# Utilizing machine learning models for predicting outcomes in acute pancreatitis: development and validation in three retrospective cohorts

**DOI:** 10.1186/s12911-025-03103-7

**Published:** 2025-07-11

**Authors:** Kaier Gu, Yang Liu

**Affiliations:** 1 Department of Internal Medicine, Shaoxing Maternity and Child Health Care Hospital, Shaoxing, Zhejiang China; 2 Department of Gastroenterology, The First Affiliated Hospital of Wenzhou Medical University, Wenzhou, Zhejiang China

**Keywords:** Acute pancreatitis, Machine learning, Prediction model, In-hospital mortality, MIMIC, eICU

## Abstract

**Background:**

Acute pancreatitis (AP) is associated with a high readmission rate; however, there is a paucity of models capable of predicting post-discharge outcomes. Furthermore, existing in-hospital prediction models exhibit notable limitations. This study leverages machine learning (ML) technology to develop prognosis prediction models for AP patients, encompassing in-hospital mortality, readmission rates, and post-discharge mortality.

**Methods:**

A retrospective analysis was carried out on the clinical and laboratory data of AP patients from three databases (MIMIC database, eICU database, and Wenzhou Hospital in China), and they were divided into a training set and two validation sets. In the training set, key variables were screened using univariate logistic regression and the LASSO method. Six ML algorithms were employed to construct predictive models. The performance of these models was appraised using receiver operating characteristic curves, decision curve analysis, Shapley additive explanations plots, and other relevant metrics. A comparison was made between the predictive capabilities of the ML models and clinical scores. Subsequently, the performance of the machine learning models was subjected to further validation within two external validation sets.

**Results:**

A total of 2,559 AP patients were included. There were 12–26 variables selected for model training. Among the six ML models under assessment, the Logistic Regression, Random Forest, and eXtreme Gradient Boosting (XGB) models exhibited relatively superior performance in predicting in-hospital mortality, mortality within 180/365 days after discharge. Findings from the decision curve analysis and two external validation sets further indicated that the XGB model exhibited the optimal performance in predicting the in-hospital mortality of AP patients admitted to the intensive care unit. Specifically, the XGB model demonstrated stability in the area under the curve across different centers, achieved a balance between sensitivity and specificity, and effectively prevented overfitting through regularization mechanisms. These features are highly congruent with the core requirements for robustness in the medical context.

**Conclusions:**

By collecting the dynamic variables of patients during their hospitalization and establishing an XGB model, it is conducive to identifying the short-term and long-term prognoses of AP patients and promoting the decision-making of clinicians.

**Clinical trial number:**

Not applicable.

**Supplementary Information:**

The online version contains supplementary material available at 10.1186/s12911-025-03103-7.

## Background

Acute pancreatitis (AP) is a common gastrointestinal condition, which leads to local or systemic inflammation of the pancreas [1, 2]. The occurrence of AP has been consistently increasing [3], with an estimated average annual increase of approximately 3% globally from 1961 to 2016 [4]. Approximately 20% of AP patients may progress spontaneously to severe forms of the disease, accompanied by systemic inflammatory response syndrome, infection, and persistent organ failure; this subgroup exhibits a mortality rate ranging from 20–40% [5, 6]. Rehospitalization following discharge is a prevalent concern among patients with AP after their initial episode, with readmission rates differing based on the underlying etiology and healthcare facility. Previous studies have pointed out that the readmission rate for all-cause pancreatitis ranges from 7–34% [7]. Additionally, a multi-center database analysis pointed out that the 90-day mortality rate of AP patients after discharge was nearly equivalent to the in-hospital mortality rate, emphasizing that future research protocols for AP must incorporate a follow-up period of at least 90 days after discharge [8].

Currently, several widely utilized clinical scoring systems exist to predict unfavorable outcomes in AP, including the Systemic Inflammatory Response Syndrome Criteria (SIRS), Bedside Index of Severity in Acute Pancreatitis, and Acute Physiology And Chronic Health Evaluation (APACHE) II [9–11]. Additionally, laboratory indicators can also serve as predictive tools, such as white blood cell count, serum calcium, and albumin [12–14]. Recently, an increasing amount of research has concentrated on creating prognostic models aimed at forecasting the outcomes of AP. For instance, Gao et al. developed a nomogram encompassing six clinical indicators to predict the severity of patients with AP [15]. Li et al. established a predictive model comprising seven clinical indicators [16]. However, traditional scoring systems have unavoidable limitations, and predictive models based on these systems and parameters show limited accuracy. Thus, the development of a novel predictive scoring system with enhanced accuracy, which improves the reliability of AP classification, holds substantial clinical significance.

In recent years, the use of artificial intelligence in biomedicine has expanded. Machine learning (ML) techniques derived from electronic health records have gained clinical attention and approval. A considerable number of ML techniques have been employed for the construction of predictive models, demonstrating outstanding predictive value [17, 18]. Various ML algorithms possess distinct characteristics and limitations, making them suitable for different situations. There is no single ML algorithm that serves as the optimal choice in every scenario. Qian et al. suggest that ML has demonstrated exceptional accuracy in predicting the severity of AP, thereby furnishing a valuable reference for the enhancement or development of straightforward clinical prediction tools [19]. Furthermore, numerous studies have employed ML models to create predictive frameworks for various clinical endpoints in AP patients, including mortality, organ failure, and disease recurrence [20], which highlight the impressive performance of ML in forecasting diverse outcomes associated with AP [21].

Nevertheless, the existing predictive studies focusing on the follow-up of AP patients after discharge remain scarce. Moreover, there is an even greater paucity of predictive models specifically addressing short-term readmission and post-discharge mortality among these patients. Therefore, this research is intended to establish a series of ML models for forecasting the prognosis of AP patients, encompassing not only the mortality rate of hospitalization but also short-term readmission and mortality rates after discharge, in order to offer effective decision-making support tools for clinicians.

## Methods

### Data source

This study is intended to initially explore the predictive efficacy of ML models for the prognosis of AP (an exploratory research design). Such analyses typically aim at maximizing the utilization of the available data. As there is a lack of prior reports on the effect size of AP prediction models, traditional sample size calculations are hard to carry out. Hence, all cases in the database that met the inclusion criteria were included.

This study primarily utilized three data sources: MIMIC-IV (v3.0) database, eICU database, and the electronic medical record data in the First Affiliated Hospital of Wenzhou Medical University (hereafter referred to as Wenzhou Hospital).

#### MIMIC-IV (v3.0) database

A publicly multi-parameter intensive care database. This database encompasses information on all patients admitted to Beth Israel Deaconess Medical Center between 2008 and 2022. To safeguard patient privacy, all personal identifiers were anonymized.

#### eICU database

A de-identified multi-center resource that contains hospitalization data for ICU patients across 208 hospitals in the United States. Notably, there is no overlap in hospital participation between the MIMIC and eICU databases.

#### Wenzhou hospital

Electronic medical record data of adult AP patients admitted to the intensive care unit (ICU) of Wenzhou Hospital from January 2016 to December 2023 were collected.

The research was carried out in accordance with the guidelines set forth in the Declaration of Helsinki. Approval for this study was obtained from the Ethics Committee at the First Affiliated Hospital of Wenzhou Medical University. This was a retrospective, observational cohort study, and the data had been anonymized and pooled prior to access and analysis. Therefore, the ethics committee waived the informed consent by all participants.

### Inclusion and exclusion criteria

We extracted patients from the MIMIC database who were diagnosed with AP, identified by an ICD-9 diagnosis code of “5770” or an ICD-10 diagnosis code of “K85**” (** can represent any digit from 0 to 9).

Exclusion criteria included: (1) Patients who were not first-time admissions; (2) Patients under the age of 18; (3) Patients whose basic information was incomplete or where more than 20% of data was missing.

**Fig. 1:**
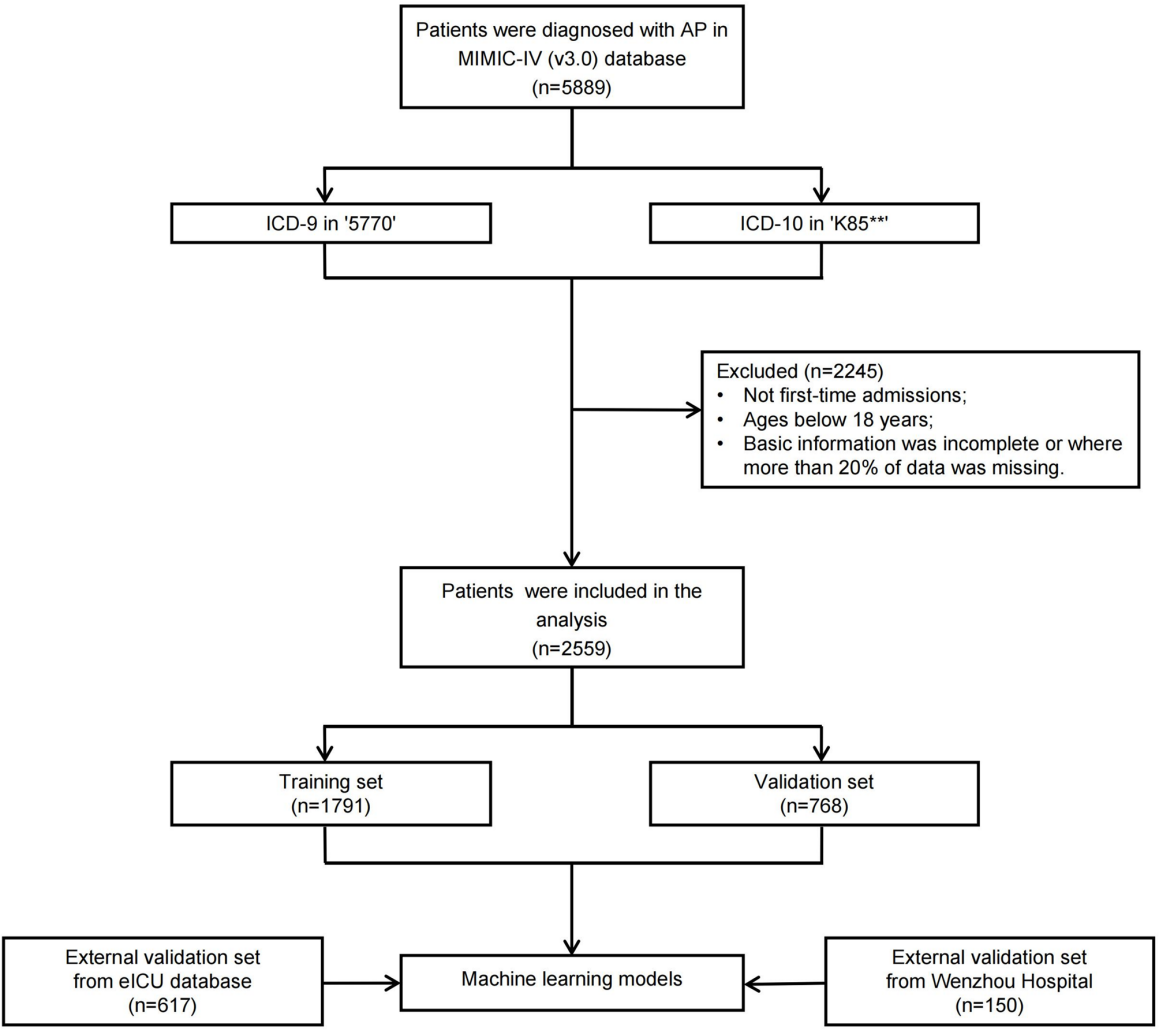
Flow chart of patient inclusion and exclusion. AP, acute pancreatitis

Patients from both the eICU database and Wenzhou Hospital with a diagnosis of AP met the extraction criteria established for MIMIC-IV. The selection process is illustrated in Fig. 1.

### Data collection

Identify potential variables for predictive modeling, including demographic characteristics, surgical history, pre-existing health conditions, laboratory indicators, and additional information. For laboratory indicators evaluated several times during hospitalization, gather their maximum, average, and minimum values. For patients admitted to the ICU specifically, collect variables: sepsis; acute kidney injury (AKI); vital signs recorded on the first day of ICU admission; and severity scores. Comprehensive descriptions of all variables can be found in Table S1. Extract the same variables from the different databases. Detailed information regarding these unit conversions is available in Table S2.

### Outcome indicators

This study presents six clinical endpoints, namely the in-hospital mortality rate, the rate of readmission within 30/60/90 days after discharge, the mortality rate within 180/365 days after discharge.

The definition of in-hospital mortality rate is the proportion of patients who die for various causes during their hospital stay among all admitted patients. The definition of readmission within 30 days after discharge is the proportion of patients who are readmitted for AP-related treatment within 30 days from the date of discharge among all discharged patients. Similarly, the rate of readmission within 60/90 days after discharge respectively refer to the proportion of readmitted patients within the corresponding time ranges. The definition of the mortality rate within 180 days after discharge is the proportion of patients who die for various reasons within 180 days from the date of discharge among all discharged patients. Likewise, the mortality rate within 365 days after discharge refers to the proportion of patients who die within 365 days from the date of discharge among all discharged patients.

### Construction and validation of the model

The AP patients in the MIMIC database were randomly divided into the training set and the internal validation set at a ratio of 7:3. The training set was utilized for developing models with diverse ML algorithms. The internal validation set and two external validation sets were employed to assess the performance of these models.

In the training set, univariate logistic regression is initially adopted to screen variables with P-values < 0.1. Subsequently, using Least Absolute Shrinkage and Selection Operator (LASSO) regression analysis to obtain final variables closely associated with the predicted outcomes.

These variables were input into six ML algorithm models for model construction, including K-Nearest Neighbor (KNN), Light Gradient Boosting Machine (LGBM), Logistic Regression (LR), Random Forest (RF), Support Vector Machine (SVM), eXtreme Gradient Boosting (XGB). To mitigate overfitting and enhance model stability, a ten-fold cross-validation approach is implemented.

The discriminatory capacity of the model was primarily assessed using the receiver operating characteristic (ROC) curve and the area under the curve (AUC) of the ROC in the validation set. Moreover, various performance metrics were analyzed, including accuracy, sensitivity, specificity, positive predictive value, negative predictive value, and F1 score. Compare the predictive performance of ML models and clinical scores [Acute Physiology Score III (APS III), Simplified Acute Physiology Score II (SAPS II), SIRS, Sequential Organ Failure Assessment (SOFA)]. To further assess the clinical effectiveness of the model, we utilized decision curve analysis. To quantify the contribution of individual variables to model predictions, we utilized SHapley Additive exPlanations (SHAP) method.

### Data processing

#### Identification and treatment of extreme/outlier values

Based on medical knowledge, establish reasonable ranges for physiological parameters. For clearly erroneous data, they shall be deleted. For values with excessively large differences in adjacent records within a short period of time, the original medical records shall be retraced for verification and correction.

#### Filling of missing values

Exclude variables with missing values exceeding 20%. For categorical variables with missing values below 20%, the mode method is utilized to impute the missing data. For continuous variables with missing values under 20%, the median method is utilized to impute the missing data. The missing rate for each variable is shown in the Table S3.

### Statistical analysis

The statistical analyses and model construction and validation were performed via R software package (version 4.2.1) and DCPM (V4.01,Jingding Medical Technology Co., Ltd.).

Quantitative data include age, hospital and ICU stay durations, laboratory indicators, and ICU-specific variables (first-day vital signs and severity scores). Qualitative data include gender, surgical history, pre-existing health conditions (hypertension, diabetes), and ICU-specific variables (sepsis, AKI).

The normality of continuous variables is evaluated using Kolmogorov-Smirnov test. Continuous variables that follow a normal distribution are expressed as the mean ± standard deviation. Continuous variables that do not follow a normal distribution are expressed as the median and interquartile range.

Categorical variables are expressed as frequency and percentage.

Homogeneity of variance for continuous variables was tested by Levene’s test (P-values > 0.05 were considered homogeneous); For categorical variables, the chi-square test required that the expected frequency of all cells be ≥ 5; otherwise, Fisher’s exact test was used.

P-values < 0.05 are regarded as statistically significant.

## Results

### Characteristics of patients

Based on the inclusion criteria, 2559 patients were finally included. Table 1 presented the basic information. Table 2 analyzes factors leading to different outcomes of all the patients. Table 3 demonstrates the following findings: Compared to all patients, those readmitted within 30/60/90 days after discharge showed no significant difference in mortality. This lack of difference was also observed in mortality within 180/365 days after discharge. Table 4 presents different outcomes corresponding to different patient categories.

**Table 1:** Baseline characteristics of patients

Characteristics	n (%), mean ± SD (range), or median [IQR]			P-value		
	All the patients (n = 2559)	Patients underwent operations (n = 1091)	Patients admitted to the ICU (n = 636)	Between all the patients and patients underwent operations	Between all the patients and patients admitted to the ICU	Between patients underwent operations and patients underwent operations
Age (years)	57.00 [44.00–70.00]	64.00 [49.00–76.00]	59.50 [46.00–74.00]	< 0.001	0.001	0.006
Gender (male), n (%)	1305 (51.00%)	493 (45.19%)	366 (57.55%)	0.001	0.004	< 0.001
Length of stay in hospital (days)	4.62 [2.70–8.72]	4.88 [3.09–8.21]	11.25 [5.96–19.86]	0.005	< 0.001	< 0.001
Length of stay in ICU (hours)	0.00 [0.00–0.00]	0.00 [0.00–0.00]	67.51 [34.25-160.75]	0.003	< 0.001	< 0.001
Death in hospital, n (%)	82 (3.20%)	26 (2.38%)	76 (11.95%)	0.217	< 0.001	< 0.001
Re-admission within 30 days after discharge, n (%)	222 (8.68%)	78 (7.15%)	39 (6.96%)	0.141	0.044	0.476
Re-admission within 60 days after discharge, n (%)	288 (11.25%)	105 (9.62%)	56 (8.81%)	0.163	0.087	0.632
Re-admission within 90 days after discharge, n (%)	322 (12.58%)	114 (10.45%)	65 (10.22%)	0.078	0.117	0.945
Follow-up after discharge until death, n (%)	301 (11.76%)	150 (13.75%)	93 (14.62%)	0.106	0.058	0.666
Death within 180 days after discharge, n (%)	109 (4.26%)	53 (4.86%)	50 (7.86%)	0.474	< 0.001	0.015
Death within 365 days after discharge, n (%)	154 (6.02%)	82 (7.52%)	62 (9.75%)	0.107	0.001	0.126
Surgical operations, n (%)	425 (16.61%)	425 (38.96%)	69 (10.85%)	< 0.001	< 0.001	< 0.001
Endoscopy, n (%)	852 (33.29%)	852 (78.09%)	191 (30.03%)	< 0.001	0.128	< 0.001
Interventional operations, n(%)	815 (31.85%)	815 (74.70%)	178 (27.99%)	< 0.001	0.067	< 0.001
Surgical or interventional or endoscopic operations, n (%)	1091 (42.63%)	1091 (100%)	227 (35.69%)	< 0.001	0.002	< 0.001
Endoscopy combined with surgical operations, n (%)	196 (7.66%)	196 (17.97%)	35 (5.50%)	< 0.001	0.073	< 0.001
Endoscopy combined with interventional operations, n (%)	803 (31.38%)	803 (73.60%)	176 (27.67%)	< 0.001	0.077	< 0.001
Surgical or interventional operations other than endoscopy, n (%)	239 (9.34%)	239 (21.91%)	36 (5.66%)	< 0.001	0.004	< 0.001
Endoscopic, surgical, and interventional procedures were performed during a single hospitalization, n (%)	193 (7.54%)	193 (17.69%)	35 (5.50%)	< 0.001	0.089	< 0.001
Hypertension, n (%)	1082 (42.28%)	476 (43.63%)	302 (47.48%)	0.473	0.020	0.133
Diabetes, n (%)	523 (20.44%)	223 (20.44%)	172 (27.04%)	1.000	< 0.001	0.002
Anion gap _ max (mEq/L)	16.00 [14.00–18.00]	16.00 [14.00–18.00]	19.00 [16.00–22.00]	0.471	< 0.001	< 0.001
Anion gap _ avg (mEq/L)	13.50 [12.10–15.00]	13.50 [12.25–14.81]	13.98 [12.50-15.86]	0.465	< 0.001	< 0.001
Anion gap _ min (mEq/L)	11.00 [10.00–13.00]	11.00 [10.00–13.00]	10.00 [9.00–12.00]	0.507	< 0.001	< 0.001
Bicarbonate _ max (mEq/L)	28.00 [26.00–30.00]	28.00 [26.00–30.00]	29.00 [26.00–32.00]	0.821	< 0.001	< 0.001
Bicarbonate _ avg (mEq/L)	25.12 [23.29-27.00]	25.12 [23.46–26.81]	24.17 [21.77–26.25]	0.759	< 0.001	< 0.001
Bicarbonate _ min (mEq/L)	23.00 [20.00–25.00]	23.00 [20.00–25.00]	19.00 [15.00–22.00]	0.568	< 0.001	< 0.001
Chloride _ max (mEq/L)	106.00 [104.00-109.00]	106.00 [104.00-109.00]	109.50 [105.00-114.00]	0.725	< 0.001	< 0.001
Chloride _ avg (mEq/L)	103.50 [101.00-106.00]	103.67 [101.33-105.92]	103.32 [100.45-106.32]	0.052	0.857	0.140
Chloride _ min (mEq/L)	101.00 [97.00-103.00]	101.00 [98.00-104.00]	97.00 [93.00-101.00]	0.004	< 0.001	< 0.001
Creatinine _ max (mg/dL)	0.90 [0.70–1.20]	0.90 [0.70–1.20]	1.30 [0.90–2.60]	0.952	< 0.001	< 0.001
Creatinine _ avg (mg/dL)	0.78 [0.63-1.00]	0.78 [0.63-1.00]	0.93 [0.67–1.54]	0.964	< 0.001	< 0.001
Creatinine _ min (mg/dL)	0.70 [0.50–0.90]	0.70 [0.60–0.80]	0.70 [0.50–0.92]	0.915	0.983	0.917
Glucose _ max (mg/dL)	131.00 [105.00-182.75]	130.00 [106.00-168.00]	194.50 [144.00-277.50]	0.145	< 0.001	< 0.001
Glucose _ avg (mg/dL)	106.50 [92.14-128.32]	104.20 [91.71-122.82]	125.10 [106.50-157.53]	0.011	< 0.001	< 0.001
Glucose _ min (mg/dL)	84.00 [73.00–96.00]	83.00 [71.00–94.00]	83.00 [72.00–96.00]	0.005	0.275	0.292
Potassium _ max (mEq/L)	4.30 [4.00-4.70]	4.20 [4.00-4.60]	4.80 [4.30–5.30]	0.017	< 0.001	< 0.001
Potassium _ avg (mEq/L)	3.90 [3.70–4.13]	3.88 [3.68–4.10]	3.92 [3.73–4.17]	0.018	0.023	< 0.001
Potassium _ min (mEq/L)	3.50 [3.20–3.80]	3.50 [3.20–3.70]	3.20 [3.00-3.50]	0.283	< 0.001	< 0.001
Sodium _ max (mEq/L)	141.00 [139.00-143.00]	141.00 [139.00-143.00]	143.00 [140.00-147.25]	0.698	< 0.001	< 0.001
Sodium _ avg (mEq/L)	139.00 [137.00-141.00]	139.00 [137.33-140.87]	138.75 [136.50-141.15]	0.125	0.213	0.028
Sodium _ min (mEq/L)	137.00 [134.00-139.00]	137.00 [134.00-139.00]	134.00 [131.00-136.00]	0.011	< 0.001	< 0.001
Urea nitrogen _ max (mg/dL)	14.00 [10.00–24.00]	15.00 [11.00–24.00]	28.00 [16.00–56.00]	0.092	< 0.001	< 0.001
Urea nitrogen _ avg (mg/dL)	11.00 [7.61–17.39]	11.50 [8.18–17.83]	17.73 [10.55–33.74]	0.033	< 0.001	< 0.001
Urea nitrogen _ min (mg/dL)	8.00 [5.00–12.00]	8.00 [6.00–12.00]	9.00 [6.00–15.00]	0.015	< 0.001	0.005
Hematocrit _ max (%)	37.20 [34.00-40.70]	37.20 [34.25–40.35]	36.60 [33.10–40.80]	0.825	0.068	0.067
Hematocrit _ avg (%)	34.27 [30.52–37.49]	34.27 [30.85–37.30]	30.32 [27.50–34.20]	0.929	< 0.001	< 0.001
Hematocrit _ min (%)	32.10 [27.60-35.75]	32.10 [28.10–35.40]	26.00 [21.98–30.90]	0.736	< 0.001	< 0.001
Hemoglobin _ max (g/dL)	12.40 [11.20–13.60]	12.40 [11.30–13.50]	12.20 [10.90-13.62]	0.711	0.018	0.051
Hemoglobin _ avg (g/dL)	11.45 [10.10–12.60]	11.45 [10.18–12.41]	10.08 [8.99–11.41]	0.769	< 0.001	< 0.001
Hemoglobin _ min (g/dL)	10.70 [9.10–12.00]	10.70 [9.30–11.90]	8.50 [7.20–10.20]	0.523	< 0.001	< 0.001
MCH _ max (pg)	30.90 [29.50–32.40]	30.90 [29.50–32.20]	31.40 [30.20–33.30]	0.123	< 0.001	< 0.001
MCH _ avg (pg)	30.35 [29.00-31.80]	30.27 [29.00-31.64]	30.44 [29.24–32.17]	0.104	0.037	0.002
MCH _ min (pg)	29.80 [28.40–31.30]	29.80 [28.40–31.10]	29.70 [28.20-31.13]	0.142	0.067	0.529
MCHC _ max (g/dL)	34.10 [33.10–35.00]	34.01 (1.32)	34.30 [33.48–35.40]	0.156	< 0.001	< 0.001
MCHC _ avg (g/dL)	33.30 [32.48–34.14]	33.28 [32.45–34.03]	33.06 [32.30-33.84]	0.103	< 0.001	0.002
MCHC _ min (g/dL)	32.60 [31.60–33.50]	32.50 [31.60–33.40]	31.80 [30.80–32.60]	0.138	< 0.001	< 0.001
MCV _ max (fL)	92.00 [88.00–97.00]	92.00 [89.00–96.00]	95.00 [91.00-101.00]	0.525	< 0.001	< 0.001
MCV _ avg (fL)	91.00 [87.00–95.00]	90.80 [87.50-94.18]	92.10 [88.29–96.76]	0.456	< 0.001	< 0.001
MCV _ min (fL)	89.00 [86.00–93.00]	89.00 [86.00–93.00]	89.00 [86.00–93.00]	0.637	0.825	0.622
PLT _ max (K/µL)	263.00 [198.00-364.00]	263.00 [200.00-343.00]	341.00 [214.75-536.25]	0.618	< 0.001	< 0.001
PLT _ avg (K/µL)	226.86 [171.43-296.25]	226.86 [178.00-288.50]	231.53 [153.00-327.84]	0.942	0.814	0.886
PLT _ min (K/µL)	188.00 [135.00-244.00]	190.00 [145.50-243.50]	136.00 [92.75–191.00]	0.155	< 0.001	< 0.001
RDW _ max (%)	14.10 [13.20–15.60]	14.10 [13.30–15.50]	15.60 [14.30–17.60]	0.971	< 0.001	< 0.001
RDW _ avg (%)	13.83 [13.05-15.00]	13.83 [13.07–14.95]	14.80 [13.85–16.34]	0.981	< 0.001	< 0.001
RDW _ min (%)	13.50 [12.80–14.50]	13.50 [12.90–14.50]	14.00 [13.20–15.10]	0.823	< 0.001	< 0.001
RBC _ max (m/µL)	4.11 [3.72–4.52]	4.11 [3.75–4.50]	4.00 [3.53–4.48]	0.689	< 0.001	< 0.001
RBC _ avg (m/µL)	3.79 [3.35–4.19]	3.79 [3.39–4.14]	3.32 [2.93–3.76]	0.741	< 0.001	< 0.001
RBC _ min (m/µL)	3.56 [3.01-4.00]	3.56 [3.10–3.94]	2.81 [2.37–3.42]	0.948	< 0.001	< 0.001
WBC _ max (K/µL)	11.00 [7.70-16.48]	11.50 [8.15–16.60]	17.50 [12.60-24.33]	0.059	< 0.001	< 0.001
WBC _ avg (K/µL)	8.80 [6.51–12.03]	8.95 [6.74–12.07]	11.64 [8.57–15.13]	0.122	< 0.001	< 0.001
WBC _ min (K/µL)	6.60 [5.00-8.90]	6.60 [5.20–8.70]	6.90 [5.10–9.33]	0.335	0.199	0.589
TBil _ max (mg/dL)	1.10 [0.60-3.00]	1.80 [0.80–4.60]	1.70 [0.80–4.60]	< 0.001	< 0.001	0.650
TBil _ avg (mg/dL)	0.85 [0.50–1.81]	1.20 [0.65–2.64]	1.05 [0.56–2.24]	< 0.001	< 0.001	0.011
TBil _ min (mg/dL)	0.60 [0.40–1.10]	0.70 [0.40–1.40]	0.60 [0.30–1.10]	< 0.001	0.149	< 0.001
ALT _max (IU/L)	77.00 [30.00-222.00]	145.00 [63.00-312.00]	86.00 [43.00-217.25]	< 0.001	0.002	< 0.001
ALT _avg (IU/L)	53.68 [24.00-144.39]	95.50 [43.86-208.33]	52.85 [28.08-111.88]	< 0.001	0.965	< 0.001
ALT _ min (IU/L)	34.00 [17.00–82.00]	56.00 [26.00-123.00]	27.00 [15.00–46.00]	< 0.001	< 0.001	< 0.001
ALP _ max (IU/L)	126.00 [80.50-216.50]	164.00 [108.00-275.00]	146.50 [97.00-247.00]	< 0.001	< 0.001	0.007
ALP _ avg (IU/L)	107.58 [73.17-173.35]	135.50 [92.17–218.50]	107.58 [76.25–173.00]	< 0.001	0.425	< 0.001
ALP _ min (IU/L)	88.00 [61.00-137.00]	108.00 [74.00-176.00]	73.00 [53.00-114.00]	< 0.001	< 0.001	< 0.001
AST _max (IU/L)	78.00 [33.00-189.00]	112.00 [59.00-231.00]	110.00 [54.00-283.00]	< 0.001	< 0.001	0.298
AST _avg (IU/L)	51.50 [26.00-103.26]	67.56 [39.00-121.00]	59.19 [35.58-121.94]	< 0.001	< 0.001	0.317
AST _min (IU/L)	29.00 [19.00–50.00]	33.00 [21.00–57.00]	28.00 [19.00–42.00]	< 0.001	0.139	< 0.001
Magnesium _ max (mg/dL)	2.10 [1.90–2.40]	2.10 [1.90–2.30]	2.40 [2.20–2.70]	0.150	< 0.001	< 0.001
Magnesium _ avg (mg/dL)	1.93 [1.80–2.05]	1.93 [1.80–2.02]	1.99 [1.89–2.11]	0.173	< 0.001	< 0.001
Magnesium _ min (mg/dL)	1.70 [1.50–1.90]	1.70 [1.60–1.90]	1.60 [1.50–1.80]	0.739	< 0.001	< 0.001
Calcium _ max (mg/dL)	9.00 [8.60–9.30]	8.90 [8.60–9.20]	9.00 [8.60–9.50]	0.015	0.049	0.001
Calcium _ avg (mg/dL)	8.55 [8.17–8.90]	8.55 [8.20–8.86]	8.20 [7.85–8.57]	0.860	< 0.001	< 0.001
Calcium _ min (mg/dL)	8.20 [7.60–8.60]	8.20 [7.80–8.60]	7.40 [6.80–7.90]	0.120	< 0.001	< 0.001
Phosphate _ max (mg/dL)	3.80 [3.20–4.50]	3.70 [3.20–4.30]	4.60 [3.80–5.90]	0.004	< 0.001	< 0.001
Phosphate _ avg (mg/dL)	3.15 [2.70–3.66]	3.15 [2.70–3.47]	3.24 [2.73–3.80]	0.012	0.004	< 0.001
Phosphate _ min (mg/dL)	2.50 [1.90–3.10]	2.50 [2.00-2.90]	1.90 [1.40–2.50]	0.655	< 0.001	< 0.001
SIRS score	-	-	3.00 [2.00–4.00]	-	-	-
SOFA score	-	-	5.00 [3.00–8.00]	-	-	-
Sepsis, n (%)	-	-	407 (63.99%)	-	-	-
AKI, n (%)	-	-	430 (67.61%)	-	-	-
Duration of auxiliary ventilation (hours)	-	-	0.00 [0.00-40.11]	-	-	-
Heart rate on the first day of ICU _ max (beats/minute)	-	-	93.68 [80.95-107.04]	-	-	-
Heart rate on the first day of ICU _ avg (beats/minute)	-	-	114.00 [97.00-128.00]	-	-	-
Heart rate on the first day of ICU _ min (beats/minute)	-	-	79.00 [67.00–92.00]	-	-	-
SysBP on the first day of ICU _ max (mmHg)	-	-	121.00 [110.17-134.47]	-	-	-
SysBP on the first day of ICU _ avg (mmHg)	-	-	147.00 [133.00-163.00]	-	-	-
SysBP on the first day of ICU _ min (mmHg)	-	-	98.00 [87.75–111.00]	-	-	-
DiasBP on the first day of ICU _ max (mmHg)	-	-	68.23 [60.48–77.45]	-	-	-
DiasBP on the first day of ICU _ avg (mmHg)	-	-	91.00 [79.00-104.00]	-	-	-
DiasBP on the first day of ICU _ min (mmHg)	-	-	52.00 [44.00–62.00]	-	-	-
Mean arterial pressure on the first day of ICU admission _ max (mmHg)	-	-	81.14 [72.69–90.69]	-	-	-
Mean arterial pressure on the first day of ICU admission _ avg (mmHg)	-	-	102.00 [91.00-116.00]	-	-	-
Mean arterial pressure on the first day of ICU admission _ min (mmHg)	-	-	65.00 [55.00–74.00]	-	-	-
Respiratory rate on the first day of ICU _ max (beats/mins)	-	-	20.44 [17.63–23.55]	-	-	-
Respiratory rate on the first day of ICU _ avg (beats/mins)	-	-	29.00 [25.00–34.00]	-	-	-
Respiratory rate on the first day of ICU _ min (beats/mins)	-	-	14.00 [12.00–16.00]	-	-	-
Temperature on the first day of ICU admission _ max(℃)	-	-	36.97 [36.71–37.34]	-	-	-
Temperature on the first day of ICU admission _ avg (℃)	-	-	37.44 [37.06–38.11]	-	-	-
Temperature on the first day of ICU admission _ min (℃)	-	-	36.56 [36.22–36.83]	-	-	-
SpO_2_ on the first day of ICU _ max (%)	-	-	96.12 [94.71–97.56]	-	-	-
SpO_2_ on the first day of ICU _ avg (%)	-	-	100.00 [98.00-100.00]	-	-	-
SpO_2_ on the first day of ICU _ min (%)	-	-	92.00 [90.00–94.00]	-	-	-

SD, standard deviation; IQR, inter-quartile range; ICU, intensive care unit; MCH, mean corpuscular hemoglobin; MCHC, mean corpuscular hemoglobin concentration; MCV, mean corpuscular volume; PLT, platelet count; RDW, red cell distribution width; RBC, red blood cells; WBC, white blood cells; TBil, total bilirubin; ALT, alanine aminotransferase; ALP, alkaline phosphatase; AST, aspartate aminotransferase; SIRS, systemic inflammatory response syndrome; SOFA, sepsis-related organ failure; AKI, acute kidney injury; SysBP, systolic blood pressure; DiasBP, diastolic blood pressure; SpO_2_, saturation of peripheral oxygen

**Table 2:** Analysis of factors leading to different outcomes of all the patients (n = 2559)

Factors	Outcomes					
	Death in hospital P-value	Re-admission within 30 days after discharge P-value	Re-admission within 60 days after discharge P-value	Re-admission within 90 days after discharge P-value	Death within 180 days after discharge P-value	Death within 365 days after discharge P-value
Age	< 0.001	< 0.001	< 0.001	< 0.001	< 0.001	< 0.001
Gender	0.706	0.748	0.407	0.178	0.987	0.510
Length of stay in hospital	< 0.001	0.001	< 0.001	< 0.001	< 0.001	< 0.001
Length of stay in ICU	< 0.001	0.012	0.037	0.065	< 0.001	< 0.001
Surgical operations	0.065	0.007	0.001	< 0.001	0.012	0.013
Endoscopy	0.105	0.064	0.131	0.063	0.034	< 0.001
Interventional operations	0.067	0.032	0.101	0.054	0.013	< 0.001
Surgical or interventional or endoscopic operations	0.055	0.022	0.029	0.006	0.233	0.008
Endoscopy combined with surgical operations	0.044	0.006	0.001	0.001	0.157	0.179
Endoscopy combined with interventional operations	0.080	0.032	0.083	0.046	0.03	< 0.001
Surgical or interventional operations other than endoscopy	0.655	0.435	0.246	0.121	0.216	0.267
Endoscopic, surgical, and interventional procedures were performed during a single hospitalization	0.046	0.006	0.002	0.001	0.168	0.195
Hypertension	0.969	0.278	0.328	0.259	0.283	0.816
Diabetes	0.015	0.170	0.195	0.169	0.013	0.023
Anion gap _ max	< 0.001	0.601	0.932	0.610	< 0.001	< 0.001
Anion gap _ avg	< 0.001	0.059	0.017	0.006	0.001	0.006
Anion gap _ min	0.802	0.004	< 0.001	< 0.001	0.194	0.074
Bicarbonate _ max	0.007	< 0.001	< 0.001	< 0.001	0.252	0.668
Bicarbonate _ avg	< 0.001	< 0.001	< 0.001	< 0.001	< 0.001	< 0.001
Bicarbonate _ min	< 0.001	0.080	0.116	0.101	< 0.001	< 0.001
Chloride _ max	< 0.001	0.421	0.546	0.631	< 0.001	< 0.001
Chloride _ avg	0.205	0.004	0.005	0.005	0.063	0.363
Chloride _ min	< 0.001	0.003	0.001	< 0.001	0.156	0.026
Creatinine _ max	< 0.001	0.245	0.171	0.045	< 0.001	< 0.001
Creatinine _ avg	< 0.001	0.073	0.02	0.003	< 0.001	< 0.001
Creatinine _ min	< 0.001	0.024	0.002	< 0.001	< 0.001	0.004
Glucose _ max	< 0.001	0.390	0.065	0.103	< 0.001	< 0.001
Glucose _ avg	< 0.001	0.278	0.054	0.067	0.001	0.002
Glucose _ min	0.532	0.757	0.943	0.732	0.022	0.039
Potassium _ max	< 0.001	0.017	0.003	0.001	< 0.001	< 0.001
Potassium _ avg	< 0.001	0.024	0.004	0.003	0.716	0.745
Potassium _ min	< 0.001	0.591	0.644	0.978	< 0.001	< 0.001
Sodium _ max	< 0.001	0.788	0.535	0.663	0.001	0.004
Sodium _ avg	0.476	0.289	0.256	0.139	0.262	0.966
Sodium _ min	< 0.001	0.131	0.018	0.007	0.004	< 0.001
Urea nitrogen _ max	< 0.001	0.673	0.626	0.421	< 0.001	< 0.001
Urea nitrogen _ avg	< 0.001	0.221	0.117	0.061	< 0.001	< 0.001
Urea nitrogen _ min	< 0.001	0.009	0.001	0.001	< 0.001	< 0.001
Hematocrit _ max	0.059	0.245	0.273	0.166	0.002	< 0.001
Hematocrit _ avg	< 0.001	0.065	0.023	0.013	< 0.001	< 0.001
Hematocrit _ min	< 0.001	0.142	0.046	0.027	< 0.001	< 0.001
Hemoglobin _ max	0.013	0.133	0.129	0.062	< 0.001	< 0.001
Hemoglobin _ avg	< 0.001	0.030	0.009	0.005	< 0.001	< 0.001
Hemoglobin _ min	< 0.001	0.061	0.022	0.013	< 0.001	< 0.001
MCH _ max	0.005	0.097	0.197	0.148	0.279	0.157
MCH _ avg	0.222	0.015	0.02	0.016	0.586	0.967
MCH _ min	0.761	0.014	0.009	0.008	0.113	0.291
MCHC _ max	0.213	0.082	0.195	0.084	0.253	0.258
MCHC _ avg	< 0.001	0.007	0.004	0.002	< 0.001	< 0.001
MCHC _ min	< 0.001	0.025	0.006	0.003	< 0.001	< 0.001
MCV _ max	< 0.001	0.607	0.837	0.776	0.001	0.001
MCV _ avg	< 0.001	0.245	0.537	0.621	0.07	0.027
MCV _ min	0.325	0.120	0.210	0.296	0.985	0.470
PLT _ max	0.195	< 0.001	< 0.001	< 0.001	0.277	0.572
PLT _ avg	< 0.001	< 0.001	< 0.001	< 0.001	0.436	0.240
PLT _ min	< 0.001	< 0.001	< 0.001	< 0.001	0.002	0.001
RDW _ max	< 0.001	0.712	0.208	0.166	< 0.001	< 0.001
RDW _ avg	< 0.001	0.900	0.605	0.513	< 0.001	< 0.001
RDW _ min	< 0.001	0.565	0.955	0.942	< 0.001	< 0.001
RBC _ max	0.009	0.417	0.329	0.152	0.001	< 0.001
RBC _ avg	< 0.001	0.213	0.060	0.029	< 0.001	< 0.001
RBC _ min	< 0.001	0.293	0.085	0.042	< 0.001	< 0.001
WBC _ max	< 0.001	0.236	0.068	0.089	0.001	0.002
WBC _ avg	< 0.001	0.093	0.087	0.125	0.021	0.117
WBC _ min	0.01	0.163	0.613	0.663	0.867	0.218
TBil _ max	< 0.001	0.008	0.035	0.006	< 0.001	< 0.001
TBil _ avg	< 0.001	0.003	0.004	< 0.001	< 0.001	< 0.001
TBil _ min	< 0.001	0.001	< 0.001	< 0.001	0.088	0.002
ALT _max	0.008	0.043	0.007	< 0.001	0.051	0.041
ALT _avg	0.375	0.005	< 0.001	< 0.001	0.715	0.527
ALT _ min	0.003	0.001	< 0.001	< 0.001	0.039	0.141
ALP _ max	< 0.001	0.980	0.719	0.578	< 0.001	< 0.001
ALP _ avg	0.199	0.579	0.793	0.240	< 0.001	< 0.001
ALP _ min	0.002	0.327	0.440	0.098	0.001	< 0.001
AST _max	< 0.001	0.035	0.011	0.001	< 0.001	< 0.001
AST _avg	< 0.001	0.003	< 0.001	< 0.001	0.007	0.002
AST _min	0.001	0.001	< 0.001	< 0.001	0.696	0.318
Magnesium _ max	< 0.001	0.648	0.721	0.530	< 0.001	< 0.001
Magnesium _ avg	< 0.001	0.186	0.637	0.426	0.014	0.127
Magnesium _ min	< 0.001	0.578	0.857	0.923	0.036	0.005
Calcium _ max	0.045	1.000	0.830	0.757	0.420	0.683
Calcium _ avg	< 0.001	0.684	0.162	0.193	0.002	0.003
Calcium _ min	< 0.001	0.744	0.201	0.142	< 0.001	< 0.001
Phosphate _ max	< 0.001	0.044	0.004	0.001	< 0.001	< 0.001
Phosphate _ avg	< 0.001	0.001	< 0.001	< 0.001	0.016	0.053
Phosphate _ min	0.012	0.242	0.395	0.229	0.016	0.029

ICU, intensive care unit; MCH, mean corpuscular hemoglobin; MCHC, mean corpuscular hemoglobin concentration; MCV, mean corpuscular volume; PLT, platelet count; RDW, red cell distribution width; RBC, red blood cells; WBC, white blood cells; TBil, total bilirubin; ALT, alanine aminotransferase; ALP, alkaline phosphatase; AST, aspartate aminotransferase

Surgical operation is one of the treatment choices for AP patients. A total of 1091 patients received various surgeries during hospitalization. Compared to all patients, those who underwent surgery during hospitalization showed no difference in in-hospital mortality. No significant difference was observed in 180-day post-discharge mortality between the two groups. However, surgical patients exhibited reduced probabilities of readmission within 30/60/90 days after discharge. Notably, they experienced an increased mortality rate within 365 days post-discharge. Table S4 analyzes factors leading to different outcomes of patients underwent operations.

**Table 3:** Analysis of whether patients re-admitted within short term will die within 180/365 days after discharge

Outcomes	Patients re-admitted within 30 days after discharge			Patients re-admitted within 60 days after discharge			Patients re-admitted within 90 days after discharge		
	Yes (n = 222)	No (n = 2255)	P-Value	Yes (n = 288)	No (n = 2189)	P-Value	Yes (n = 322)	No (n = 2155)	P-Value
Mortality within 180 days after discharge	7 (3.15%)	102 (4.52%)	0.436	8 (2.78%)	101 (4.61%)	0.202	9 (2.80%)	100 (4.64%)	0.174
Mortality within 365 days after discharge	10 (4.50%)	144 (6.39%)	0.336	13 (4.51%)	141 (6.44%)	0.253	15 (4.66%)	139 (6.45%)	0.263

A total of 636 patients were admitted to the ICU during hospitalization. Compared to all patients, patients admitted to the ICU exhibited higher in-hospital mortality. This elevated risk persisted post-discharge, with increased mortality rates at both 180/365 days. Conversely, their 30/60/90-day readmission probabilities were markedly reduced. Table S5 analyzes factors leading to different outcomes of patients admitted to the ICU.

**Table 4:** Different outcomes corresponding to various patient categories

Outcomes	All (n = 2559)	Patients underwent operations			Patients admitted to the ICU		
		Yes (n = 1091)	No (n = 1468)	P-value	Yes (n = 636)	No (n = 1923)	P-value
In-hospital mortality	82 (3.20%)	26 (2.38%)	56 (3.81%)	0.055	76 (11.95%)	6 (0.31%)	< 0.001
Re-admission within 30 days after discharge	222 (8.68%)	78 (7.15%)	144 (9.81%)	0.022	39 (6.13%)	183 (9.52%)	0.011
Re-admission within 60 days after discharge	288 (11.25%)	105 (9.62%)	183 (12.47%)	0.029	56 (8.81%)	232 (12.06%)	0.029
Re-admission within 90 days after discharge	322 (12.58%)	114 (10.45%)	208 (14.17%)	0.006	65 (10.22%)	257 (13.36%)	0.045
Mortality within 180 days after discharge	109 (4.26%)	53 (4.86%)	56 (3.81%)	0.233	50 (7.86%)	59 (3.07%)	< 0.001
Mortality within 365 days after discharge	154 (6.02%)	82 (7.52%)	72 (4.90%)	0.008	62 (9.75%)	92 (4.78%)	< 0.001

ICU, intensive care unit

### Characteristic selection

A total of 112 clinical variables were enumerated and classified in accordance with the data distribution types (Table S1). Firstly, a univariate logistic regression analysis was conducted for all variables and the 6 outcomes. The variables with P-values < 0.1 were documented in Table S6-8. Subsequently, LASSO regression analysis was carried out on these variables to screen out the final variables (Table S9).

The study discovered that among all patients, a total of 25 variables were closely associated with in-hospital mortality. Similarly, 12–26 variables were closely associated with other five clinical endpoints. Likewise, variable screening was performed for patients undergoing surgery during hospitalization and those admitted to the ICU.

### Comparison of the performance of models

A total of six ML models were utilized to prognosticate the six clinical endpoints of AP patients. The performance of the ML models in predicting the outcomes is elaborated in Table S10-27.

#### ML models among all patients

Among all patients, when it comes to predicting in-hospital mortality, mortality within 180/365 days after discharge, the LR, RF, and XGB models exhibited outstanding performance (AUC > 0.800). When it comes to predicting readmission within 30/60/90 days after discharge, the performances of the six ML models were all suboptimal (AUC < 0.700).

#### ML models among patients underwent surgery during hospitalization

Among patients who underwent surgery during hospitalization, when it comes to predicting in-hospital mortality, mortality within 180/365 days after discharge, the LGBM, LR, RF, and XGB models displayed excellent performance (AUC > 0.800). When predicting the re-admission within 30/60/90 days after discharge, the performances of all six ML models were suboptimal (AUC < 0.700).

#### ML models among patients admitted to the ICU

Among patients admitted to the ICU, when predicting in-hospital mortality, the performances of the six ML models were all remarkable, among which the LR, RF, SVM, and XGB models demonstrate excellent performance (AUC > 0.900). When it comes to predicting readmission within 30 days after discharge, the performance of all six ML models was suboptimal (AUC < 0.700). When it comes to predicting the readmission within 60/90 days after discharge, the LR, RF, and XGB models exhibited relatively good performance (AUC > 0.700). When it comes to predicting the mortality within 180/365 days after discharge, the XGB model exhibited preferable performance (AUC > 0.700).

#### The models’ predictive performance varies for different clinical endpoints

The analysis indicated that all six ML models manifested a certain degree of predictive efficacy for predicting six clinical endpoints. The best ML models for predicting various clinical endpoints based on patient classification are summarized in Table 5. Among them, the LR, RF, and XGB models exhibited relatively superior performance in predicting in-hospital mortality, mortality within 180/365 days after discharge. However, in predicting the other three outcomes, the predictive efficacy demonstrated by the six ML models was less satisfactory in the internal validation set.

**Table 5:** Optimal machine-learning algorithms for predicting various outcomes corresponding to patient categories

Outcomes	AUC in MIMIC-IV database	AUC in eICU database	AUC in Wenzhou hospital
All-In-hospital mortality	RF (0.966)	-	-
All-Re-admission within 30 days after discharge	LR (0.592)	-	-
All-Re-admission within 60 days after discharge	XGB (0.653)	-	-
All-Re-admission within 90 days after discharge	XGB (0.664)	-	-
All-Mortality within 180 days after discharge	XGB (0.844)	-	-
All-Mortality within 365 days after discharge	XGB (0.820)	-	-
Operation-In-hospital mortality	XGB (0.930)	-	-
Operation-Re-admission within 30 days after discharge	LGBM (0.635)	-	-
Operation-Re-admission within 60 days after discharge	XGB (0.667)	-	-
Operation-Re-admission within 90 days after discharge	XGB (0.654)	-	-
Operation-Mortality within 180 days after discharge	LR (0.850)	-	-
Operation-Mortality within 365 days after discharge	RF (0.904)	-	-
ICU-In-hospital mortality	RF (0.938)	XGB (0.931)	RF (0.837)
ICU-Re-admission within 30 days after discharge	RF (0.665)	-	-
ICU-Re-admission within 60 days after discharge	RF (0.756)	-	-
ICU-Re-admission within 90 days after discharge	LGBM (0.778)	-	-
ICU-Mortality within 180 days after discharge	LR (0.745)	-	-
ICU-Mortality within 365 days after discharge	XGB (0.712)	-	-

AUC, area under the curve; ICU, intensive care unit; RF, Random Forest; LR, Logistic Regression; XGB, eXtreme Gradient Boosting; LGBM, Light Gradient Boosting Machine

### Further exploration of In-hospital mortality in ICU patients

#### Comparison of the ML models with clinical scores

**Fig. 2:**
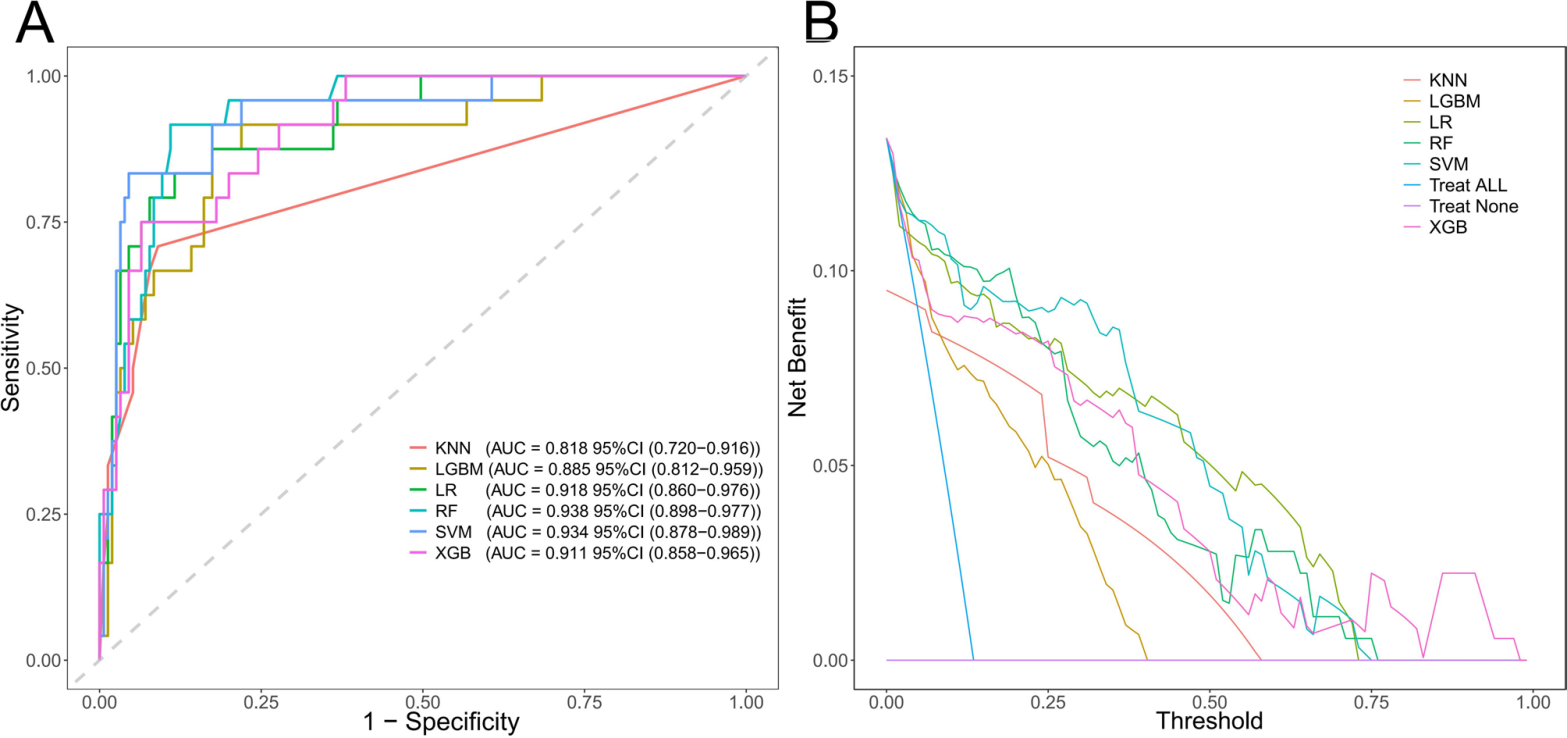
ROC curves and decision curves of the ML models in the internal validation set. (**A**) ROC curves. (**B**) Decision curves ML, machine learning; ROC, receiver operating characteristic; KNN, K-Nearest Neighbor; LGBM, Light Gradient Boosting Machine; LR, Logistic Regression; RF, Random Forest; SVM, Support Vector Machine; XGB, eXtreme Gradient Boosting

Regarding the prediction of the in-hospital mortality of ICU patients, the performance of ML models is presented in Table S22 and Fig. 2. And the performance of clinical scores (APS III, SAPS II, SIRS, SOFA) is presented in Table S28.

APS III exhibited a sensitivity of 0.958 and an AUC of 0.859 in the validation set, emerging as the top performer among clinical scores. Its distinct advantage lies in the remarkably low risk of misdiagnosis, rendering it highly suitable for initial screening purposes. Nevertheless, it demonstrated a specificity of 0.606 and a pos pred value (PPV) of only 0.274. This implies a false positive rate of 72.6%, which may readily lead to the inefficient utilization of resources.

SAPS II attained a sensitivity of 0.875 and an AUC of 0.839 in the validation set. Yet, its specificity was recorded at 0.665, and the PPV stood at 0.288. Similar to the APSIII, it has analogous advantages and limitations, and can solely serve as a rapid screening instrument.

SIRS attained an AUC of 0.660 and an F1 score of 0.297 in the validation set, presenting the poorest performance. Additionally, its specificity was merely 0.342, and the PPV was only 0.177. Despite its computational simplicity, it lacks practical predictive utility.

SOFA achieved an AUC of 0.814 in the validation set, indicating moderate performance. Its PPV was 0.284, and the F1 score was 0.418, while the specificity was 0.690. Although it enjoys high clinical prevalence, its overall performance is comprehensively eclipsed by the ML model.

Among these clinical scores, APS III and SAPS II are appropriate for initial screening. Their elevated sensitivity effectively mitigates the risk of missed diagnoses. However, due to their relatively high false positive rates, secondary verification is imperative. In comparison to these clinical scores, the ML models in this research outshine them across multiple metrics, particularly the SVM and XGB models. Whether in terms of generalization ability (AUC > 0.900), specificity (> 0.900), or predictive precision (PPV > 0.580), these models demonstrate a significant edge over clinical scores.

#### External validation of ML models

**Fig. 3:**
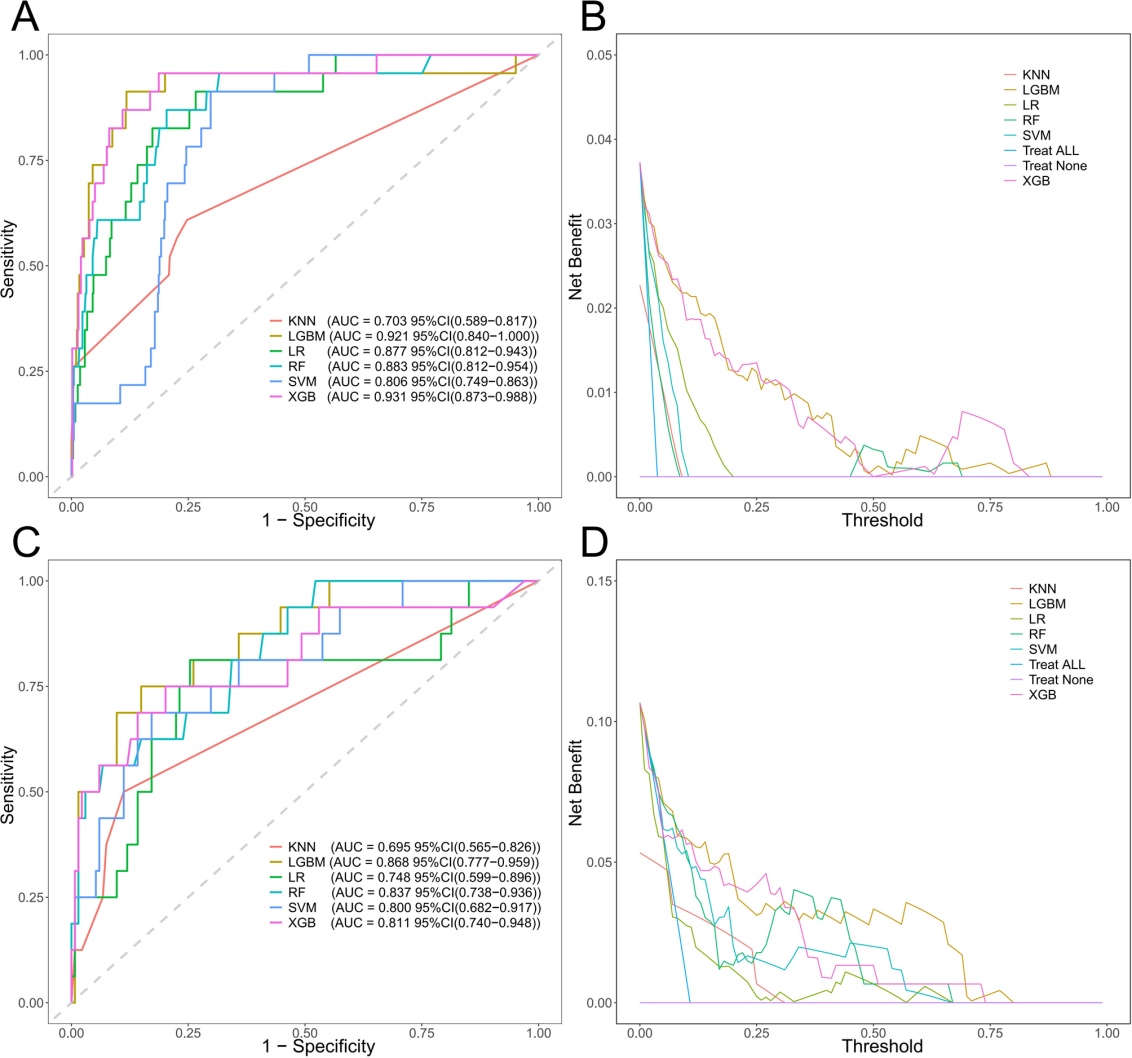
ROC curves and decision curves of the ML models in the external validation sets. (**A**-**B**) eICU database. (**C**-**D**) Wenzhou Hospital ML, machine learning; ROC, receiver operating characteristic; KNN, K-Nearest Neighbor; LGBM, Light Gradient Boosting Machine; LR, Logistic Regression; RF, Random Forest; SVM, Support Vector Machine; XGB, eXtreme Gradient Boosting

To further verify the reliability of these ML models for predicting in-hospital mortality in this patient set, additional validations were conducted with two external validation sets (Table S29). The effectiveness of the ML models in forecasting results is detailed in Table S30 and Fig. 3.

#### Selection of ML models

The KNN model is capable of rapidly fitting simple patterns, attributed to the adaptability of its non-parametric characteristics to local non-linear relationships. In the training set, the AUC of this model reaches 1.000. However, in the external validation sets, the KNN model reveals significant limitations. Specifically, the AUC in the eICU and Wenzhou datasets decreases to 0.703 and 0.695 respectively, with the sensitivity fluctuating within the range of 0.250 to 0.478. This finding implies that the KNN model is highly sensitive to the distance calculations of high-dimensional features. As such, it confronts difficulties in overcoming the curse of dimensionality and lacks a generalization mechanism for capturing non-linear relationships. Furthermore, the specificity decreases from 1.000 in the training set to 0.791 in the eICU dataset. This decline indicates that the model’s excessive reliance on local similarity leads to a severe deficiency in model stability. Consequently, the KNN model is entirely unsuitable for the analysis of high-dimensional data.

The LGBM model manifests remarkable advantages in processing high-dimensional data by virtue of the gradient boosting framework. The automatic feature selection mechanism of the LGBM model attained outstanding AUCs of 0.921 and 0.868 in the eICU and Wenzhou datasets respectively. This achievement validates the model’s proficiency in filtering redundant features. The tree-structured design of the model effectively captures non-linear relationships. Notably, the reduction in AUC from the training set to the validation set is smaller compared to that of most models, thereby evidencing its robust anti-overfitting capacity. In the external validation sets, the F1 score of the LGBM model fluctuated within the range of 0.456–0.474, which stands as the optimal value among all models, further substantiating its balanced predictive performance. Nevertheless, it was observed that in the Wenzhou dataset, the sensitivity of the LGBM model decreased to 0.562. Evidently, its stability is inferior to that of the XGB model. This observation implies that the LGBM model may have limitations in identifying minority classes (such as death cases), potentially impeding the screening of high-risk patients in clinical settings.

The parameter sparsity of the LR model mitigates the risk of overfitting and showcases commendable stability within high-dimensional settings. In the eICU and Wenzhou datasets, this model achieved remarkable AUC values of 0.877 and 0.748, respectively. Correspondingly, the sensitivities were measured at 0.696 and 0.500. While the transparency of its linear structure offers pivotal interpretability for clinical decision-making, the linear assumption unfortunately emerges as a critical shortcoming. In the eICU dataset, the PPV was found to be a mere 0.163, indicating that the model falls short in discerning the intricate interactions among features. Such a high false-positive rate indicates that when the incidence of disease is relatively low, the use of this model for clinical screening will escalate the screening workload. Consequently, the practical predictive utility of this model remains circumscribed.

The RF model fortifies its non-linear modeling capacity through the integration of multiple decision trees. In the training set and the internal validation set, this model attained AUC values of 1.000 and 0.938 respectively. Nonetheless, in the external validation sets, certain deficiencies of the RF model became apparent. Specifically, in the eICU and Wenzhou datasets, the sensitivity decreased to 0.304 and 0.438 respectively, whereas the specificity soared to over 0.976, presenting a rather inflated value. This pronounced imbalance implies that despite the fact that the random feature subset strategy can relieve some of the dimensionality-related challenges, the generalization ability of the tree-based structure of the RF model for intricate non-linear patterns remains restricted. Additionally, it struggles to adapt to the variances in data distribution across different centers. Consequently, when the distribution of features undergoes changes, the rate of missed diagnoses of this model experiences a substantial upsurge, ultimately resulting in the forfeiture of its clinical practical significance.

The SVM model employs the kernel trick to establish classification boundaries in high-dimensional spaces. In the internal validation set, it has demonstrated excellent performance, achieving an AUC of 0.934, a specificity of 0.910, and a sensitivity of 0.833. However, during the external validation process, certain deficiencies of the SVM model have come to light. Specifically, in the eICU and Wenzhou datasets, the AUC values have decreased to 0.806 and 0.800 respectively. This phenomenon indicates that the performance of the SVM model is highly contingent upon parameter optimization. Furthermore, within the eICU dataset, the F1 score is 0.169, and the PPV is 0.099. These figures confirm that the SVM model lacks reliability in predicting positive cases, thereby circumscribing its value in clinical decision-making. At the core of the problem is the fact that the linear kernel function has significant limitations in modeling complex non-linear interactions within high-dimensional data. Additionally, it is highly sensitive to class imbalance issues. Compounding these problems is the fact that the computational complexity of the SVM model grows exponentially with the increase in dimensionality. As a consequence, the SVM model is the least viable for practical applications in the clinical domain.

The XGB model combines regularization strategies with gradient boosting mechanisms, setting a benchmark for processing high-dimensional non-linear data. In the present study, it has demonstrated the most well-balanced comprehensive performance. In the eICU and Wenzhou datasets, this model attained optimal AUC of 0.931 and 0.811, respectively. The regularization mechanism and tree-pruning strategy employed by the XGB model significantly fortify its resilience against high-dimensional overfitting, thereby minimizing the reduction in AUC from the training set to the validation set. The tree-ensemble architecture of the XGB model automatically sifts out crucial features. This enables the model to strike an ideal balance between sensitivity and specificity. In the eICU dataset, when the specificity reached 0.960, the model managed to uphold a sensitivity of 0.609. Moreover, the neg pred value(NPV) soared to 0.984. Addressing the prevalent issue of sample imbalance in data, both the F1 score (0.459) and PPV (0.368) of the XGB model significantly outperformed those of comparable models. This accomplishment realizes a relatively harmonious equilibrium between missed diagnoses and misdiagnoses.

The decision curve analysis curve further attests to the superiority of the XGB model. As can be seen from the figure, the XGB model exhibits relatively stable performance in both the internal and external validation sets, decidedly outperforming other models. In the core threshold interval (0.2–0.4), its curve shows a gentle decline, which can adapt to diverse requirements for medical resource allocation. This indicates that the XGB model is capable of effectively balancing the identification of high-risk patients and the costs associated with false positives. Conversely, in the external validation set, other ML models manifest a precipitous drop in performance, which reflects their inadequate generalization capabilities and thus limited clinical practicality. Notably, when the threshold surpasses 0.4, the net benefit of the XGB model attenuates significantly. Therefore, it is necessary to adjust the decision-making threshold in combination with clinical risk preferences.

In summary, among the ML models for predicting in-hospital mortality of ICU patients, the XGB model exhibits multiple advantages, thereby qualifying as the optimal model. These advantages include cross-center AUC stability (with fluctuations < 0.2), a balance between sensitivity and specificity (both exceeding 0.850), and a regularization-based mechanism to counter overfitting. Such characteristics are highly congruent with the core requirement of robustness in medical scenarios. While the LR model offers interpretability, it incurs a relatively high cost of false positives. The LGBM model, despite having performance comparable to that of the XGB model, demonstrates slightly inferior stability in the face of abrupt changes in data distribution. The RF, KNN, and SVM models have been excluded owing to their unacceptable generalization limitations.

#### Model interpretation by the SHAP method

**Fig. 4:**
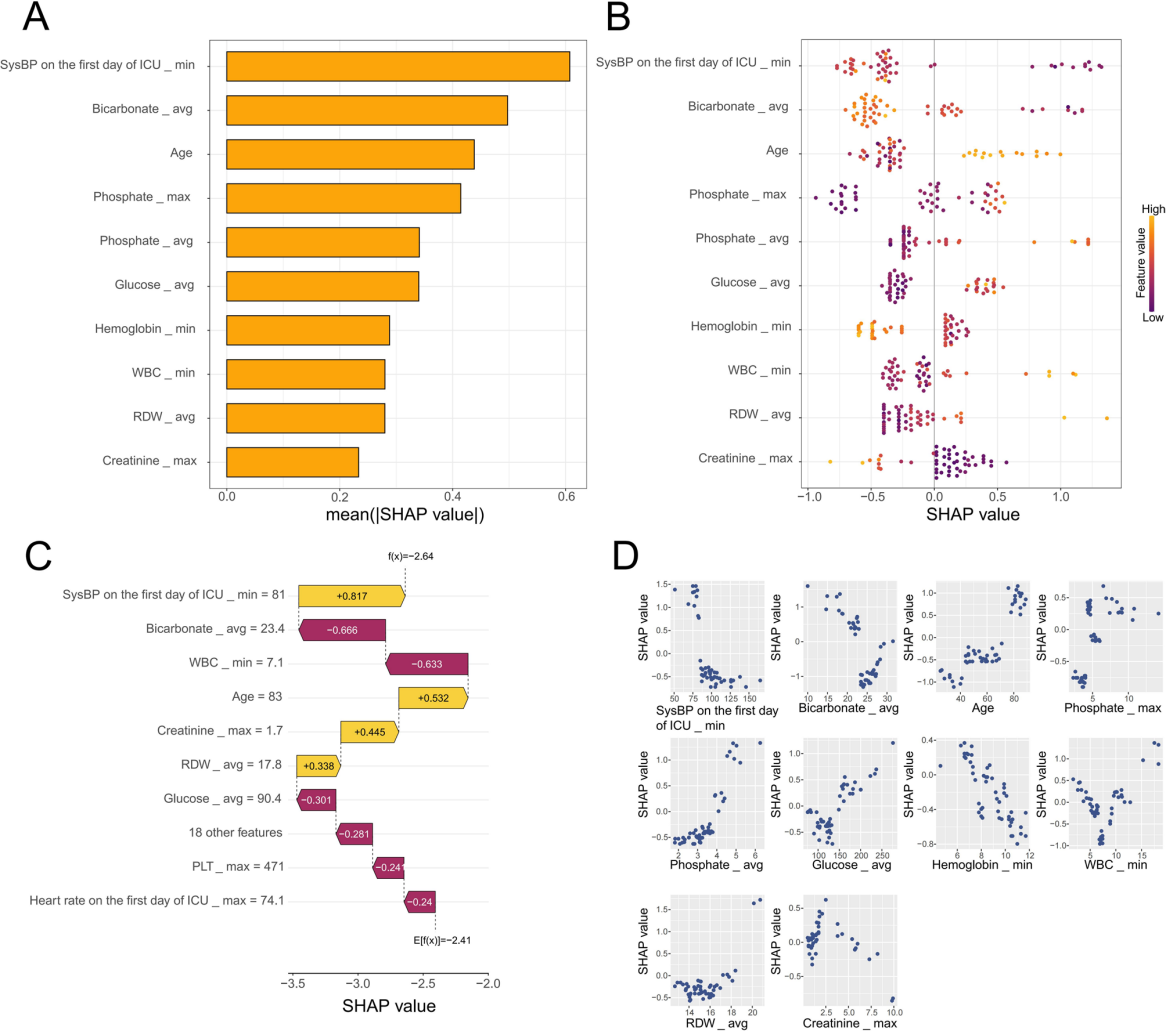
Global and local model explanation via the SHAP method. (**A**) SHAP summary bar plot assesses the contribution of each feature to the model through the utilization of mean SHAP values, listing the ten most significant features in order of importance, presented in descending sequence. (**B**) SHAP summary dot plot shows that the probability of in-hospital mortality ascends with the SHAP values of the features. Each dot represents a patient’s SHAP value for a given feature, with orange indicating higher feature values and purple signifying lower values. Dots are vertically stacked to exhibit density. (**C**) SHAP force plot showcases the contribution of each feature to the prediction result of the third patient using the Random Forest model. Yellow bars signify features that contribute positively to the prediction, while purple bars denote negative contributions. Feature values are displayed in conjunction with their SHAP values, highlighting key features. (**D**) SHAP dependence plot reveals how a single feature affects the output of the model, with each point representing a patient. SHAP, shapley additive explanations

We utilize the SHAP method to clarify the outputs of XGB model by assessing how much each variable contributes to its predictions. This interpretable approach provides two forms of explanations: a global overview at the feature level and a local perspective for individual cases (Fig. 4).

In the SHAP summary bar plot, we use SHAP averages to measure feature contributions to the model (Fig. 4A).

The SHAP summary dot plot visually illustrates both the direction and magnitude of these features’ effects on predictions made by the model (Fig. 4B). These plots list the top 10 most significant features in order of importance and visually elucidates the direction and magnitude of their influence on model predictions. It can be observed from the figure that among the conventional indicators, SysBP on the first day of ICU_min, bicarbonate_avg, and age are the top three potent predictive features.

The SHAP force plot shows how these features contribute to prediction outcomes for one specific patient using the XGB model (Fig. 4C). By visualizing the SHAP values of the samples, we can systematically assess the influence of each feature on the model predictions for these specific instances. For the surviving patient, the predicted probability of in-hospital mortality was relatively low as SysBP on the first day of ICU_min = 81 mmHg, Bicarbonate_avg = 23.4 mEq/L, Age = 83.

Furthermore, the SHAP dependence plot aids in understanding how particular features influence the output generated by our prediction model (Fig. 4D).

## Discussion

This study evaluated the predictive performance of ML models for clinical outcomes in AP patients during hospitalization and after discharge. The XGB model showed superiority in predicting in-hospital mortality, long-term mortality (180/365 days), and supporting clinical decisions. To our knowledge, this is the first multicenter retrospective study on short- and long-term AP prognosis.

### Model interpretability and variable selection

In our study, for different populations corresponding to distinct outcomes, each model encompassed 11–27 clinical parameters. Concerning the quantity of variables in the prediction model, currently, due to the absence of criteria or consensus for variable selection in prediction models, the number of variables that should be incorporated in the prediction model remains undetermined. Although large number of variables can provide more information to the prediction model, it may limit the model’s application in clinical settings. Moreover, when there are many variables, correlations often occur among them, causing multicollinearity and several complications in the model.

In this study, prior to constructing the model, we first employed LASSO regression for variable selection. LASSO regression helps to develop a more streamlined model by selecting only the most important predictors, which in turn improves both the interpretability and generalizability of the model.

ML techniques are frequently characterized as “black boxes” since it is arduous to explain how predictions are derived through conventional operations. Presently, SHAP analysis has been extensively applied in the medical domain and is utilized as an interpretative approach for ML models [22–25]. We adopted the SHAP method to aid in variable selection and further employed it to explain the variable selection process of the ML model. The SHAP method is designed to assess the significance of input features and elucidate the outcomes of prediction models. Grounded in Shapley value theory, it offers both global and local interpretability by breaking down the prediction results into the contributions made by each feature.

### The clinical significance of key variables

In this study, when establishing the ML model for predicting the in-hospital mortality of ICU-AP patients, a total of 27 closely related predictors were screened (Supplementary Table S9). These variables not only exhibit statistical significance but also concordant with the clinical significance of AP.

We employed the SHAP method to evaluate the contribution of each variable to the predictions of the XGB model. Among them, the top 10 variables are as follows: SysBP on the first day of ICU_min, Bicarbonate_avg, Age, Phosphate_max, Phosphate_avg, Glucose_avg, Hemoglobin_min, WBC_min, RDW_avg, and Creatinine_max.

Low systolic blood pressure serves as a prominent indicator of systemic inflammatory response syndrome and shock. On one hand, in cases of SAP, the massive release of inflammatory mediators gives rise to vasodilation, capillary leakage, a precipitous decline in the effective circulating blood volume, and myocardial depression. This directly culminates in inadequate tissue perfusion, thereby triggering or exacerbating multiple organ dysfunction syndrome. On the other hand, hypotension per se reflects the exhaustion of the body’s compensatory mechanisms. The lower the minimum systolic blood pressure, the more intense the inflammatory storm, the more profound the hemodynamic decompensation, and the higher the risk of tissue hypoperfusion and organ damage [26]. When the systolic blood pressure persists below 90 mmHg within the first day in the ICU, this may foreshadow the development of septic shock, and immediate initiation of fluid resuscitation is warranted.

Bicarbonate is the primary buffering base in the human body. The decline in its level can be primarily attributed to several factors. These include the accumulation of lactic acid resulting from tissue hypoperfusion, the impaired excretion of acidic metabolic products caused by acute kidney injury, and the release of acidic substances from necrotic pancreatic tissue. In cases of SAP, it is frequently observed that bicarbonate levels remain persistently low or exhibit a progressive decline. This phenomenon is a comprehensive manifestation of sustained tissue hypoperfusion, severe inflammatory responses, and organ dysfunction. It reflects the extent and duration of the disruption of the body’s acid-base equilibrium and serves as an indicator of a critically ill condition that is continuously deteriorating [27].

Age does not directly mirror the current inflammatory extent of acute pancreatitis. Nevertheless, when the severity of the disease is equivalent, the mortality risk among elderly patients is notably higher compared to that of younger patients [28]. This can be ascribed to the fact that elderly patients generally exhibit a decline in physiological reserve function, are afflicted with multiple chronic diseases, possess weakened immune function, have a reduced capacity for tissue repair, experience alterations in drug metabolism, and may suffer from potential malnutrition. Consequently, when confronted with acute and severe insults, elderly patients are more susceptible to developing severe organ dysfunction. Moreover, their tolerance and responsiveness to treatment are relatively poor.

An elevation in phosphate levels serves as a potent indication of extensive cellular necrosis and severe impairment of renal function. This phenomenon reflects both the severity of tissue destruction and the exhaustion of the body’s capacity to eliminate metabolic waste products. In patients with AP, two main factors contribute to this situation. Firstly, pancreatic necrosis leads to the destruction of a large number of tissue cells, thereby releasing intracellular phosphate. Secondly, acute kidney injury reduces the excretion of phosphate. Furthermore, severe hyperphosphatemia can give rise to electrolyte disorders, soft tissue calcification, and exacerbate cardiovascular instability. Clinically, hyperphosphatemia has been associated with an increased mortality rate among critically ill patients. As such, it serves as an important indicator of disease severity and organ dysfunction [29].

Persistent hyperglycemia is frequently observed in SAP and is attributed to “stress-induced hyperglycemia”. During the early stage of AP development, a substantial release of inflammatory mediators and stress hormones occurs. This leads to insulin resistance, augmented hepatic gluconeogenesis, accelerated glycogenolysis, and relatively insufficient insulin secretion. A hyperglycemic milieu can compromise immune function, promote oxidative stress and endothelial injury, elevate the risk of infection, and may directly exacerbate pancreatic damage. A consistently elevated average blood glucose level reflects the body’s continuous exposure to a severe stress and inflammatory state. Difficulty in blood glucose control often foreshadows an uncontrolled inflammatory response, indicating a severe and complex disease condition. In line with the findings of previous studies, this research confirms that Glucose_avg is closely associated with the in-hospital mortality of AP patients [30].

The lowest hemoglobin value serves as an indicator of the severity of anemia. Anemia is prevalent among critically ill patients. In the context of AP, several factors can contribute to low hemoglobin levels: Inflammatory anemia, for instance, can occur when inflammatory mediators inhibit bone marrow hematopoiesis, shorten the lifespan of erythrocytes, and disrupt iron metabolism. Hemodilution, often resulting from aggressive fluid resuscitation, is another contributing factor. Additionally, underlying nutritional deficiencies may also play a role. Anemia reduces the oxygen-carrying capacity of the blood. During periods of increased tissue oxygen demand, such as in a stress response, this can exacerbate organ hypoxia. A lower lowest hemoglobin value in patients indicates more substantial blood loss, more severe suppression of hematopoiesis by inflammation, or potential issues with the fluid resuscitation strategy.

The lowest WBC count is an unconventional yet potentially pivotal biomarker. In an inflammatory milieu where an elevation in WBCs is typically anticipated, a low value often implies bone marrow suppression, excessive depletion of immune cells in peripheral tissues, or even a state of immune system exhaustion. Within the context of AP, a sustained decline in WBC counts is particularly worthy of vigilant attention. This situation is highly likely to be associated with disease exacerbation or the emergence of complications.

An elevated RDW indicates an augmented heterogeneity in the size of red blood cells. In the context of chronic and critical diseases, an increased RDW has been linked to chronic inflammation, malnutrition, bone marrow stress, oxidative stress, and endothelial dysfunction. Inflammatory mediators disrupt iron metabolism and erythropoiesis, resulting in the generation of red blood cells with a wide range of sizes. In the case of AP, persistent inflammation and potential nutritional deficiencies are the primary contributing factors. RDW serves as a comprehensive indicator that reflects the cumulative effects of a patient’s chronic health burden and superimposed acute inflammation. Among ICU patients, a high RDW has been extensively demonstrated to be an independent predictor of mortality across multiple disease entities. This suggests that patients with a high RDW have poor physiological reserves, low tolerance to acute stressors, and limited capacity for recovery [31].

Serum creatinine is one of the most critical markers for reflecting the decline in glomerular filtration rate, thereby indicating the severity of AKI. A higher maximum creatinine value suggests a potentially more severe renal injury. In cases of SAP, the mechanisms underlying AKI are intricate. They encompass prerenal factors (such as hypovolemic shock and reduced cardiac output), renal factors (including the direct nephrotoxic effects of inflammatory mediators and the formation of microthrombi), and postrenal factors (for example, the compression of the kidneys or ureters due to abdominal compartment syndrome). AKI is a robust predictor of poor prognosis in AP patients, significantly elevating the risk of mortality [31]. Creatinine_max not only reflects the degree of injury to the kidneys themselves but also serves as an important indicator of systemic hemodynamic derangements, the intensity of the inflammatory storm, and the overall organ dysfunction.

### Comparison of the models with clinical scores

There exists many clinical scores employed for predicting poor prognoses in patients, including the APS III, SAPS II, SOFA, and SIRS.

Both the APS III and SAPS II serve to evaluate the severity of illness and the mortality risk of ICU patients. The APS III constitutes the physiological scoring component of APACHE III [32]. It demonstrates high accuracy in predicting mortality and is applicable to diverse groups of ICU patients. This score necessitates 17 physiological indicators, incorporating the most aberrant physiological state within 24 h post-admission. In this study, it exhibited the optimal AUC among all clinical scores.

The calculation of the SAPS II is founded on 12 physiological parameters, age, hospitalization type, and chronic diseases [33]. It is noteworthy that the data it collects pertains to the first 24 h upon ICU admission and does not encompass subsequent dynamic alterations. Nevertheless, for specific patient populations, the prediction bias of the SAPS II may be substantial. In this study, its AUC performance ranked second only to the APS III.

The SOFA and SIRS have, in prior research, predominantly focused on predicting the in-hospital mortality of patients suspected of having an infection. The SIRS is computed based on four parameters: body temperature, heart rate, respiratory rate, and white blood cell count, aiming to identify inflammatory responses [34]. While it can promptly indicate potential infection or inflammatory states and is convenient to use, it suffers from low specificity. Moreover, non-infectious factors such as trauma and surgery can also trigger it. Consequently, it has gradually been supplanted by the SOFA. In this study, its predictive capacity was subpar.

The SOFA assesses the degree of dysfunction in six major organ systems to dynamically monitor the progression of the disease [35]. Notably, it requires repeated measurement of multiple parameters, and some items are susceptible to the influence of therapeutic interventions. In this study, its predictive performance regarding the in-hospital mortality of ICU patients was commendable.

In comparison to these clinical scores, the model developed in this study demonstrated a significantly superior predictive ability regarding the in-hospital mortality of ICU patients. This finding further validates the superiority of its predictive performance. The clinical variables incorporated into this model are multi-dimensional, encompassing demographic characteristics, laboratory indicators, and real-time vital signs. Importantly, the model does not merely rely on data collected within the initial 24 h following ICU admission; it also integrates continuous dynamic measurement data obtained thereafter. This approach enables the model to function throughout the entirety of the patient’s hospital stay and offer ongoing tracking predictions as the patient’s condition progresses.

### The relationship between readmission and post-discharge mortality

This study analyzed the relationship between readmission and post-discharge mortality in AP patients, leveraging data from the MIMIC-IV database and external validation sets. Our results demonstrated no significant correlation between readmission within 30/60/90 days after discharge and mortality within 180/365 days after discharge (Table 3) for all AP patients. For those patients who underwent surgical intervention and ICU admission, the short-term readmission rate decreased, but paradoxically, the post-discharge mortality rate increased (Table 4, Tables S4-5). This finding is consistent with clinical experience. As patients with severe conditions requiring surgical intervention/ICU treatment typically have a higher risk of death. However, adequate surgical intervention/ICU treatment enhances the possibility of cure, thereby reducing the likelihood of readmission for these patients. As indicated by the key variables screened (Table S9), readmission was mainly associated with transient factors such as electrolyte and metabolic disorders (Chloride_min, Glucose_max/avg) or hematological indicators (PLT_min, RDW_avg), while post-discharge mortality was mainly related to organ dysfunction factors such as Urea nitrogen_max, Creatinine_max, and SOFA. These paradoxical trends suggest that readmission and mortality in AP patients are driven by distinct clinical pathways, necessitating a reevaluation of their roles as composite endpoints in prognostic studies.

In this study, all ML models performed poorly in predicting readmission rates. Even though the XGB model achieved a robust AUC (> 0.800) in predicting in-hospital mortality, its performance in predicting readmission rates remained suboptimal (AUC < 0.700). This might be attributed to the fact that the outcome of readmission is influenced by various unstructured variables, such as social factors (e.g., home care, economic status) and follow-up compliance. Additionally, early readmission after discharge may be related to the residual effects of acute-phase treatment rather than long-term pathological processes. The existing databases have difficulty in fully collecting such information. In conclusion, the readmission rate of AP patients may not be suitable as a follow-up indicator for long-term quality of life.

### Clinical significance of models

The utilization of the XGB model in clinical practice can prominently optimize the allocation of resources. On the one hand, efficiently identifying low-risk patients (NPV > 0.923) can prevent the excessive utilization of medical resources, such as repetitive abdominal CT scans within a short period. On the other hand, early intervention for high-risk patients can lower the incidence of complications and thus reduce the average length of hospital stay. Furthermore, this intelligent risk stratification system can also decrease the time spent by medical staff and thereby increase work efficiency.

### Strengths and limitations

This study presents several strengths. Firstly, this study is the first retrospective investigation of both the short-term and long-term prognoses of AP patients. We have elaborately analyzed and compared the predictive performance of the ML model in different outcomes. Secondly, we employed multi-center data. The data originated from multiple regions and various ethnic groups. The ML models demonstrated excellent performance in the internal validation set as well as in two external validation sets, showcasing favorable predictive capabilities. This indicates that the model is not restricted by geographical locations and ethnicities and possesses robustness and practicality. Additionally, there was a possibility of multiple positive outcomes for some patients. For instance, patients who were readmitted to the hospital within 30 days after discharge and then died within 365 days after discharge. This is an actual phenomenon in clinical practice, but it has often been overlooked in previous studies. In this study, we further analyzed the correlation between multiple positive results. Thirdly, the acute pancreatitis prediction model developed in this study demonstrated significantly better performance in predicting the in-hospital mortality of ICU patients compared to clinical scores, including APS III, SAPS II, SIRS, SOFA. Finally, the clinical variables utilized for constructing the model were multi-dimensional. The clinical variables collected in this study encompassed dynamic measurements during hospitalization. This allowed the model to operate continuously during the treatment period and provide ongoing tracking predictions as the disease progressed while the patient was hospitalized.

Nonetheless, this study has several limitations. Firstly, our model was developed retrospectively using data from a single database, which unavoidably led to both selection bias and detection bias in the process of data collection. Secondly, since this study utilized data from multiple centers, it could only investigate the variables that were common to all centers. Finally, this model can only predict the occurrence of prognostic outcomes but is unable to predict the timing of these outcomes. Further research is required to investigate the prediction of the timing of outcome occurrence.

## Conclusions

Our study demonstrates that the XGB model is beneficial in identifying the short-term and long-term prognosis of AP patients, especially in predicting the in-hospital mortality of AP patients admitted to the ICU. These capabilities directly support clinical decision-making for healthcare providers.

## Electronic supplementary material

Below is the link to the electronic supplementary material.

Supplementary Material 1

## Data Availability

Some of the original data presented in the study are publicly available. The data can be found in the eICU-CRD database: https://eicu-crd.mit.edu/gettingstarted/access/ Additionally, the data can be found in the MIMIC-IV database: https://physionet.org/content/mimiciv/3.0/ The other original data presented in the study are available from the authors but restrictions apply to the availability of these data, which were used under license from the Ethics Committee at the First Affiliated Hospital of Wenzhou Medical University for the current study, and so are not publicly available. Data are, however, available from the authors upon reasonable request.

## Abbreviation

AP: Acute pancreatitis
APACHE: Acute physiology and chronic health Evaluation
APS III: Acute Physiology Score III
AUC: Area under the curve
ICU: Intensive care unit
KNN: K-Nearest Neighbor
LASSO: Least Absolute Shrinkage and Selection Operator
LGBM: Light Gradient Boosting Machine
LR: Logistic Regression
ML: Machine learning
NPV: Neg pred value
PPV: Pos pred value
RF: Random Forest
ROC: Receiver operating characteristic
SAP: Severe acute pancreatitis
SAPS II: Simplified Acute Physiology Score II
SHAP: SHapley Additive exPlanations
SIRS: Systemic Inflammatory Response Syndrome Criteria
SOFA: Sequential Organ Failure Assessment
SVM: Support Vector Machine
XGB: eXtreme Gradient Boosting
