# Supplementary material for: Utilizing machine learning models for predicting outcomes in acute pancreatitis: development and validation in three retrospective cohorts
Source: BMC Med Inform Decis Mak. 2025 Jul 11;25:261. doi: 10.1186/s12911-025-03103-7 (PMC12247377; doi:10.1186/s12911-025-03103-7)
Supplement: Supplementary file 1 — Supplementary Material 1 [file 12911_2025_3103_MOESM1_ESM.docx]

**Table S1. Variables classified according to the type of data distribution corresponding to various patient categories**

| All the patients. (n=86) | | | Patients underwent operations. (n=85) | | | Patients admitted to the ICU. (n=112) | | |
| --- | --- | --- | --- | --- | --- | --- | --- | --- |
| Normal distribution variables | Abnormal distribution variables | Categorical variables | Normal distribution variables | Abnormal distribution variables | Categorical variables | Normal distribution variables | Abnormal distribution variables | Categorical variables |
|  | Age | Gender |  | Age | Gender |  | Age | Gender |
|  | Length of stay in hospital | Surgical operations |  | Length of stay in hospital | Surgical operations |  | Length of stay in hospital | Surgical operations |
|  | Length of stay in ICU | Endoscopy |  | Length of stay in ICU | Endoscopy |  | Length of stay in ICU | Endoscopy |
|  | Anion gap _ max | Interventional operations |  | Anion gap _ max | Interventional operations |  | Anion gap _ max | Interventional operations |
|  | Anion gap _ avg | Surgical or interventional or endoscopic operations |  | Anion gap _ avg | Endoscopy combined with surgical operations |  | Anion gap _ avg | Endoscopy combined with surgical operations |
|  | Anion gap _ min | Endoscopy combined with surgical operations |  | Anion gap _ min | Endoscopy combined with interventional operations |  | Anion gap _ min | Endoscopy combined with interventional operations |
|  | Bicarbonate _ max | Endoscopy combined with interventional operations |  | Bicarbonate _ max | Surgical or interventional operations other than endoscopy |  | Bicarbonate _ max | Surgical or interventional or endoscopic operations |
|  | Bicarbonate _ avg | Surgical or interventional operations other than endoscopy |  | Bicarbonate _ avg | Endoscopic, surgical, and interventional procedures were performed during a single hospitalization |  | Bicarbonate _ avg | Surgical or interventional operations other than endoscopy |
|  | Bicarbonate _ min | Endoscopic, surgical, and interventional procedures were performed during a single hospitalization |  | Bicarbonate _ min | Hypertension |  | Bicarbonate _ min | Endoscopic, surgical, and interventional procedures were performed during a single hospitalization |
|  | Chloride _ max | Hypertension |  | Chloride _ max | Diabetes |  | Chloride _ max | Hypertension |
|  | Chloride _ avg | Diabetes |  | Chloride _ avg |  |  | Chloride _ avg | Diabetes |
|  | Chloride _ min |  |  | Chloride _ min |  |  | Chloride _ min | Sepsis |
|  | Creatinine _ max |  |  | Creatinine _ max |  |  | Creatinine _ max | AKI |
|  | Creatinine _ avg |  |  | Creatinine _ avg |  |  | Creatinine _ avg |  |
|  | Creatinine _ min |  |  | Creatinine _ min |  |  | Creatinine _ min |  |
|  | Glucose _ max |  |  | Glucose _ max |  |  | Glucose _ max |  |
|  | Glucose _ avg |  |  | Glucose _ avg |  |  | Glucose _ avg |  |
|  | Glucose _ min |  |  | Glucose _ min |  |  | Glucose _ min |  |
|  | Potassium _ max |  |  | Potassium _ max |  |  | Potassium _ max |  |
|  | Potassium _ avg |  |  | Potassium _ avg |  |  | Potassium _ avg |  |
|  | Potassium _ min |  |  | Potassium _ min |  |  | Potassium _ min |  |
|  | Sodium _ max |  |  | Sodium _ max |  |  | Sodium _ max |  |
|  | Sodium _ avg |  |  | Sodium _ avg |  |  | Sodium _ avg |  |
|  | Sodium _ min |  |  | Sodium _ min |  |  | Sodium _ min |  |
|  | Urea nitrogen _ max |  |  | Urea nitrogen _ max |  |  | Urea nitrogen _ max |  |
|  | Urea nitrogen _ avg |  |  | Urea nitrogen _ avg |  |  | Urea nitrogen _ avg |  |
|  | Urea nitrogen _ min |  |  | Urea nitrogen _ min |  |  | Urea nitrogen _ min |  |
|  | Hematocrit _ max |  |  | Hematocrit _ max |  |  | Hematocrit _ max |  |
|  | Hematocrit _ avg |  |  | Hematocrit _ avg |  |  | Hematocrit _ avg |  |
|  | Hematocrit _ min |  |  | Hematocrit _ min |  |  | Hematocrit _ min |  |
|  | Hemoglobin _ max |  |  | Hemoglobin _ max |  |  | Hemoglobin _ max |  |
|  | Hemoglobin _ avg |  |  | Hemoglobin _ avg |  |  | Hemoglobin _ avg |  |
|  | Hemoglobin _ min |  |  | Hemoglobin _ min |  |  | Hemoglobin _ min |  |
|  | MCH _ max |  |  | MCH _ max |  |  | MCH _ max |  |
|  | MCH _ avg |  |  | MCH _ avg |  |  | MCH _ avg |  |
|  | MCH _ min |  |  | MCH _ min |  |  | MCH _ min |  |
|  | MCHC _ max |  |  | MCHC _ max |  |  | MCHC _ max |  |
|  | MCHC _ avg |  |  | MCHC _ avg |  |  | MCHC _ avg |  |
|  | MCHC _ min |  |  | MCHC _ min |  |  | MCHC _ min |  |
|  | MCV _ max |  |  | MCV _ max |  |  | MCV _ max |  |
|  | MCV _ avg |  |  | MCV _ avg |  |  | MCV _ avg |  |
|  | MCV _ min |  |  | MCV _ min |  |  | MCV _ min |  |
|  | PLT _ max |  |  | PLT _ max |  |  | PLT _ max |  |
|  | PLT _ avg |  |  | PLT _ avg |  |  | PLT _ avg |  |
|  | PLT _ min |  |  | PLT _ min |  |  | PLT _ min |  |
|  | RDW _ max |  |  | RDW _ max |  |  | RDW _ max |  |
|  | RDW _ avg |  |  | RDW _ avg |  |  | RDW _ avg |  |
|  | RDW _ min |  |  | RDW _ min |  |  | RDW _ min |  |
|  | RBC _ max |  |  | RBC _ max |  |  | RBC _ max |  |
|  | RBC _ avg |  |  | RBC _ avg |  |  | RBC _ avg |  |
|  | RBC _ min |  |  | RBC _ min |  |  | RBC _ min |  |
|  | WBC _ max |  |  | WBC _ max |  |  | WBC _ max |  |
|  | WBC _ avg |  |  | WBC _ avg |  |  | WBC _ avg |  |
|  | WBC _ min |  |  | WBC _ min |  |  | WBC _ min |  |
|  | TBil _ max |  |  | TBil _ max |  |  | TBil _ max |  |
|  | TBil _ avg |  |  | TBil _ avg |  |  | TBil _ avg |  |
|  | TBil _ min |  |  | TBil _ min |  |  | TBil _ min |  |
|  | ALT _max |  |  | ALT _max |  |  | ALT _max |  |
|  | ALT _avg |  |  | ALT _avg |  |  | ALT _avg |  |
|  | ALT _ min |  |  | ALT _ min |  |  | ALT _ min |  |
|  | ALP _ max |  |  | ALP _ max |  |  | ALP _ max |  |
|  | ALP _ avg |  |  | ALP _ avg |  |  | ALP _ avg |  |
|  | ALP _ min |  |  | ALP _ min |  |  | ALP _ min |  |
|  | AST _max |  |  | AST _max |  |  | AST _max |  |
|  | AST _avg |  |  | AST _avg |  |  | AST _avg |  |
|  | AST _min |  |  | AST _min |  |  | AST _min |  |
|  | Magnesium _ max |  |  | Magnesium _ max |  |  | Magnesium _ max |  |
|  | Magnesium _ avg |  |  | Magnesium _ avg |  |  | Magnesium _ avg |  |
|  | Magnesium _ min |  |  | Magnesium _ min |  |  | Magnesium _ min |  |
|  | Calcium _ max |  |  | Calcium _ max |  |  | Calcium _ max |  |
|  | Calcium _ avg |  |  | Calcium _ avg |  |  | Calcium _ avg |  |
|  | Calcium _ min |  |  | Calcium _ min |  |  | Calcium _ min |  |
|  | Phosphate _ max |  |  | Phosphate _ max |  |  | Phosphate _ max |  |
|  | Phosphate _ avg |  |  | Phosphate _ avg |  |  | Phosphate _ avg |  |
|  | Phosphate _ min |  |  | Phosphate _ min |  |  | Phosphate _ min |  |
|  |  |  |  |  |  |  | SIRS score |  |
|  |  |  |  |  |  |  | SOFA score |  |
|  |  |  |  |  |  |  | Duration of auxiliary ventilation |  |
|  |  |  |  |  |  |  | Heart rate on the first day of ICU _ max |  |
|  |  |  |  |  |  |  | Heart rate on the first day of ICU _ avg |  |
|  |  |  |  |  |  |  | Heart rate on the first day of ICU _ min |  |
|  |  |  |  |  |  |  | SysBP on the first day of ICU _ max |  |
|  |  |  |  |  |  |  | SysBP on the first day of ICU _ avg |  |
|  |  |  |  |  |  |  | SysBP on the first day of ICU _ min |  |
|  |  |  |  |  |  |  | DiasBP on the first day of ICU _ max |  |
|  |  |  |  |  |  |  | DiasBP on the first day of ICU _ avg |  |
|  |  |  |  |  |  |  | DiasBP on the first day of ICU _ min |  |
|  |  |  |  |  |  |  | Mean arterial pressure on the first day of ICU admission _ max |  |
|  |  |  |  |  |  |  | Mean arterial pressure on the first day of ICU admission _ avg |  |
|  |  |  |  |  |  |  | Mean arterial pressure on the first day of ICU admission _ min |  |
|  |  |  |  |  |  |  | Respiratory rate on the first day of ICU _ max |  |
|  |  |  |  |  |  |  | Respiratory rate on the first day of ICU _ avg |  |
|  |  |  |  |  |  |  | Respiratory rate on the first day of ICU _ min |  |
|  |  |  |  |  |  |  | Temperature on the first day of ICU admission _ max |  |
|  |  |  |  |  |  |  | Temperature on the first day of ICU admission _ avg |  |
|  |  |  |  |  |  |  | Temperature on the first day of ICU admission _ min |  |
|  |  |  |  |  |  |  | SpO2 on the first day of ICU _ max |  |
|  |  |  |  |  |  |  | SpO2 on the first day of ICU _ avg |  |
|  |  |  |  |  |  |  | SpO2 on the first day of ICU _ min |  |

ICU, intensive care unit; MCH, mean corpuscular hemoglobin; MCHC, mean corpuscular hemoglobin concentration; MCV, mean corpuscular volume; PLT, platelet count; RDW, red cell distribution width; RBC, red blood cells; WBC, white blood cells; TBil, total bilirubin; ALT, alanine aminotransferase; ALP, alkaline phosphatase; AST, aspartate aminotransferase; SIRS, systemic inflammatory response syndrome; SOFA, sepsis-related organ failure; AKI, acute kidney injury; SysBP, systolic blood pressure; DiasBP, diastolic blood pressure; SpO2, saturation of peripheral oxygen.

**Table S2. Summary table of conversion relationship between variable data units collected from Wenzhou Hospital, eICU database and existing data units of MIMIC database**

| Variables | MIMIC database | eICU database | Wenzhou hospital | Conversion formulas |
| --- | --- | --- | --- | --- |
| Age | years | years | years | - |
| Bicarbonate | mEq/L | mmol/L | mmol/L | 1 mEq/L = 1 mmol/L |
| Creatinine | mg/dL | mg/dL | umol/L | 1 mg/dL= 88.4 umol/L |
| Glucose | mg/dL | mg/dL | mmol/L | 1 mmol/L = 18 mg/dL |
| Potassium | mEq/L | mmol/L | mmol/L | 1 mEq/L = 1 mmol/L |
| Urea nitrogen | mg/dL | mg/dL | mmol/L | 1 mmol/L = 6.01 mg/dL |
| Hemoglobin | g/dL | g/dL | g/L | 1 g/dL = 10 g/L |
| MCHC | g/dL | g/dL | g/dL | - |
| MCV | fL | fL | fl | - |
| PLT | K/uL | K/mcL | 109/L | 1 K/uL = 1 K/mcL = 1 x 109/L |
| RDW | % | % | % | - |
| WBC | K/uL | K/mcL | 109/L | 1 K/uL = 1 K/mcL = 1 x 109/L |
| TBil | mg/dL | mg/dL | umol/L | 1 mg/dL= 17.1 umol/L |
| AST | IU/L | IU/L | IU/L | - |
| Phosphate | mg/dL | mg/dL | mmol/L | 1 mmol/L = 3.097 mg/dL |
| Sepsis | - | - | - | - |
| AKI | - | - | - | - |
| Heart rate on the first day of ICU | bpm | bpm | bpm | - |
| SysBP on the first day of ICU | mmHg | mmHg | mmHg | - |
| Respiratory rate on the first day of ICU | beats/mins | beats/mins | beats/mins | - |
| SpO2 on the first day of ICU | % | % | % | - |

MCHC, mean corpuscular hemoglobin concentration; MCV, mean corpuscular volume; PLT, platelet count; RDW, red cell distribution width; WBC, white blood cells; TBil, total bilirubin; AST, aspartate aminotransferase; AKI, acute kidney injury.

**Table S3. The missing rate for each variable**

| Variables | Available quantity | Missing quantity | Missing percentage |
| --- | --- | --- | --- |
| All the patients (n=2559), patients underwent operations (n=1091), and patients admitted to the ICU (n=636) |  |  |  |
| Age | 2559 | 0 | 0 |
| Gender | 2559 | 0 | 0 |
| Length of stay in hospital | 2559 | 0 | 0 |
| Length of stay in ICU | 2559 | 0 | 0 |
| Death in hospital | 2559 | 0 | 0 |
| Re-admission within 30 days after discharge | 2559 | 0 | 0 |
| Re-admission within 60 days after discharge | 2559 | 0 | 0 |
| Re-admission within 90 days after discharge | 2559 | 0 | 0 |
| Follow-up after discharge until death | 2559 | 0 | 0 |
| Death within 180 days after discharge | 2559 | 0 | 0 |
| Death within 365 days after discharge | 2559 | 0 | 0 |
| Surgical operations | 2559 | 0 | 0 |
| Endoscopy | 2559 | 0 | 0 |
| Interventional operations | 2559 | 0 | 0 |
| Surgical or interventional or endoscopic operations | 2559 | 0 | 0 |
| Endoscopy combined with surgical operations | 2559 | 0 | 0 |
| Endoscopy combined with interventional operations | 2559 | 0 | 0 |
| Surgical or interventional operations other than endoscopy | 2559 | 0 | 0 |
| Endoscopic, surgical, and interventional procedures were performed during a single hospitalization | 2559 | 0 | 0 |
| Hypertension | 2559 | 0 | 0 |
| Diabetes | 2559 | 0 | 0 |
| Anion gap _ max | 2474 | 85 | 3.3 |
| Anion gap _ avg | 2474 | 85 | 3.3 |
| Anion gap _ min | 2474 | 85 | 3.3 |
| Bicarbonate _ max | 2474 | 85 | 3.3 |
| Bicarbonate _ avg | 2474 | 85 | 3.3 |
| Bicarbonate _ min | 2474 | 85 | 3.3 |
| Chloride _ max | 2477 | 82 | 3.2 |
| Chloride _ avg | 2477 | 82 | 3.2 |
| Chloride _ min | 2477 | 82 | 3.2 |
| Creatinine _ max | 2478 | 81 | 3.2 |
| Creatinine _ avg | 2478 | 81 | 3.2 |
| Creatinine _ min | 2478 | 81 | 3.2 |
| Glucose _ max | 2474 | 85 | 3.3 |
| Glucose _ avg | 2474 | 85 | 3.3 |
| Glucose _ min | 2474 | 85 | 3.3 |
| Potassium _ max | 2478 | 81 | 3.2 |
| Potassium _ avg | 2478 | 81 | 3.2 |
| Potassium _ min | 2478 | 81 | 3.2 |
| Sodium _ max | 2478 | 81 | 3.2 |
| Sodium _ avg | 2478 | 81 | 3.2 |
| Sodium _ min | 2478 | 81 | 3.2 |
| Urea nitrogen _ max | 2478 | 81 | 3.2 |
| Urea nitrogen _ avg | 2478 | 81 | 3.2 |
| Urea nitrogen _ min | 2478 | 81 | 3.2 |
| Hematocrit _ max | 2479 | 80 | 3.1 |
| Hematocrit _ avg | 2479 | 80 | 3.1 |
| Hematocrit _ min | 2479 | 80 | 3.1 |
| Hemoglobin _ max | 2478 | 81 | 3.2 |
| Hemoglobin _ avg | 2478 | 81 | 3.2 |
| Hemoglobin _ min | 2478 | 81 | 3.2 |
| MCH _ max | 2477 | 82 | 3.2 |
| MCH _ avg | 2477 | 82 | 3.2 |
| MCH _ min | 2477 | 82 | 3.2 |
| MCHC _ max | 2478 | 81 | 3.2 |
| MCHC _ avg | 2478 | 81 | 3.2 |
| MCHC _ min | 2478 | 81 | 3.2 |
| MCV _ max | 2477 | 82 | 3.2 |
| MCV _ avg | 2477 | 82 | 3.2 |
| MCV _ min | 2477 | 82 | 3.2 |
| PLT _ max | 2477 | 82 | 3.2 |
| PLT _ avg | 2477 | 82 | 3.2 |
| PLT _ min | 2477 | 82 | 3.2 |
| RDW _ max | 2477 | 82 | 3.2 |
| RDW _ avg | 2477 | 82 | 3.2 |
| RDW _ min | 2477 | 82 | 3.2 |
| RBC _ max | 2477 | 82 | 3.2 |
| RBC _ avg | 2477 | 82 | 3.2 |
| RBC _ min | 2477 | 82 | 3.2 |
| WBC _ max | 2478 | 81 | 3.2 |
| WBC _ avg | 2478 | 81 | 3.2 |
| WBC _ min | 2478 | 81 | 3.2 |
| TBil _ max | 2337 | 222 | 8.7 |
| TBil _ avg | 2337 | 222 | 8.7 |
| TBil _ min | 2337 | 222 | 8.7 |
| ALT _ max | 2352 | 207 | 8.1 |
| ALT _ avg | 2352 | 207 | 8.1 |
| ALT _ min | 2352 | 207 | 8.1 |
| ALP _ max | 2339 | 220 | 8.6 |
| ALP _ avg | 2339 | 220 | 8.6 |
| ALP _ min | 2339 | 220 | 8.6 |
| AST _ max | 2353 | 206 | 8.1 |
| AST _ avg | 2353 | 206 | 8.1 |
| AST _ min | 2353 | 206 | 8.1 |
| Magnesium _ max | 2404 | 155 | 6.1 |
| Magnesium _ avg | 2404 | 155 | 6.1 |
| Magnesium _ min | 2404 | 155 | 6.1 |
| Calcium _ max | 2395 | 164 | 6.4 |
| Calcium _ avg | 2395 | 164 | 6.4 |
| Calcium _ min | 2395 | 164 | 6.4 |
| Phosphate _ max | 2369 | 190 | 7.4 |
| Phosphate _ avg | 2369 | 190 | 7.4 |
| Phosphate _ min | 2369 | 190 | 7.4 |
| Patients admitted to the ICU (n=636) |  |  |  |
| SIRS score | 636 | 0 | 0 |
| SOFA score | 636 | 0 | 0 |
| Sepsis | 636 | 0 | 0 |
| AKI | 636 | 0 | 0 |
| Duration of auxiliary ventilation | 636 | 0 | 0 |
| Heart rate on the first day of ICU _ max | 635 | 1 | 0.2 |
| Heart rate on the first day of ICU _ avg | 635 | 1 | 0.2 |
| Heart rate on the first day of ICU _ min | 629 | 7 | 1.1 |
| SysBP on the first day of ICU _ max | 601 | 35 | 5.5 |
| SysBP on the first day of ICU _ avg | 601 | 35 | 5.5 |
| SysBP on the first day of ICU _ min | 601 | 35 | 5.5 |
| DiasBP on the first day of ICU _ max | 600 | 36 | 5.7 |
| DiasBP on the first day of ICU _ avg | 600 | 36 | 5.7 |
| DiasBP on the first day of ICU _ min | 600 | 36 | 5.7 |
| Mean arterial pressure on the first day of ICU admission _ max | 600 | 36 | 5.7 |
| Mean arterial pressure on the first day of ICU admission _ avg | 600 | 36 | 5.7 |
| Mean arterial pressure on the first day of ICU admission _ min | 599 | 37 | 5.8 |
| Respiratory rate on the first day of ICU _ max | 635 | 1 | 0.2 |
| Respiratory rate on the first day of ICU _ avg | 635 | 1 | 0.2 |
| Respiratory rate on the first day of ICU _ min | 602 | 34 | 5.3 |
| Temperature on the first day of ICU admission _ max | 609 | 27 | 4.2 |
| Temperature on the first day of ICU admission _ avg | 609 | 27 | 4.2 |
| Temperature on the first day of ICU admission _ min | 608 | 28 | 4.4 |
| SpO2 on the first day of ICU _ max | 635 | 1 | 0.2 |
| SpO2 on the first day of ICU _ avg | 635 | 1 | 0.2 |
| SpO2 on the first day of ICU _ min | 635 | 1 | 0.2 |

ICU, intensive care unit; MCH, mean corpuscular hemoglobin; MCHC, mean corpuscular hemoglobin concentration; MCV, mean corpuscular volume; PLT, platelet count; RDW, red cell distribution width; RBC, red blood cells; WBC, white blood cells; TBil, total bilirubin; ALT, alanine aminotransferase; ALP, alkaline phosphatase; AST, aspartate aminotransferase; SIRS, systemic inflammatory response syndrome; SOFA, sepsis-related organ failure; AKI, acute kidney injury; SysBP, systolic blood pressure; DiasBP, diastolic blood pressure; SpO2, saturation of peripheral oxygen.

**Table S4. Analysis of factors leading to different outcomes of patients underwent operations (n=1091)**

| Factors | Outcomes | | | | | |
| --- | --- | --- | --- | --- | --- | --- |
|  | Death in hospital P value | Re-admission within 30 days after discharge P value | Re-admission within 60 days after discharge P value | Re-admission within 90 days after discharge P value | Death within 180 days after discharge P value | Death within 365 days after discharge P value |
| Age | <0.001 | 0.429 | 0.157 | 0.097 | <0.001 | <0.001 |
| Gender | 1.000 | 0.767 | 0.529 | 0.321 | 0.116 | 0.029 |
| Length of stay in hospital | 0.003 | 0.023 | 0.006 | 0.002 | 0.001 | <0.001 |
| Length of stay in ICU | <0.001 | 0.838 | 0.416 | 0.553 | <0.001 | 0.003 |
| Surgical operations | 0.285 | 0.057 | 0.005 | 0.002 | <0.001 | <0.001 |
| Endoscopy | 1.000 | 1.000 | 0.709 | 0.554 | 0.082 | 0.038 |
| Interventional operations | 0.674 | 0.633 | 0.988 | 0.760 | 0.025 | 0.007 |
| Endoscopy combined with surgical operations | 0.068 | 0.021 | 0.006 | 0.005 | 0.065 | 0.031 |
| Endoscopy combined with interventional operations | 0.774 | 0.611 | 1.000 | 0.894 | 0.079 | 0.034 |
| Surgical or interventional operations other than endoscopy | 1.000 | 1.000 | 0.709 | 0.554 | 0.082 | 0.038 |
| Endoscopic, surgical, and interventional procedures were performed during a single hospitalization | 0.068 | 0.025 | 0.007 | 0.006 | 0.072 | 0.035 |
| Hypertension | 0.644 | 0.077 | 0.239 | 0.250 | 0.500 | 0.690 |
| Diabetes | 0.002 | 0.674 | 0.617 | 0.492 | 0.007 | 0.013 |
| Anion gap _ max | <0.001 | 0.010 | 0.014 | 0.033 | <0.001 | <0.001 |
| Anion gap _ avg | <0.001 | 0.152 | 0.588 | 0.676 | 0.028 | 0.028 |
| Anion gap _ min | 0.094 | 0.105 | 0.011 | 0.008 | 0.206 | 0.296 |
| Bicarbonate _ max | 0.909 | 0.052 | 0.006 | 0.007 | 0.805 | 0.637 |
| Bicarbonate _ avg | <0.001 | 0.725 | 0.199 | 0.196 | 0.024 | 0.042 |
| Bicarbonate _ min | <0.001 | 0.160 | 0.138 | 0.153 | <0.001 | <0.001 |
| Chloride _ max | <0.001 | 0.940 | 0.738 | 0.814 | 0.025 | 0.293 |
| Chloride _ avg | 0.416 | 0.017 | 0.007 | 0.007 | 0.607 | 0.327 |
| Chloride _ min | 0.007 | 0.001 | <0.001 | <0.001 | 0.199 | 0.005 |
| Creatinine _ max | <0.001 | 0.369 | 0.401 | 0.428 | <0.001 | <0.001 |
| Creatinine _ avg | <0.001 | 0.583 | 0.976 | 0.952 | <0.001 | <0.001 |
| Creatinine _ min | 0.204 | 0.506 | 0.281 | 0.217 | 0.017 | 0.046 |
| Glucose _ max | <0.001 | 0.044 | 0.010 | 0.046 | 0.003 | <0.001 |
| Glucose _ avg | <0.001 | 0.053 | 0.007 | 0.038 | 0.015 | 0.003 |
| Glucose _ min | 0.999 | 0.466 | 0.516 | 0.647 | 0.236 | 0.375 |
| Potassium _ max | <0.001 | 0.013 | 0.003 | 0.003 | 0.006 | 0.003 |
| Potassium _ avg | <0.001 | 0.273 | 0.033 | 0.038 | 0.712 | 0.609 |
| Potassium _ min | 0.111 | 0.137 | 0.495 | 0.445 | <0.001 | <0.001 |
| Sodium _ max | 0.060 | 0.066 | 0.226 | 0.205 | 0.023 | 0.261 |
| Sodium _ avg | 0.107 | 0.933 | 0.593 | 0.591 | 0.496 | 0.196 |
| Sodium _ min | <0.001 | 0.033 | 0.014 | 0.018 | 0.049 | <0.001 |
| Urea nitrogen _ max | <0.001 | 0.091 | 0.104 | 0.208 | <0.001 | <0.001 |
| Urea nitrogen _ avg | <0.001 | 0.178 | 0.149 | 0.274 | <0.001 | <0.001 |
| Urea nitrogen _ min | 0.001 | 0.757 | 0.867 | 0.717 | <0.001 | <0.001 |
| Hematocrit _ max | 0.503 | 0.941 | 0.710 | 0.736 | 0.315 | 0.078 |
| Hematocrit _ avg | <0.001 | 0.012 | 0.003 | 0.004 | <0.001 | <0.001 |
| Hematocrit _ min | <0.001 | 0.008 | 0.002 | 0.003 | <0.001 | <0.001 |
| Hemoglobin _ max | 0.455 | 0.543 | 0.290 | 0.248 | 0.069 | 0.017 |
| Hemoglobin _ avg | <0.001 | 0.004 | 0.001 | 0.001 | <0.001 | <0.001 |
| Hemoglobin _ min | <0.001 | 0.003 | 0.001 | 0.001 | <0.001 | <0.001 |
| MCH _ max | 0.046 | 0.683 | 0.630 | 0.836 | 0.125 | 0.162 |
| MCH _ avg | 0.259 | 0.451 | 0.446 | 0.325 | 0.868 | 0.902 |
| MCH _ min | 0.770 | 0.268 | 0.231 | 0.177 | 0.646 | 0.573 |
| MCHC _ max | 0.070 | 0.565 | 0.402 | 0.207 | 0.203 | 0.353 |
| MCHC _ avg | 0.160 | 0.012 | 0.002 | 0.001 | 0.002 | 0.007 |
| MCHC _ min | <0.001 | 0.005 | 0.001 | 0.001 | <0.001 | <0.001 |
| MCV _ max | <0.001 | 0.028 | 0.008 | 0.012 | 0.001 | 0.011 |
| MCV _ avg | 0.028 | 0.308 | 0.119 | 0.139 | 0.028 | 0.096 |
| MCV _ min | 0.484 | 0.954 | 0.585 | 0.612 | 0.385 | 0.586 |
| PLT _ max | 0.497 | <0.001 | <0.001 | <0.001 | 0.312 | 0.126 |
| PLT _ avg | 0.071 | 0.001 | <0.001 | <0.001 | 0.921 | 0.710 |
| PLT _ min | <0.001 | 0.105 | 0.095 | 0.068 | 0.076 | 0.204 |
| RDW _ max | <0.001 | 0.005 | <0.001 | <0.001 | <0.001 | <0.001 |
| RDW _ avg | <0.001 | 0.018 | 0.001 | <0.001 | <0.001 | <0.001 |
| RDW _ min | 0.002 | 0.143 | 0.019 | 0.005 | <0.001 | <0.001 |
| RBC _ max | 0.161 | 0.831 | 0.485 | 0.404 | 0.023 | 0.010 |
| RBC _ avg | <0.001 | 0.011 | 0.001 | 0.001 | <0.001 | <0.001 |
| RBC _ min | <0.001 | 0.004 | 0.001 | 0.001 | <0.001 | <0.001 |
| WBC _ max | <0.001 | 0.015 | 0.002 | 0.006 | 0.112 | 0.038 |
| WBC _ avg | <0.001 | 0.019 | 0.005 | 0.010 | 0.435 | 0.284 |
| WBC _ min | 0.751 | 0.046 | 0.035 | 0.034 | 0.388 | 0.348 |
| TBil _ max | 0.004 | 0.763 | 0.532 | 0.624 | <0.001 | <0.001 |
| TBil _ avg | 0.024 | 0.677 | 0.766 | 0.670 | <0.001 | <0.001 |
| TBil _ min | 0.378 | 0.078 | 0.069 | 0.057 | 0.010 | 0.001 |
| ALT _max | 0.429 | 0.290 | 0.132 | 0.045 | 0.904 | 0.784 |
| ALT _avg | 0.610 | 0.029 | 0.007 | 0.001 | 0.464 | 0.315 |
| ALT _ min | 0.002 | 0.002 | <0.001 | <0.001 | 0.078 | 0.058 |
| ALP _ max | 0.345 | 0.414 | 0.271 | 0.259 | <0.001 | <0.001 |
| ALP _ avg | 0.695 | 0.703 | 0.984 | 0.996 | <0.001 | <0.001 |
| ALP _ min | 0.006 | 0.120 | 0.253 | 0.220 | 0.002 | <0.001 |
| AST _max | <0.001 | 0.637 | 0.610 | 0.314 | 0.022 | 0.040 |
| AST _avg | 0.015 | 0.134 | 0.138 | 0.052 | 0.054 | 0.099 |
| AST _min | 0.779 | 0.012 | 0.006 | 0.002 | 0.277 | 0.430 |
| Magnesium _ max | <0.001 | 0.001 | 0.001 | 0.002 | 0.001 | 0.001 |
| Magnesium _ avg | 0.001 | 0.008 | 0.033 | 0.045 | 0.084 | 0.332 |
| Magnesium _ min | 0.001 | 0.222 | 0.112 | 0.085 | 0.117 | 0.020 |
| Calcium _ max | 0.288 | 0.710 | 0.902 | 0.989 | 0.493 | 0.899 |
| Calcium _ avg | <0.001 | 0.031 | 0.003 | 0.006 | 0.002 | 0.013 |
| Calcium _ min | <0.001 | 0.002 | <0.001 | <0.001 | <0.001 | <0.001 |
| Phosphate _ max | <0.001 | 0.160 | 0.037 | 0.027 | 0.024 | 0.044 |
| Phosphate _ avg | <0.001 | 0.203 | 0.114 | 0.064 | 0.159 | 0.223 |
| Phosphate _ min | 0.009 | 0.137 | 0.069 | 0.167 | 0.116 | 0.150 |

ICU, intensive care unit; MCH, mean corpuscular hemoglobin; MCHC, mean corpuscular hemoglobin concentration; MCV, mean corpuscular volume; PLT, platelet count; RDW, red cell distribution width; RBC, red blood cells; WBC, white blood cells; TBil, total bilirubin; ALT, alanine aminotransferase; ALP, alkaline phosphatase; AST, aspartate aminotransferase.

**Table S5. Analysis of factors leading to different outcomes of patients admitted to the ICU (n=636)**

| Factors | Outcomes | | | | | |
| --- | --- | --- | --- | --- | --- | --- |
|  | Death in hospital P value | Re-admission within 30 days after discharge P value | Re-admission within 60 days after discharge P value | Re-admission within 90 days after discharge P value | Death within 180 days after discharge P value | Death within 365 days after discharge P value |
| Age | <0.001 | 0.444 | 0.246 | 0.091 | <0.001 | <0.001 |
| Gender | 0.580 | 0.752 | 0.440 | 0.301 | 0.704 | 0.556 |
| Length of stay in hospital | 0.334 | 0.010 | 0.001 | <0.001 | 0.144 | 0.205 |
| Length of stay in ICU | 0.009 | 0.510 | 0.347 | 0.185 | 0.327 | 0.526 |
| Surgical operations | 0.770 | 0.600 | 0.522 | 0.542 | 0.661 | 0.922 |
| Endoscopy | 0.156 | 1.000 | 0.608 | 1.000 | 0.078 | 0.155 |
| Interventional operations | 0.116 | 1.000 | 0.797 | 1.000 | 0.071 | 0.067 |
| Surgical or interventional or endoscopic operations | 0.355 | 0.586 | 0.305 | 0.722 | 0.261 | 0.223 |
| Endoscopy combined with surgical operations | 0.108 | 0.715 | 1.000 | 0.773 | 0.750 | 0.767 |
| Endoscopy combined with interventional operations | 0.131 | 1.000 | 0.754 | 1.000 | 0.062 | 0.110 |
| Surgical or interventional operations other than endoscopy | 0.423 | 0.268 | 0.235 | 0.401 | 0.348 | 1.000 |
| Endoscopic, surgical, and interventional procedures were performed during a single hospitalization | 0.108 | 0.715 | 1.000 | 0.773 | 0.750 | 0.767 |
| Hypertension | 0.261 | 0.021 | 0.169 | 0.140 | 0.122 | 0.063 |
| Diabetes | 0.794 | 0.697 | 0.604 | 0.540 | 0.746 | 0.411 |
| Anion gap _ max | <0.001 | 0.155 | 0.144 | 0.045 | 0.099 | 0.048 |
| Anion gap _ avg | <0.001 | 0.033 | 0.010 | 0.005 | 0.120 | 0.094 |
| Anion gap _ min | 0.005 | 0.005 | <0.001 | <0.001 | 0.890 | 0.887 |
| Bicarbonate _ max | <0.001 | 0.002 | <0.001 | <0.001 | 0.100 | 0.110 |
| Bicarbonate _ avg | <0.001 | 0.035 | 0.003 | 0.001 | 0.019 | 0.011 |
| Bicarbonate _ min | <0.001 | 0.289 | 0.097 | 0.052 | 0.026 | 0.016 |
| Chloride _ max | 0.012 | 0.735 | 0.729 | 0.940 | 0.004 | 0.006 |
| Chloride _ avg | 0.344 | 0.132 | 0.172 | 0.091 | 0.010 | 0.008 |
| Chloride _ min | 0.049 | 0.024 | 0.018 | 0.007 | 0.079 | 0.059 |
| Creatinine _ max | <0.001 | 0.141 | 0.013 | 0.001 | 0.001 | 0.002 |
| Creatinine _ avg | <0.001 | 0.033 | 0.001 | <0.001 | 0.001 | 0.003 |
| Creatinine _ min | <0.001 | 0.006 | <0.001 | <0.001 | 0.008 | 0.031 |
| Glucose _ max | <0.001 | 0.187 | 0.049 | 0.053 | 0.046 | 0.065 |
| Glucose _ avg | <0.001 | 0.055 | 0.052 | 0.028 | 0.451 | 0.646 |
| Glucose _ min | 0.826 | 0.601 | 0.570 | 0.841 | 0.037 | 0.040 |
| Potassium _ max | <0.001 | 0.605 | 0.302 | 0.066 | 0.176 | 0.245 |
| Potassium _ avg | <0.001 | 0.964 | 0.497 | 0.269 | 0.512 | 0.580 |
| Potassium _ min | 0.316 | 0.371 | 0.717 | 0.523 | 0.148 | 0.116 |
| Sodium _ max | 0.038 | 0.743 | 0.612 | 0.894 | 0.034 | 0.037 |
| Sodium _ avg | 0.389 | 0.453 | 0.512 | 0.271 | 0.040 | 0.033 |
| Sodium _ min | 0.038 | 0.094 | 0.054 | 0.017 | 0.697 | 0.539 |
| Urea nitrogen _ max | <0.001 | 0.741 | 0.205 | 0.069 | <0.001 | <0.001 |
| Urea nitrogen _ avg | <0.001 | 0.454 | 0.101 | 0.029 | <0.001 | <0.001 |
| Urea nitrogen _ min | <0.001 | 0.186 | 0.035 | 0.013 | <0.001 | <0.001 |
| Hematocrit _ max | 0.279 | 0.150 | 0.288 | 0.430 | 0.959 | 0.452 |
| Hematocrit _ avg | 0.003 | 0.155 | 0.084 | 0.071 | 0.336 | 0.222 |
| Hematocrit _ min | <0.001 | 0.255 | 0.126 | 0.141 | 0.084 | 0.061 |
| Hemoglobin _ max | 0.137 | 0.283 | 0.690 | 0.995 | 0.874 | 0.462 |
| Hemoglobin _ avg | 0.001 | 0.051 | 0.013 | 0.009 | 0.388 | 0.261 |
| Hemoglobin _ min | <0.001 | 0.245 | 0.090 | 0.075 | 0.141 | 0.099 |
| MCH _ max | 0.717 | 0.243 | 0.182 | 0.076 | 0.606 | 0.951 |
| MCH _ avg | 0.730 | 0.049 | 0.024 | 0.009 | 0.204 | 0.531 |
| MCH _ min | 0.710 | 0.035 | 0.012 | 0.005 | 0.152 | 0.450 |
| MCHC _ max | 0.612 | 0.197 | 0.133 | 0.028 | 0.336 | 0.592 |
| MCHC _ avg | 0.037 | 0.011 | 0.001 | <0.001 | 0.969 | 0.984 |
| MCHC _ min | <0.001 | 0.027 | 0.002 | <0.001 | 0.248 | 0.301 |
| MCV _ max | 0.001 | 0.881 | 0.939 | 0.983 | 0.998 | 0.718 |
| MCV _ avg | 0.101 | 0.470 | 0.691 | 0.585 | 0.506 | 0.954 |
| MCV _ min | 0.537 | 0.393 | 0.446 | 0.390 | 0.137 | 0.522 |
| PLT _ max | <0.001 | <0.001 | <0.001 | <0.001 | 0.106 | 0.063 |
| PLT _ avg | <0.001 | <0.001 | <0.001 | <0.001 | 0.036 | 0.024 |
| PLT _ min | <0.001 | 0.004 | 0.004 | <0.001 | 0.015 | 0.022 |
| RDW _ max | <0.001 | 0.463 | 0.942 | 0.969 | 0.001 | 0.004 |
| RDW _ avg | <0.001 | 0.125 | 0.447 | 0.363 | <0.001 | 0.002 |
| RDW _ min | <0.001 | 0.048 | 0.284 | 0.187 | <0.001 | 0.002 |
| RBC _ max | 0.290 | 0.127 | 0.375 | 0.593 | 0.493 | 0.872 |
| RBC _ avg | 0.006 | 0.396 | 0.193 | 0.162 | 0.712 | 0.438 |
| RBC _ min | <0.001 | 0.525 | 0.329 | 0.373 | 0.198 | 0.119 |
| WBC _ max | <0.001 | 0.002 | 0.006 | 0.004 | 0.744 | 0.900 |
| WBC _ avg | <0.001 | 0.002 | 0.011 | 0.002 | 0.837 | 0.493 |
| WBC _ min | 0.067 | 0.103 | 0.529 | 0.176 | 0.568 | 0.404 |
| TBil _ max | <0.001 | 0.909 | 0.627 | 0.732 | 0.066 | 0.070 |
| TBil _ avg | <0.001 | 0.543 | 0.782 | 0.259 | 0.046 | 0.052 |
| TBil _ min | <0.001 | 0.124 | 0.134 | 0.031 | 0.152 | 0.126 |
| ALT _max | 0.154 | 0.428 | 0.480 | 0.290 | 0.026 | 0.088 |
| ALT _avg | 0.449 | 0.085 | 0.100 | 0.039 | 0.120 | 0.307 |
| ALT _ min | 0.451 | 0.022 | 0.019 | 0.003 | 0.715 | 0.976 |
| ALP _ max | 0.249 | 0.670 | 0.679 | 0.950 | 0.022 | 0.049 |
| ALP _ avg | 0.745 | 0.271 | 0.893 | 0.645 | 0.046 | 0.085 |
| ALP _ min | 0.130 | 0.068 | 0.402 | 0.263 | 0.292 | 0.356 |
| AST _max | <0.001 | 0.327 | 0.581 | 0.282 | 0.046 | 0.084 |
| AST _avg | <0.001 | 0.081 | 0.156 | 0.047 | 0.081 | 0.143 |
| AST _min | <0.001 | 0.012 | 0.006 | 0.001 | 0.676 | 0.667 |
| Magnesium _ max | <0.001 | 0.175 | 0.327 | 0.267 | 0.068 | 0.045 |
| Magnesium _ avg | <0.001 | 0.459 | 0.818 | 0.686 | 0.031 | 0.063 |
| Magnesium _ min | 0.985 | 0.578 | 0.372 | 0.852 | 0.229 | 0.561 |
| Calcium _ max | 0.289 | 0.071 | 0.143 | 0.101 | 0.533 | 0.569 |
| Calcium _ avg | 0.061 | 0.007 | 0.005 | 0.004 | 0.603 | 0.846 |
| Calcium _ min | <0.001 | 0.154 | 0.050 | 0.033 | 0.153 | 0.436 |
| Phosphate _ max | <0.001 | 0.998 | 0.620 | 0.406 | 0.011 | 0.014 |
| Phosphate _ avg | <0.001 | 0.968 | 0.472 | 0.370 | 0.004 | 0.008 |
| Phosphate _ min | 0.007 | 0.682 | 0.981 | 0.902 | 0.286 | 0.429 |
| SIRS score | 0.001 | 0.212 | 0.196 | 0.114 | 0.935 | 0.779 |
| SOFA score | <0.001 | 0.038 | 0.013 | 0.001 | 0.013 | 0.071 |
| Sepsis | <0.001 | 0.060 | 0.065 | 0.013 | 0.282 | 0.287 |
| AKI | <0.001 | 0.070 | 0.165 | 0.664 | 0.594 | 1.000 |
| Duration of auxiliary ventilation | <0.001 | 0.976 | 0.380 | 0.657 | 0.618 | 0.632 |
| Heart rate on the first day of ICU _ max | 0.054 | 0.071 | 0.025 | 0.001 | 0.003 | 0.011 |
| Heart rate on the first day of ICU _ avg | 0.007 | 0.040 | 0.027 | 0.001 | 0.001 | 0.002 |
| Heart rate on the first day of ICU _ min | 0.225 | 0.061 | 0.020 | 0.001 | 0.014 | 0.042 |
| SysBP on the first day of ICU _ max | <0.001 | 0.003 | 0.006 | 0.002 | 0.437 | 0.823 |
| SysBP on the first day of ICU _ avg | <0.001 | 0.022 | 0.036 | 0.029 | 0.379 | 0.416 |
| SysBP on the first day of ICU _ min | <0.001 | 0.004 | 0.003 | 0.001 | 0.244 | 0.795 |
| DiasBP on the first day of ICU _ max | <0.001 | 0.002 | 0.002 | <0.001 | 0.027 | 0.071 |
| DiasBP on the first day of ICU _ avg | 0.079 | 0.422 | 0.617 | 0.411 | 0.172 | 0.246 |
| DiasBP on the first day of ICU _ min | <0.001 | 0.001 | <0.001 | <0.001 | 0.092 | 0.254 |
| Mean arterial pressure on the first day of ICU admission _ max | <0.001 | 0.001 | 0.002 | <0.001 | 0.072 | 0.236 |
| Mean arterial pressure on the first day of ICU admission _ avg | 0.021 | 0.180 | 0.271 | 0.221 | 0.258 | 0.474 |
| Mean arterial pressure on the first day of ICU admission _ min | <0.001 | <0.001 | <0.001 | <0.001 | 0.207 | 0.732 |
| Respiratory rate on the first day of ICU _ max | <0.001 | 0.233 | 0.067 | 0.027 | 0.160 | 0.112 |
| Respiratory rate on the first day of ICU _ avg | <0.001 | 0.063 | 0.009 | 0.001 | 0.035 | 0.050 |
| Respiratory rate on the first day of ICU _ min | 0.224 | 0.338 | 0.368 | 0.800 | 0.676 | 0.676 |
| Temperature on the first day of ICU admission _ max | 0.001 | 0.017 | 0.002 | <0.001 | 0.029 | 0.012 |
| Temperature on the first day of ICU admission _ avg | 0.009 | 0.051 | 0.006 | 0.001 | 0.249 | 0.178 |
| Temperature on the first day of ICU admission _ min | 0.001 | 0.008 | 0.001 | <0.001 | 0.004 | 0.003 |
| SpO2 on the first day of ICU _ max | <0.001 | 0.144 | 0.101 | 0.101 | 0.146 | 0.045 |
| SpO2 on the first day of ICU _ avg | 0.931 | 0.199 | 0.332 | 0.441 | 0.016 | 0.003 |
| SpO2 on the first day of ICU _ min | <0.001 | 0.284 | 0.228 | 0.329 | 0.099 | 0.053 |

ICU, intensive care unit; MCH, mean corpuscular hemoglobin; MCHC, mean corpuscular hemoglobin concentration; MCV, mean corpuscular volume; PLT, platelet count; RDW, red cell distribution width; RBC, red blood cells; WBC, white blood cells; TBil, total bilirubin; ALT, alanine aminotransferase; ALP, alkaline phosphatase; AST, aspartate aminotransferase; SIRS, systemic inflammatory response syndrome; SOFA, sepsis-related organ failure; AKI, acute kidney injury; SysBP, systolic blood pressure; DiasBP, diastolic blood pressure.

**Table S6. Influencing factors of different outcomes corresponding to various patient categories (P＜0.1) of all the patients (n=2559)**

| In-hospital mortality | Re-admission within 30 days after discharge | Re-admission within 60 days after discharge | Re-admission within 90 days after discharge | Mortality within 180 days after discharge | Mortality within 365 days after discharge |
| --- | --- | --- | --- | --- | --- |
| RBC _ min | PLT _ max | Age | Age | Hemoglobin _ max | Hemoglobin _ max |
| Phosphate _ avg | PLT _ avg | Length of stay in hospital | Gender | ALP _ min | ALP _ min |
| Calcium _ min | PLT _ min | Surgical operations | Length of stay in hospital | RBC _ min | RBC _ min |
| Phosphate _ max | Bicarbonate _ avg | Interventional operations | Surgical operations | Calcium _ min | Calcium _ min |
| Calcium _ avg | Age | Surgical or interventional or endoscopic operations | Endoscopy | Phosphate _ max | ALP _ avg |
| AST _avg | AST _min | Endoscopy combined with surgical operations | Interventional operations | ALP _ avg | ALP _ max |
| Magnesium _ max | Bicarbonate _ max | Endoscopy combined with interventional operations | Surgical or interventional or endoscopic operations | ALP _ max | TBil _ avg |
| Magnesium _ avg | Chloride _ avg | Endoscopic, surgical, and interventional procedures were performed during a single hospitalization | Endoscopy combined with surgical operations | RBC _ avg | TBil _ max |
| AST _max | Surgical or interventional or endoscopic operations | Anion gap _ avg | Endoscopy combined with interventional operations | RDW _ min | RBC _ avg |
| ALP _ max | Bicarbonate _ min | Anion gap _ min | Endoscopic, surgical, and interventional procedures were performed during a single hospitalization | RDW _ avg | RBC _ max |
| WBC _ avg | Endoscopy combined with interventional operations | Bicarbonate _ max | Anion gap _ avg | RDW _ max | RDW _ min |
| TBil _ min | Surgical operations | Bicarbonate _ avg | Anion gap _ min | MCHC _ min | RDW _ avg |
| TBil _ avg | Interventional operations | Bicarbonate _ min | Bicarbonate _ max | Hemoglobin _ min | RDW _ max |
| TBil _ max | Phosphate _ avg | Chloride _ avg | Bicarbonate _ avg | Hemoglobin _ avg | MCHC _ min |
| RBC _ avg | Chloride _ min | Chloride _ min | Bicarbonate _ min | Hematocrit _ min | Hemoglobin _ min |
| RDW _ min | Endoscopy combined with surgical operations | Creatinine _ avg | Chloride _ avg | Urea nitrogen _ max | Hemoglobin _ avg |
| RDW _ avg | MCHC _ avg | Glucose _ max | Chloride _ min | Hematocrit _ avg | Hematocrit _ min |
| PLT _ avg | Endoscopic, surgical, and interventional procedures were performed during a single hospitalization | Potassium _ avg | Creatinine _ max | Urea nitrogen _ avg | Urea nitrogen _ max |
| RDW _ max | TBil _ avg | Potassium _ min | Creatinine _ avg | Urea nitrogen _ min | Hematocrit _ avg |
| PLT _ min | TBil _ min | Sodium _ min | Creatinine _ min | Bicarbonate _ avg | Urea nitrogen _ avg |
| MCV _ max | Endoscopy | Urea nitrogen _ avg | Glucose _ max | Sodium _ max | Urea nitrogen _ min |
| MCHC _ min | AST _avg | Urea nitrogen _ min | Glucose _ avg | Potassium _ min | Hematocrit _ max |
| Hemoglobin _ min | Urea nitrogen _ min | MCH _ avg | Potassium _ avg | Potassium _ max | Bicarbonate _ avg |
| Hemoglobin _ avg | Anion gap _ avg | MCH _ min | Sodium _ avg | Bicarbonate _ min | Potassium _ min |
| Sodium _ min | Potassium _ avg | MCHC _ max | Sodium _ min | Chloride _ max | Bicarbonate _ min |
| Hematocrit _ min | Phosphate _ min | MCHC _ avg | Urea nitrogen _ avg | Creatinine _ avg | Chloride _ max |
| Urea nitrogen _ max | Anion gap _ min | MCHC _ min | Urea nitrogen _ min | Creatinine _ max | Creatinine _ avg |
| Hematocrit _ avg | MCHC _ min | PLT _ max | MCHC _ avg | Anion gap _ avg | Creatinine _ max |
| Urea nitrogen _ avg | Length of stay in hospital | PLT _ avg | MCHC _ min | Anion gap _ max | Anion gap _ max |
| Urea nitrogen _ min | Creatinine _ avg | PLT _ min | PLT _ max | Age | Interventional operations |
| Bicarbonate _ avg | MCH _ avg | TBil _ max | PLT _ avg | Length of stay in hospital | Age |
| Sodium _ max | Sodium _ min | TBil _ avg | PLT _ min | TBil _ avg | Length of stay in hospital |
| Potassium _ avg | Creatinine _ min | TBil _ min | TBil _ max | TBil _ max | Phosphate _ max |
| Potassium _ max | MCHC _ max | ALT _max | TBil _ avg | Hematocrit _ max | TBil _ min |
| Bicarbonate _ min | ALT _avg | ALT _avg | TBil _ min | Creatinine _ min | Sodium _ min |
| Creatinine _ min | Potassium _ min | AST _max | ALT _max | Length of stay in ICU | Sodium _ max |
| Glucose _ avg | Urea nitrogen _ avg | AST _avg | ALT _avg | MCHC _ avg | Anion gap _ avg |
| Glucose _ max | RDW _ avg | AST _min | ALT _ min | RBC _ max | Endoscopy combined with interventional operations |
| Chloride _ max | MCH _ min | Phosphate _ avg | ALP _ avg | Calcium _ avg | Endoscopy |
| Chloride _ min | Chloride _ max | Phosphate _ min | ALP _ min | Magnesium _ max | Potassium _ max |
| Creatinine _ avg | ALP _ avg |  | AST _max | Diabetes | Creatinine _ min |
| Creatinine _ max | TBil _ max |  | AST _avg | Glucose _ max | Magnesium _ max |
| Anion gap _ avg | MCH _ max |  | AST _min | MCV _ max | Calcium _ avg |
| Anion gap _ max |  |  | Phosphate _ max | Surgical operations | MCV _ max |
| Length of stay in ICU |  |  | Phosphate _ avg | Sodium _ min | Diabetes |
| Age |  |  | Phosphate _ min | TBil _ min | Surgical or interventional or endoscopic operations |
| Length of stay in hospital |  |  |  | Phosphate _ min | Magnesium _ min |
| Bicarbonate _ max |  |  |  | PLT _ min | MCHC _ avg |
| Magnesium _ min |  |  |  | WBC _ max | Length of stay in ICU |
| AST _min |  |  |  | Magnesium _ min | Glucose _ max |
| Diabetes |  |  |  | WBC _ avg | PLT _ min |
| ALT _max |  |  |  | Interventional operations | Surgical operations |
| MCHC _ avg |  |  |  | Glucose _ min | Phosphate _ min |
| Potassium _ min |  |  |  | Endoscopy combined with surgical operations | Glucose _ avg |
| WBC _ min |  |  |  | Endoscopic, surgical, and interventional procedures were performed during a single hospitalization | WBC _ avg |
| ALT _avg |  |  |  | Endoscopy | Chloride _ min |
| MCH _ max |  |  |  | Glucose _ avg | MCV _ avg |
| MCV _ avg |  |  |  | Chloride _ avg | WBC _ max |
| Phosphate _ min |  |  |  | Endoscopy combined with interventional operations | Endoscopy combined with surgical operations |
| ALT _ min |  |  |  | Phosphate _ avg | Endoscopic, surgical, and interventional procedures were performed during a single hospitalization |
| Calcium _ max |  |  |  |  |  |
| Hemoglobin _ max |  |  |  |  |  |
| Anion gap _ min |  |  |  |  |  |
| RBC _ max |  |  |  |  |  |
| ALP _ avg |  |  |  |  |  |
| MCHC _ max |  |  |  |  |  |

ICU, intensive care unit; MCH, mean corpuscular hemoglobin; MCHC, mean corpuscular hemoglobin concentration; MCV, mean corpuscular volume; PLT, platelet count; RDW, red cell distribution width; RBC, red blood cells; WBC, white blood cells; TBil, total bilirubin; ALT, alanine aminotransferase; ALP, alkaline phosphatase; AST, aspartate aminotransferase; SIRS, systemic inflammatory response syndrome; SOFA, sepsis-related organ failure; AKI, acute kidney injury; SysBP, systolic blood pressure; DiasBP, diastolic blood pressure; SpO2, saturation of peripheral oxygen.

**Table S7. Influencing factors of different outcomes corresponding to various patient categories (P＜0.1) of patients underwent operations (n=1091)**

| In-hospital mortality | Re-admission within 30 days after discharge | Re-admission within 60 days after discharge | Re-admission within 90 days after discharge | Mortality within 180 days after discharge | Mortality within 365 days after discharge |
| --- | --- | --- | --- | --- | --- |
| Phosphate _ avg | PLT _ avg | Age | Age | ALP _ min | ALP _ min |
| Phosphate _ max | PLT _ max | Length of stay in hospital | Length of stay in hospital | ALP _ avg | ALP _ avg |
| Calcium _ min | MCHC _ min | Surgical operations | Surgical operations | ALP _ max | ALP _ max |
| Calcium _ avg | MCHC _ avg | Surgical or interventional or endoscopic operations | Surgical or interventional or endoscopic operations | RBC _ min | TBil _ max |
| ALT _max | Surgical or interventional or endoscopic operations | Endoscopy combined with surgical operations | Endoscopy combined with surgical operations | RBC _ avg | RBC _ min |
| TBil _ min | Sodium _ min | Endoscopic, surgical, and interventional procedures were performed during a single hospitalization | Endoscopic, surgical, and interventional procedures were performed during a single hospitalization | RDW _ min | RBC _ avg |
| TBil _ avg | Glucose _ max | Anion gap _ min | Anion gap _ min | RDW _ avg | RDW _ min |
| TBil _ max | Chloride _ min | Bicarbonate _ max | Bicarbonate _ max | RDW _ max | RDW _ avg |
| WBC _ avg | Length of stay in hospital | Chloride _ avg | Chloride _ avg | Hemoglobin _ min | RDW _ max |
| WBC _ max | Magnesium _ max | Chloride _ min | Chloride _ min | Hemoglobin _ avg | Hemoglobin _ min |
| RBC _ min | MCV _ max | Glucose _ max | Glucose _ max | Hematocrit _ min | Hemoglobin _ avg |
| RBC _ avg | PLT _ min | Glucose _ avg | Glucose _ avg | Hematocrit _ avg | Hematocrit _ min |
| RDW _ avg | Chloride _ avg | Potassium _ max | Potassium _ max | Urea nitrogen _ min | Hematocrit _ avg |
| RDW _ max | Hemoglobin _ min | Potassium _ avg | Potassium _ avg | Urea nitrogen _ avg | Urea nitrogen _ min |
| PLT _ min | Potassium _ max | Sodium _ avg | Sodium _ avg | Urea nitrogen _ max | Urea nitrogen _ avg |
| MCV _ max | Hemoglobin _ avg | Sodium _ min | Sodium _ min | Potassium _ min | Urea nitrogen _ max |
| MCHC _ min | Urea nitrogen _ max | Urea nitrogen _ max | Urea nitrogen _ max | Anion gap _ max | Potassium _ min |
| Hemoglobin _ min | Glucose _ avg | Hematocrit _ avg | Hematocrit _ avg | Surgical or interventional or endoscopic operations | Surgical or interventional or endoscopic operations |
| Hematocrit _ min | RBC _ min | Hematocrit _ min | Hematocrit _ min | Age | Surgical operations |
| Urea nitrogen _ avg | Bicarbonate _ max | Hemoglobin _ avg | Hemoglobin _ avg | Creatinine _ max | Age |
| Urea nitrogen _ max | Calcium _ min | Hemoglobin _ min | Hemoglobin _ min | Diabetes | Anion gap _ max |
| Sodium _ min | WBC _ max | MCHC _ max | MCHC _ max | Surgical operations | Diabetes |
| Potassium _ avg | RDW _ max | MCHC _ avg | MCHC _ avg | Calcium _ min | Calcium _ min |
| Potassium _ max | RBC _ avg | MCHC _ min | MCHC _ min | TBil _ max | TBil _ avg |
| Creatinine _ max | Phosphate _ max | MCV _ max | MCV _ max | MCHC _ min | MCHC _ min |
| Bicarbonate _ min | Length of stay in ICU | MCV _ avg | MCV _ avg | Bicarbonate _ min | Length of stay in hospital |
| Bicarbonate _ avg | Endoscopy combined with surgical operations | PLT _ max | PLT _ max | MCV _ max | Bicarbonate _ min |
| Anion gap _ avg | AST _min | PLT _ avg | PLT _ avg | Chloride _ max | Sodium _ min |
| Anion gap _ max | AST _avg | PLT _ min | PLT _ min | Length of stay in hospital | MCV _ max |
| Surgical or interventional or endoscopic operations | Endoscopic, surgical, and interventional procedures were performed during a single hospitalization | RDW _ max | RDW _ max | Sodium _ max | RBC _ max |
| AST _max | Hematocrit _ min | RDW _ avg | RDW _ avg | Anion gap _ avg | Anion gap _ avg |
| Glucose _ max | MCHC _ max | RDW _ min | RDW _ min | Calcium _ avg | Creatinine _ max |
| Creatinine _ avg |  | RBC _ avg | RBC _ avg | RBC _ max | Hemoglobin _ max |
| Chloride _ min |  | RBC _ min | RBC _ min | Creatinine _ avg | Calcium _ avg |
| Chloride _ max |  | WBC _ max | WBC _ max | TBil _ avg | Endoscopy combined with surgical operations |
| Age |  | WBC _ avg | WBC _ avg | Bicarbonate _ avg | Endoscopic, surgical, and interventional procedures were performed during a single hospitalization |
| Magnesium _ min |  | AST _min | ALT _avg | Hemoglobin _ max | Bicarbonate _ avg |
| AST _avg |  | Magnesium _ max | AST _avg | MCHC _ avg | TBil _ min |
| ALT _avg |  | Calcium _ avg | AST _min | MCV _ avg | MCHC _ avg |
| Hemoglobin _ avg |  | Calcium _ min | Magnesium _ max | Length of stay in ICU | Chloride _ min |
| Hematocrit _ avg |  | Phosphate _ max | Calcium _ avg | Endoscopy combined with surgical operations | MCV _ avg |
| Urea nitrogen _ min |  | Phosphate _ avg | Calcium _ min | Endoscopic, surgical, and interventional procedures were performed during a single hospitalization | Creatinine _ avg |
| Length of stay in ICU |  |  | Phosphate _ max | AST _avg | Sodium _ max |
| Magnesium _ avg |  |  | Phosphate _ avg | AST _max | Interventional operations |
| ALP _ max |  |  |  | Creatinine _ min | Magnesium _ min |
| Diabetes |  |  |  | Hematocrit _ max | Hematocrit _ max |
| RDW _ min |  |  |  | TBil _ min | Length of stay in ICU |
| Sodium _ avg |  |  |  | Hypertension | Gender |
| Glucose _ avg |  |  |  |  | Phosphate _ max |
| Phosphate _ min |  |  |  |  | Glucose _ avg |
| Magnesium _ max |  |  |  |  | Chloride _ max |
| Sodium _ max |  |  |  |  | Creatinine _ min |
| Length of stay in hospital |  |  |  |  | Surgical or interventional operations other than endoscopy |
| AST _min |  |  |  |  | Endoscopy |
| MCV _ avg |  |  |  |  | Magnesium _ max |
| PLT _ avg |  |  |  |  | Endoscopy combined with interventional operations |
| ALT _ min |  |  |  |  |  |

ICU, intensive care unit; MCH, mean corpuscular hemoglobin; MCHC, mean corpuscular hemoglobin concentration; MCV, mean corpuscular volume; PLT, platelet count; RDW, red cell distribution width; RBC, red blood cells; WBC, white blood cells; TBil, total bilirubin; ALT, alanine aminotransferase; ALP, alkaline phosphatase; AST, aspartate aminotransferase; SIRS, systemic inflammatory response syndrome; SOFA, sepsis-related organ failure; AKI, acute kidney injury; SysBP, systolic blood pressure; DiasBP, diastolic blood pressure; SpO2, saturation of peripheral oxygen.

**Table S8. Influencing factors of different outcomes corresponding to various patient categories (P＜0.1) of patients admitted to the ICU (n=636)**

| In-hospital mortality | Re-admission within 30 days after discharge | Re-admission within 60 days after discharge | Re-admission within 90 days after discharge | Mortality within 180 days after discharge | Mortality within 365 days after discharge |
| --- | --- | --- | --- | --- | --- |
| Age | Length of stay in hospital | Anion gap _ avg | Anion gap _ max | Age | Age |
| Anion gap _ max | Hypertension | Anion gap _ min | Anion gap _ avg | Anion gap _ avg | Anion gap _ avg |
| Anion gap _ avg | Anion gap _ max | Bicarbonate _ max | Anion gap _ min | Bicarbonate _ max | Bicarbonate _ max |
| Anion gap _ min | Anion gap _ avg | Bicarbonate _ avg | Bicarbonate _ max | Bicarbonate _ avg | Bicarbonate _ avg |
| Bicarbonate _ max | Anion gap _ min | Creatinine _ max | Bicarbonate _ avg | Bicarbonate _ min | Bicarbonate _ min |
| Bicarbonate _ avg | Bicarbonate _ max | Creatinine _ avg | Chloride _ min | Chloride _ max | Chloride _ max |
| Bicarbonate _ min | Bicarbonate _ avg | Creatinine _ min | Creatinine _ max | Chloride _ avg | Chloride _ avg |
| Chloride _ max | Creatinine _ avg | Urea nitrogen _ min | Creatinine _ avg | Creatinine _ max | Chloride _ min |
| Creatinine _ max | Creatinine _ min | Hematocrit _ max | Creatinine _ min | Creatinine _ avg | Creatinine _ avg |
| Creatinine _ avg | Urea nitrogen _ min | MCHC _ avg | Potassium _ max | Creatinine _ min | Creatinine _ min |
| Creatinine _ min | Hematocrit _ max | MCHC _ min | Sodium _ min | Glucose _ min | Glucose _ min |
| Glucose _ max | Hemoglobin _ max | PLT _ max | Urea nitrogen _ avg | Urea nitrogen _ max | Urea nitrogen _ max |
| Glucose _ avg | MCHC _ avg | PLT _ avg | Urea nitrogen _ min | Urea nitrogen _ avg | Urea nitrogen _ avg |
| Potassium _ max | MCHC _ min | PLT _ min | Hematocrit _ max | Urea nitrogen _ min | Urea nitrogen _ min |
| Potassium _ avg | PLT _ max | Magnesium _ max | MCH _ avg | PLT _ min | PLT _ max |
| Potassium _ min | PLT _ avg | Calcium _ avg | MCH _ min | RDW _ max | RDW _ max |
| Urea nitrogen _ max | PLT _ min | Calcium _ min | MCHC _ max | RDW _ avg | RDW _ avg |
| Urea nitrogen _ avg | RBC _ max | SOFA score | MCHC _ avg | RDW _ min | RDW _ min |
| Urea nitrogen _ min | WBC _ min | Heart rate on the first day of ICU _ avg | MCHC _ min | ALP _ max | TBil _ max |
| Hematocrit _ avg | ALP _ avg | SysBP on the first day of ICU _ max | PLT _ max | ALP _ avg | TBil _ avg |
| Hematocrit _ min | ALP _ min | SysBP on the first day of ICU _ avg | PLT _ avg | ALP _ min | TBil _ min |
| Hemoglobin _ avg | AST _avg | SysBP on the first day of ICU _ min | PLT _ min | Magnesium _ avg | ALP _ max |
| Hemoglobin _ min | AST _min | DiasBP on the first day of ICU _ max | TBil _ min | Phosphate _ avg | ALP _ avg |
| MCHC _ min | Magnesium _ max | DiasBP on the first day of ICU _ min | AST _avg | APSIII score | ALP _ min |
| MCV _ max | Calcium _ avg | Mean arterial pressure on the first day of ICU admission _ max | AST _min | SAPSII score | Magnesium _ avg |
| PLT _ max | SOFA score | Mean arterial pressure on the first day of ICU admission _ min | Magnesium _ max | Heart rate on the first day of ICU _ max | SAPSII score |
| PLT _ avg | Sepsis | Respiratory rate on the first day of ICU _ avg | Calcium _ avg | Heart rate on the first day of ICU _ avg | Heart rate on the first day of ICU _ max |
| PLT _ min | Heart rate on the first day of ICU _ avg | Temperature on the first day of ICU admission _ max | Calcium _ min | Heart rate on the first day of ICU _ min | Heart rate on the first day of ICU _ avg |
| RDW _ max | Heart rate on the first day of ICU _ min | Temperature on the first day of ICU admission _ avg | SOFA score | DiasBP on the first day of ICU _ max | Heart rate on the first day of ICU _ min |
| RDW _ avg | SysBP on the first day of ICU _ max | Temperature on the first day of ICU admission _ min | Heart rate on the first day of ICU _ max | Mean arterial pressure on the first day of ICU admission _ max | DiasBP on the first day of ICU _ max |
| RDW _ min | SysBP on the first day of ICU _ avg | SpO2 on the first day of ICU _ max | Heart rate on the first day of ICU _ avg | Respiratory rate on the first day of ICU _ avg | Respiratory rate on the first day of ICU _ max |
| RBC _ avg | SysBP on the first day of ICU _ min |  | Heart rate on the first day of ICU _ min | SpO2 on the first day of ICU _ avg | Respiratory rate on the first day of ICU _ avg |
| RBC _ min | DiasBP on the first day of ICU _ max |  | SysBP on the first day of ICU _ max |  | Temperature on the first day of ICU admission _ avg |
| WBC _ min | DiasBP on the first day of ICU _ min |  | SysBP on the first day of ICU _ avg |  | SpO2 on the first day of ICU _ avg |
| TBil _ max | Mean arterial pressure on the first day of ICU admission _ max |  | SysBP on the first day of ICU _ min |  |  |
| TBil _ avg | Mean arterial pressure on the first day of ICU admission _ min |  | DiasBP on the first day of ICU _ max |  |  |
| TBil _ min | Temperature on the first day of ICU admission _ max |  | DiasBP on the first day of ICU _ min |  |  |
| ALT _ min | Temperature on the first day of ICU admission _ min |  | Mean arterial pressure on the first day of ICU admission _ max |  |  |
| ALP _ max |  |  | Mean arterial pressure on the first day of ICU admission _ min |  |  |
| AST _max |  |  | Respiratory rate on the first day of ICU _ avg |  |  |
| AST _avg |  |  | Temperature on the first day of ICU admission _ max |  |  |
| AST _min |  |  | Temperature on the first day of ICU admission _ avg |  |  |
| Magnesium _ max |  |  | Temperature on the first day of ICU admission _ min |  |  |
| Magnesium _ avg |  |  | SpO2 on the first day of ICU _ max |  |  |
| Calcium _ min |  |  |  |  |  |
| Phosphate _ max |  |  |  |  |  |
| Phosphate _ avg |  |  |  |  |  |
| Phosphate _ min |  |  |  |  |  |
| APSIII score |  |  |  |  |  |
| GCS score |  |  |  |  |  |
| SAPSII score |  |  |  |  |  |
| OASIS score |  |  |  |  |  |
| SIRS score |  |  |  |  |  |
| SOFA score |  |  |  |  |  |
| Sepsis |  |  |  |  |  |
| AKI stage |  |  |  |  |  |
| Renal replacement therapy |  |  |  |  |  |
| Cultivate positivity |  |  |  |  |  |
| Heart rate on the first day of ICU _ max |  |  |  |  |  |
| Heart rate on the first day of ICU _ avg |  |  |  |  |  |
| SysBP on the first day of ICU _ max |  |  |  |  |  |
| SysBP on the first day of ICU _ avg |  |  |  |  |  |
| SysBP on the first day of ICU _ min |  |  |  |  |  |
| DiasBP on the first day of ICU _ max |  |  |  |  |  |
| DiasBP on the first day of ICU _ min |  |  |  |  |  |
| Mean arterial pressure on the first day of ICU admission _ max |  |  |  |  |  |
| Mean arterial pressure on the first day of ICU admission _ min |  |  |  |  |  |
| Respiratory rate on the first day of ICU _ max |  |  |  |  |  |
| Respiratory rate on the first day of ICU _ avg |  |  |  |  |  |
| Temperature on the first day of ICU admission _ max |  |  |  |  |  |
| Temperature on the first day of ICU admission _ avg |  |  |  |  |  |
| SpO2 on the first day of ICU _ max |  |  |  |  |  |
| SpO2 on the first day of ICU _ min |  |  |  |  |  |

ICU, intensive care unit; MCH, mean corpuscular hemoglobin; MCHC, mean corpuscular hemoglobin concentration; MCV, mean corpuscular volume; PLT, platelet count; RDW, red cell distribution width; RBC, red blood cells; WBC, white blood cells; TBil, total bilirubin; ALT, alanine aminotransferase; ALP, alkaline phosphatase; AST, aspartate aminotransferase; SIRS, systemic inflammatory response syndrome; SOFA, sepsis-related organ failure; AKI, acute kidney injury; SysBP, systolic blood pressure; DiasBP, diastolic blood pressure; SpO2, saturation of peripheral oxygen.

**Table S9. Summary of variables that were finally incorporated into each model**

| Patient categories | Outcomes | Variables ultimately incorporated into the models |
| --- | --- | --- |
| All patients | In-hospital mortality | Age Length of stay in hospital Bicarbonate _ avg Bicarbonate _ min Creatinine _ max Creatinine _ avg Glucose _ max Glucose _ avg Potassium _ avg Urea nitrogen _ max Urea nitrogen _ avg Hemoglobin _ min MCHC _ min MCV _ max PLT _ avg RDW _ max RDW _ avg WBC _ avg WBC _ min TBil _ min AST _min Magnesium _ max Calcium _ avg Phosphate _ max Phosphate _ avg |
|  |  |  |
|  |  |  |
|  |  |  |
|  |  |  |
|  |  |  |
|  |  |  |
|  |  |  |
|  |  |  |
|  |  |  |
|  |  |  |
|  |  |  |
|  |  |  |
|  |  |  |
|  |  |  |
|  |  |  |
|  |  |  |
|  |  |  |
|  |  |  |
|  |  |  |
|  |  |  |
|  |  |  |
|  |  |  |
|  |  |  |
|  |  |  |
|  | Re-admission within 30 days after discharge | Age Length of stay in hospital Surgical operations Endoscopy combined with surgical operations Endoscopy combined with interventional operations Anion gap _ avg Anion gap _ min Bicarbonate _ avg Bicarbonate _ min Chloride _ max Chloride _ avg Chloride _ min Creatinine _ avg Potassium _ min Sodium _ min MCH _ max MCHC _ avg PLT _ avg PLT _ min RDW _ avg TBil _ min ALP _ avg AST _avg AST _min Phosphate _ avg |
|  |  |  |
|  |  |  |
|  |  |  |
|  |  |  |
|  |  |  |
|  |  |  |
|  |  |  |
|  |  |  |
|  |  |  |
|  |  |  |
|  |  |  |
|  |  |  |
|  |  |  |
|  |  |  |
|  |  |  |
|  |  |  |
|  |  |  |
|  |  |  |
|  |  |  |
|  |  |  |
|  |  |  |
|  |  |  |
|  |  |  |
|  |  |  |
|  | Re-admission within 60 days after discharge | Age Length of stay in hospital Surgical operations Surgical or interventional or endoscopic operations Endoscopy combined with surgical operations Anion gap _ min Bicarbonate _ avg Bicarbonate _ min Chloride _ avg Creatinine _ avg Glucose _ max Potassium _ min Sodium _ min MCHC _ avg PLT _ avg TBil _ min AST _max AST _min Phosphate _ avg |
|  |  |  |
|  |  |  |
|  |  |  |
|  |  |  |
|  |  |  |
|  |  |  |
|  |  |  |
|  |  |  |
|  |  |  |
|  |  |  |
|  |  |  |
|  |  |  |
|  |  |  |
|  |  |  |
|  |  |  |
|  |  |  |
|  |  |  |
|  |  |  |
|  | Re-admission within 90 days after discharge | Age Gender Length of stay in hospital Surgical operations Surgical or interventional or endoscopic operations Endoscopy combined with surgical operations Anion gap _ min Bicarbonate _ min Chloride _ avg Creatinine _ max Creatinine _ avg Glucose _ avg Potassium _ avg Sodium _ min Urea nitrogen _ avg MCHC _ avg PLT _ avg PLT _ min TBil _ avg TBil _ min ALP _ avg AST _max AST _min Phosphate _ max Phosphate _ avg Phosphate _ min |
|  |  |  |
|  |  |  |
|  |  |  |
|  |  |  |
|  |  |  |
|  |  |  |
|  |  |  |
|  |  |  |
|  |  |  |
|  |  |  |
|  |  |  |
|  |  |  |
|  |  |  |
|  |  |  |
|  |  |  |
|  |  |  |
|  |  |  |
|  |  |  |
|  |  |  |
|  |  |  |
|  |  |  |
|  |  |  |
|  |  |  |
|  |  |  |
|  |  |  |
|  | Mortality within 180 days after discharge | Age Length of stay in hospital Surgical operations Anion gap _ avg Bicarbonate _ avg Potassium _ min Urea nitrogen _ max Urea nitrogen _ min Hemoglobin _ avg RDW _ min RBC _ min WBC _ max ALP _ avg ALP _ min |
|  |  |  |
|  |  |  |
|  |  |  |
|  |  |  |
|  |  |  |
|  |  |  |
|  |  |  |
|  |  |  |
|  |  |  |
|  |  |  |
|  |  |  |
|  |  |  |
|  |  |  |
|  | Mortality within 365 days after discharge | Age Length of stay in hospital Surgical operations Interventional operations Potassium _ min Urea nitrogen _ min RDW _ min RBC _ min WBC _ max TBil _ avg ALP _ avg ALP _ min |
|  |  |  |
|  |  |  |
|  |  |  |
|  |  |  |
|  |  |  |
|  |  |  |
|  |  |  |
|  |  |  |
|  |  |  |
|  |  |  |
|  |  |  |
| Patients underwent operations | In-hospital mortality | Age Length of stay in hospital Diabetes Bicarbonate _ min Glucose _ avg Potassium _ avg Sodium _ min WBC _ max TBil _ avg ALT _max AST _max AST _min Calcium _ avg Calcium _ min Phosphate _ max Phosphate _ avg |
|  |  |  |
|  |  |  |
|  |  |  |
|  |  |  |
|  |  |  |
|  |  |  |
|  |  |  |
|  |  |  |
|  |  |  |
|  |  |  |
|  |  |  |
|  |  |  |
|  |  |  |
|  |  |  |
|  |  |  |
|  | Re-admission within 30 days after discharge | Length of stay in hospital Length of stay in ICU Endoscopy combined with surgical operations Chloride _ avg Chloride _ min Glucose _ max Glucose _ avg MCHC _ avg MCV _ max PLT _ avg PLT _ min AST _avg Magnesium _ max |
|  |  |  |
|  |  |  |
|  |  |  |
|  |  |  |
|  |  |  |
|  |  |  |
|  |  |  |
|  |  |  |
|  |  |  |
|  |  |  |
|  |  |  |
|  |  |  |
|  | Re-admission within 60 days after discharge | Age Length of stay in hospital Surgical operations Endoscopy combined with surgical operations Anion gap _ min Chloride _ avg Glucose _ max Glucose _ avg Potassium _ avg MCHC _ avg MCV _ max PLT _ avg PLT _ min Magnesium _ max Calcium _ avg |
|  |  |  |
|  |  |  |
|  |  |  |
|  |  |  |
|  |  |  |
|  |  |  |
|  |  |  |
|  |  |  |
|  |  |  |
|  |  |  |
|  |  |  |
|  |  |  |
|  |  |  |
|  |  |  |
|  | Re-admission within 90 days after discharge | Age Length of stay in hospital Surgical operations Endoscopy combined with surgical operations Anion gap _ min Chloride _ avg Glucose _ avg Potassium _ avg MCHC _ avg MCV _ max PLT _ min RBC _ avg AST _avg Magnesium _ max Calcium _ avg |
|  |  |  |
|  |  |  |
|  |  |  |
|  |  |  |
|  |  |  |
|  |  |  |
|  |  |  |
|  |  |  |
|  |  |  |
|  |  |  |
|  |  |  |
|  |  |  |
|  |  |  |
|  |  |  |
|  | Mortality within 180 days after discharge | Age Length of stay in hospital Surgical operations Hypertension Diabetes Potassium _ min Urea nitrogen _ max Urea nitrogen _ min MCHC _ avg RDW _ min RBC _ avg ALP _ min AST _avg |
|  |  |  |
|  |  |  |
|  |  |  |
|  |  |  |
|  |  |  |
|  |  |  |
|  |  |  |
|  |  |  |
|  |  |  |
|  |  |  |
|  |  |  |
|  |  |  |
|  | Mortality within 365 days after discharge | Age Gender Length of stay in hospital Surgical operations Diabetes Chloride _ min Potassium _ min Sodium _ min Urea nitrogen _ min MCV _ avg RDW _ min RBC _ avg ALP _ min |
|  |  |  |
|  |  |  |
|  |  |  |
|  |  |  |
|  |  |  |
|  |  |  |
|  |  |  |
|  |  |  |
|  |  |  |
|  |  |  |
|  |  |  |
|  |  |  |
| Patients admitted to the ICU | In-hospital mortality | Age Bicarbonate _ avg Creatinine _ max Glucose _ avg Potassium _ avg Urea nitrogen _ avg Hemoglobin _ min MCHC _ min MCV _ max PLT _ max PLT _ avg RDW _ avg WBC _ min TBil _ min AST _max AST _min Phosphate _ max Phosphate _ avg Sepsis AKI Heart rate on the first day of ICU _ max Heart rate on the first day of ICU _ avg SysBP on the first day of ICU _ max SysBP on the first day of ICU _ min Respiratory rate on the first day of ICU _ max SpO2 on the first day of ICU _ max SpO2 on the first day of ICU _ min |
|  |  |  |
|  |  |  |
|  |  |  |
|  |  |  |
|  |  |  |
|  |  |  |
|  |  |  |
|  |  |  |
|  |  |  |
|  |  |  |
|  |  |  |
|  |  |  |
|  |  |  |
|  |  |  |
|  |  |  |
|  |  |  |
|  |  |  |
|  |  |  |
|  |  |  |
|  |  |  |
|  |  |  |
|  |  |  |
|  |  |  |
|  |  |  |
|  |  |  |
|  |  |  |
|  | Re-admission within 30 days after discharge | Length of stay in hospital Hypertension Anion gap _ max Anion gap _ min Creatinine _ min Hemoglobin _ max MCHC _ avg PLT _ avg WBC _ min ALP _ avg Magnesium _ max Calcium _ avg Sepsis Heart rate on the first day of ICU _ avg SysBP on the first day of ICU _ max Mean arterial pressure on the first day of ICU admission _ min |
|  |  |  |
|  |  |  |
|  |  |  |
|  |  |  |
|  |  |  |
|  |  |  |
|  |  |  |
|  |  |  |
|  |  |  |
|  |  |  |
|  |  |  |
|  |  |  |
|  |  |  |
|  |  |  |
|  |  |  |
|  | Re-admission within 60 days after discharge | Anion gap _ min Creatinine _ min Urea nitrogen _ min Hematocrit _ max MCHC _ avg PLT _ max PLT _ avg Magnesium _ max Calcium _ avg SysBP on the first day of ICU _ avg Mean arterial pressure on the first day of ICU admission _ min Respiratory rate on the first day of ICU _ avg Temperature on the first day of ICU admission _ max Temperature on the first day of ICU admission _ avg SpO2 on the first day of ICU _ max |
|  |  |  |
|  |  |  |
|  |  |  |
|  |  |  |
|  |  |  |
|  |  |  |
|  |  |  |
|  |  |  |
|  |  |  |
|  |  |  |
|  |  |  |
|  |  |  |
|  |  |  |
|  |  |  |
|  | Re-admission within 90 days after discharge | Anion gap _ max Anion gap _ min Chloride _ min Creatinine _ min Sodium _ min Urea nitrogen _ min Hematocrit _ max MCH _ avg MCHC _ avg PLT _ avg Magnesium _ max Calcium _ avg SOFA score SysBP on the first day of ICU _ avg SysBP on the first day of ICU _ min Mean arterial pressure on the first day of ICU admission _ min Respiratory rate on the first day of ICU _ avg Temperature on the first day of ICU admission _ max SpO2 on the first day of ICU _ max |
|  |  |  |
|  |  |  |
|  |  |  |
|  |  |  |
|  |  |  |
|  |  |  |
|  |  |  |
|  |  |  |
|  |  |  |
|  |  |  |
|  |  |  |
|  |  |  |
|  |  |  |
|  |  |  |
|  |  |  |
|  |  |  |
|  |  |  |
|  |  |  |
|  | Mortality within 180 days after discharge | Age Bicarbonate _ avg Chloride _ max Creatinine _ max Glucose _ min Urea nitrogen _ min PLT _ min RDW _ min ALP _ max ALP _ min Heart rate on the first day of ICU _ avg Respiratory rate on the first day of ICU _ avg SpO2 on the first day of ICU _ avg |
|  |  |  |
|  |  |  |
|  |  |  |
|  |  |  |
|  |  |  |
|  |  |  |
|  |  |  |
|  |  |  |
|  |  |  |
|  |  |  |
|  |  |  |
|  |  |  |
|  | Mortality within 365 days after discharge | Age Bicarbonate _ avg Chloride _ max Glucose _ min Urea nitrogen _ min RDW _ min TBil _ avg ALP _ max Heart rate on the first day of ICU _ avg Respiratory rate on the first day of ICU _ avg SpO2 on the first day of ICU _ avg |
|  |  |  |
|  |  |  |
|  |  |  |
|  |  |  |
|  |  |  |
|  |  |  |
|  |  |  |
|  |  |  |
|  |  |  |
|  |  |  |

ICU, intensive care unit; MCH, mean corpuscular hemoglobin; MCHC, mean corpuscular hemoglobin concentration; MCV, mean corpuscular volume; PLT, platelet count; RDW, red cell distribution width; RBC, red blood cells; WBC, white blood cells; TBil, total bilirubin; ALT, alanine aminotransferase; ALP, alkaline phosphatase; AST, aspartate aminotransferase; SIRS, systemic inflammatory response syndrome; SOFA, sepsis-related organ failure; AKI, acute kidney injury; SysBP, systolic blood pressure; DiasBP, diastolic blood pressure; SpO2, saturation of peripheral oxygen.

**Table S10. Performance of machine learning algorithms (Predicting in-hospital mortality for all the patients included)**

| Class | Model | AUC (95%CI) | Accuracy | Sensitivity | Specificity | PPV | NPV | F1 score |
| --- | --- | --- | --- | --- | --- | --- | --- | --- |
| Training set | KNN | 1.000 (1.000−1.000) | 1.000 | 1.000 | 1.000 | 1.000 | 1.000 | 1.000 |
| Training set | LGBM | 1.000 (1.000−1.000) | 1.000 | 1.000 | 1.000 | 1.000 | 1.000 | 1.000 |
| Training set | LR | 0.977 (0.967−0.988) | 0.923 | 0.966 | 0.880 | 0.212 | 0.999 | 0.348 |
| Training set | RF | 1.000 (1.000−1.000) | 1.000 | 1.000 | 1.000 | 1.000 | 1.000 | 1.000 |
| Training set | SVM | 0.963 (0.941−0.985) | 0.900 | 0.845 | 0.956 | 0.392 | 0.995 | 0.536 |
| Training set | XGB | 0.995 (0.993−0.998) | 0.977 | 1.000 | 0.955 | 0.426 | 1.000 | 0.598 |
| Validation set | KNN | 0.824 (0.726−0.922) | 0.744 | 0.500 | 0.988 | 0.571 | 0.984 | 0.533 |
| Validation set | LGBM | 0.951 (0.907−0.995) | 0.661 | 0.333 | 0.989 | 0.500 | 0.979 | 0.400 |
| Validation set | LR | 0.957 (0.917−0.997) | 0.898 | 0.917 | 0.879 | 0.196 | 0.997 | 0.324 |
| Validation set | RF | 0.966 (0.937−0.995) | 0.747 | 0.500 | 0.995 | 0.750 | 0.984 | 0.600 |
| Validation set | SVM | 0.917 (0.841−0.992) | 0.873 | 0.792 | 0.954 | 0.358 | 0.993 | 0.494 |
| Validation set | XGB | 0.957 (0.918−0.997) | 0.894 | 0.833 | 0.956 | 0.377 | 0.994 | 0.519 |

AUC, area under the curve; CI, confidence interval; PPV, pos pred value; NPV, neg pred value; KNN, K-Nearest Neighbor; LGBM, light gradient boosting machine; LR, logistic regression; RF, random forest; SVM, support vector machine; XGB, eXtreme gradient boosting.

**Table S11. Performance of machine learning algorithms (Predicting re-admission within 30 days after discharge for all the patients included)**

| Class | Model | AUC (95%CI) | Accuracy | Sensitivity | Specificity | PPV | NPV | F1 score |
| --- | --- | --- | --- | --- | --- | --- | --- | --- |
| Training set | KNN | 1.000 (1.000−1.000) | 1.000 | 1.000 | 1.000 | 1.000 | 1.000 | 1.000 |
| Training set | LGBM | 0.964 (0.950−0.977) | 0.899 | 0.872 | 0.926 | 0.529 | 0.987 | 0.659 |
| Training set | LR | 0.713 (0.671−0.755) | 0.666 | 0.615 | 0.717 | 0.172 | 0.951 | 0.269 |
| Training set | RF | 1.000 (0.999−1.000) | 0.996 | 0.994 | 0.998 | 0.981 | 0.999 | 0.987 |
| Training set | SVM | 0.570 (0.525−0.616) | 0.441 | 0.186 | 0.697 | 0.055 | 0.900 | 0.085 |
| Training set | XGB | 0.777 (0.737−0.816) | 0.717 | 0.776 | 0.657 | 0.178 | 0.968 | 0.289 |
| Validation set | KNN | 0.533 (0.473−0.592) | 0.520 | 0.136 | 0.905 | 0.118 | 0.918 | 0.127 |
| Validation set | LGBM | 0.531 (0.455−0.607) | 0.521 | 0.167 | 0.875 | 0.111 | 0.918 | 0.133 |
| Validation set | LR | 0.592 (0.513−0.672) | 0.559 | 0.439 | 0.679 | 0.114 | 0.928 | 0.181 |
| Validation set | RF | 0.531 (0.447−0.615) | 0.571 | 0.227 | 0.915 | 0.200 | 0.926 | 0.213 |
| Validation set | SVM | 0.538 (0.465−0.612) | 0.527 | 0.318 | 0.736 | 0.102 | 0.920 | 0.154 |
| Validation set | XGB | 0.568 (0.489−0.647) | 0.568 | 0.530 | 0.605 | 0.112 | 0.932 | 0.185 |

AUC, area under the curve; CI, confidence interval; PPV, pos pred value; NPV, neg pred value; KNN, K-Nearest Neighbor; LGBM, light gradient boosting machine; LR, logistic regression; RF, random forest; SVM, support vector machine; XGB, eXtreme gradient boosting.

**Table S12. Performance of machine learning algorithms (Predicting re-admission within 60 days after discharge for all the patients included)**

| Class | Model | AUC (95%CI) | Accuracy | Sensitivity | Specificity | PPV | NPV | F1 score |
| --- | --- | --- | --- | --- | --- | --- | --- | --- |
| Training set | KNN | 1.000 (1.000−1.000) | 1.000 | 1.000 | 1.000 | 1.000 | 1.000 | 1.000 |
| Training set | LGBM | 0.932 (0.917−0.947) | 0.859 | 0.837 | 0.880 | 0.472 | 0.977 | 0.604 |
| Training set | LR | 0.710 (0.674−0.747) | 0.656 | 0.650 | 0.662 | 0.198 | 0.937 | 0.303 |
| Training set | RF | 1.000 (1.000−1.000) | 0.996 | 0.995 | 0.997 | 0.981 | 0.999 | 0.988 |
| Training set | SVM | 0.511 (0.470−0.552) | 0.528 | 0.670 | 0.386 | 0.122 | 0.901 | 0.207 |
| Training set | XGB | 0.764 (0.730−0.797) | 0.698 | 0.764 | 0.632 | 0.209 | 0.954 | 0.329 |
| Validation set | KNN | 0.547 (0.491−0.603) | 0.502 | 0.129 | 0.874 | 0.113 | 0.890 | 0.121 |
| Validation set | LGBM | 0.576 (0.511−0.640) | 0.557 | 0.318 | 0.796 | 0.163 | 0.904 | 0.215 |
| Validation set | LR | 0.633 (0.566−0.700) | 0.593 | 0.565 | 0.621 | 0.156 | 0.920 | 0.245 |
| Validation set | RF | 0.623 (0.557−0.689) | 0.550 | 0.176 | 0.924 | 0.224 | 0.900 | 0.197 |
| Validation set | SVM | 0.554 (0.495−0.614) | 0.480 | 0.612 | 0.348 | 0.105 | 0.878 | 0.179 |
| Validation set | XGB | 0.653 (0.587−0.718) | 0.609 | 0.612 | 0.606 | 0.162 | 0.926 | 0.256 |

AUC, area under the curve; CI, confidence interval; PPV, pos pred value; NPV, neg pred value; KNN, K-Nearest Neighbor; LGBM, light gradient boosting machine; LR, logistic regression; RF, random forest; SVM, support vector machine; XGB, eXtreme gradient boosting.

**Table S13. Performance of machine learning algorithms (Predicting re-admission within 90 days after discharge for all the patients included)**

| Class | Model | AUC (95%CI) | Accuracy | Sensitivity | Specificity | PPV | NPV | F1 score |
| --- | --- | --- | --- | --- | --- | --- | --- | --- |
| Training set | KNN | 1.000 (1.000−1.000) | 1.000 | 1.000 | 1.000 | 1.000 | 1.000 | 1.000 |
| Training set | LGBM | 0.957 (0.945−0.969) | 0.898 | 0.912 | 0.884 | 0.531 | 0.986 | 0.671 |
| Training set | LR | 0.708 (0.672−0.743) | 0.664 | 0.650 | 0.677 | 0.225 | 0.931 | 0.334 |
| Training set | RF | 0.999 (0.998−1.000) | 0.997 | 0.996 | 0.997 | 0.983 | 0.999 | 0.989 |
| Training set | SVM | 0.535 (0.497−0.572) | 0.549 | 0.717 | 0.382 | 0.143 | 0.903 | 0.239 |
| Training set | XGB | 0.828 (0.799−0.858) | 0.764 | 0.712 | 0.815 | 0.358 | 0.952 | 0.476 |
| Validation set | KNN | 0.508 (0.455−0.561) | 0.517 | 0.156 | 0.878 | 0.155 | 0.879 | 0.155 |
| Validation set | LGBM | 0.601 (0.538−0.663) | 0.567 | 0.323 | 0.811 | 0.196 | 0.893 | 0.244 |
| Validation set | LR | 0.655 (0.593−0.717) | 0.609 | 0.562 | 0.655 | 0.189 | 0.913 | 0.283 |
| Validation set | RF | 0.625 (0.558−0.692) | 0.628 | 0.385 | 0.871 | 0.298 | 0.908 | 0.336 |
| Validation set | SVM | 0.534 (0.472−0.595) | 0.509 | 0.656 | 0.362 | 0.128 | 0.880 | 0.214 |
| Validation set | XGB | 0.664 (0.602−0.727) | 0.609 | 0.448 | 0.769 | 0.217 | 0.907 | 0.293 |

AUC, area under the curve; CI, confidence interval; PPV, pos pred value; NPV, neg pred value; KNN, K-Nearest Neighbor; LGBM, light gradient boosting machine; LR, logistic regression; RF, random forest; SVM, support vector machine; XGB, eXtreme gradient boosting.

**Table S14. Performance of machine learning algorithms (Predicting mortality within 180 days after discharge for all the patients included)**

| Class | Model | AUC (95%CI) | Accuracy | Sensitivity | Specificity | PPV | NPV | F1 score |
| --- | --- | --- | --- | --- | --- | --- | --- | --- |
| Training set | KNN | 1.000 (1.000−1.000) | 1.000 | 1.000 | 1.000 | 1.000 | 1.000 | 1.000 |
| Training set | LGBM | 0.988 (0.982−0.994) | 0.956 | 0.988 | 0.925 | 0.380 | 0.999 | 0.549 |
| Training set | LR | 0.857 (0.821−0.893) | 0.784 | 0.800 | 0.767 | 0.139 | 0.988 | 0.236 |
| Training set | RF | 1.000 (1.000−1.000) | 1.000 | 1.000 | 1.000 | 1.000 | 1.000 | 1.000 |
| Training set | SVM | 0.706 (0.646−0.767) | 0.304 | 0.325 | 0.283 | 0.021 | 0.900 | 0.039 |
| Training set | XGB | 0.913 (0.886−0.940) | 0.842 | 0.863 | 0.822 | 0.185 | 0.992 | 0.305 |
| Validation set | KNN | 0.675 (0.586−0.763) | 0.502 | 0.034 | 0.970 | 0.043 | 0.962 | 0.038 |
| Validation set | LGBM | 0.751 (0.664−0.837) | 0.617 | 0.310 | 0.924 | 0.138 | 0.972 | 0.191 |
| Validation set | LR | 0.843 (0.785−0.902) | 0.782 | 0.793 | 0.771 | 0.120 | 0.990 | 0.208 |
| Validation set | RF | 0.839 (0.767−0.912) | 0.515 | 0.034 | 0.995 | 0.200 | 0.963 | 0.059 |
| Validation set | SVM | 0.713 (0.618−0.807) | 0.277 | 0.276 | 0.279 | 0.015 | 0.907 | 0.028 |
| Validation set | XGB | 0.844 (0.787−0.900) | 0.717 | 0.621 | 0.813 | 0.115 | 0.982 | 0.195 |

AUC, area under the curve; CI, confidence interval; PPV, pos pred value; NPV, neg pred value; KNN, K-Nearest Neighbor; LGBM, light gradient boosting machine; LR, logistic regression; RF, random forest; SVM, support vector machine; XGB, eXtreme gradient boosting.

**Table S15. Performance of machine learning algorithms (Predicting mortality within 365 days after discharge for all the patients included)**

| Class | Model | AUC (95%CI) | Accuracy | Sensitivity | Specificity | PPV | NPV | F1 score |
| --- | --- | --- | --- | --- | --- | --- | --- | --- |
| Training set | KNN | 1.000 (1.000−1.000) | 1.000 | 1.000 | 1.000 | 1.000 | 1.000 | 1.000 |
| Training set | LGBM | 0.962 (0.951−0.974) | 0.911 | 0.939 | 0.884 | 0.356 | 0.995 | 0.517 |
| Training set | LR | 0.827 (0.792−0.863) | 0.756 | 0.713 | 0.799 | 0.196 | 0.976 | 0.307 |
| Training set | RF | 1.000 (1.000−1.000) | 1.000 | 1.000 | 1.000 | 1.000 | 1.000 | 1.000 |
| Training set | SVM | 0.672 (0.618−0.726) | 0.351 | 0.409 | 0.293 | 0.038 | 0.878 | 0.070 |
| Training set | XGB | 0.888 (0.860−0.916) | 0.820 | 0.791 | 0.848 | 0.263 | 0.983 | 0.395 |
| Validation set | KNN | 0.561 (0.490−0.631) | 0.514 | 0.077 | 0.952 | 0.079 | 0.951 | 0.078 |
| Validation set | LGBM | 0.744 (0.660−0.828) | 0.638 | 0.410 | 0.866 | 0.140 | 0.965 | 0.209 |
| Validation set | LR | 0.804 (0.730−0.877) | 0.758 | 0.718 | 0.798 | 0.160 | 0.981 | 0.262 |
| Validation set | RF | 0.819 (0.749−0.890) | 0.533 | 0.077 | 0.989 | 0.273 | 0.952 | 0.120 |
| Validation set | SVM | 0.673 (0.586−0.760) | 0.340 | 0.385 | 0.295 | 0.028 | 0.900 | 0.053 |
| Validation set | XGB | 0.820 (0.752−0.887) | 0.743 | 0.641 | 0.845 | 0.181 | 0.978 | 0.282 |

AUC, area under the curve; CI, confidence interval; PPV, pos pred value; NPV, neg pred value; KNN, K-Nearest Neighbor; LGBM, light gradient boosting machine; LR, logistic regression; RF, random forest; SVM, support vector machine; XGB, eXtreme gradient boosting.

**Table S16. Performance of machine learning algorithms (Predicting in-hospital mortality for patients underwent operations)**

| Class | Model | AUC (95%CI) | Accuracy | Sensitivity | Specificity | PPV | NPV | F1 score |
| --- | --- | --- | --- | --- | --- | --- | --- | --- |
| Training set | KNN | 1.000 (1.000−1.000) | 1.000 | 1.000 | 1.000 | 1.000 | 1.000 | 1.000 |
| Training set | LGBM | 1.000 (1.000−1.000) | 1.000 | 1.000 | 1.000 | 1.000 | 1.000 | 1.000 |
| Training set | LR | 0.991 (0.984−0.998) | 0.981 | 1.000 | 0.961 | 0.408 | 1.000 | 0.580 |
| Training set | RF | 1.000 (1.000−1.000) | 1.000 | 1.000 | 1.000 | 1.000 | 1.000 | 1.000 |
| Training set | SVM | 0.975 (0.945−1.000) | 0.932 | 0.950 | 0.913 | 0.226 | 0.999 | 0.365 |
| Training set | XGB | 0.999 (0.999−1.000) | 0.998 | 1.000 | 0.996 | 0.870 | 1.000 | 0.930 |
| Validation set | KNN | 0.823 (0.615−1.000) | 0.744 | 0.500 | 0.987 | 0.429 | 0.991 | 0.462 |
| Validation set | LGBM | 0.868 (0.690−1.000) | 0.748 | 0.500 | 0.997 | 0.750 | 0.991 | 0.600 |
| Validation set | LR | 0.883 (0.679−1.000) | 0.808 | 0.667 | 0.950 | 0.200 | 0.993 | 0.308 |
| Validation set | RF | 0.926 (0.844−1.000) | 0.583 | 0.167 | 1.000 | 1.000 | 0.984 | 0.286 |
| Validation set | SVM | 0.893 (0.715−1.000) | 0.861 | 0.833 | 0.890 | 0.125 | 0.996 | 0.217 |
| Validation set | XGB | 0.930 (0.858−1.000) | 0.662 | 0.333 | 0.991 | 0.400 | 0.987 | 0.364 |

AUC, area under the curve; CI, confidence interval; PPV, pos pred value; NPV, neg pred value; KNN, K-Nearest Neighbor; LGBM, light gradient boosting machine; LR, logistic regression; RF, random forest; SVM, support vector machine; XGB, eXtreme gradient boosting.

**Table S17. Performance of machine learning algorithms (Predicting re-admission within 30 days after discharge for patients underwent operations)**

| Class | Model | AUC (95%CI) | Accuracy | Sensitivity | Specificity | PPV | NPV | F1 score |
| --- | --- | --- | --- | --- | --- | --- | --- | --- |
| Training set | KNN | 1.000 (1.000−1.000) | 1.000 | 1.000 | 1.000 | 1.000 | 1.000 | 1.000 |
| Training set | LGBM | 0.888 (0.838−0.938) | 0.844 | 0.824 | 0.865 | 0.302 | 0.986 | 0.442 |
| Training set | LR | 0.721 (0.645−0.797) | 0.663 | 0.647 | 0.679 | 0.125 | 0.964 | 0.210 |
| Training set | RF | 1.000 (1.000−1.000) | 1.000 | 1.000 | 1.000 | 1.000 | 1.000 | 1.000 |
| Training set | SVM | 0.578 (0.495−0.660) | 0.581 | 0.686 | 0.476 | 0.085 | 0.955 | 0.152 |
| Training set | XGB | 0.790 (0.722−0.857) | 0.719 | 0.804 | 0.635 | 0.135 | 0.978 | 0.232 |
| Validation set | KNN | 0.531 (0.453−0.608) | 0.522 | 0.111 | 0.932 | 0.130 | 0.920 | 0.120 |
| Validation set | LGBM | 0.635 (0.511−0.759) | 0.621 | 0.407 | 0.834 | 0.183 | 0.939 | 0.253 |
| Validation set | LR | 0.608 (0.487−0.728) | 0.584 | 0.481 | 0.686 | 0.123 | 0.935 | 0.195 |
| Validation set | RF | 0.567 (0.444−0.690) | 0.512 | 0.037 | 0.986 | 0.200 | 0.918 | 0.062 |
| Validation set | SVM | 0.572 (0.465−0.678) | 0.563 | 0.593 | 0.534 | 0.104 | 0.935 | 0.177 |
| Validation set | XGB | 0.625 (0.501−0.749) | 0.550 | 0.481 | 0.618 | 0.103 | 0.929 | 0.170 |

AUC, area under the curve; CI, confidence interval; PPV, pos pred value; NPV, neg pred value; KNN, K-Nearest Neighbor; LGBM, light gradient boosting machine; LR, logistic regression; RF, random forest; SVM, support vector machine; XGB, eXtreme gradient boosting.

**Table S18. Performance of machine learning algorithms (Predicting re-admission within 60 days after discharge for patients underwent operations)**

| Class | Model | AUC (95%CI) | Accuracy | Sensitivity | Specificity | PPV | NPV | F1 score |
| --- | --- | --- | --- | --- | --- | --- | --- | --- |
| Training set | KNN | 1.000 (1.000−1.000) | 1.000 | 1.000 | 1.000 | 1.000 | 1.000 | 1.000 |
| Training set | LGBM | 0.912 (0.876−0.948) | 0.857 | 0.918 | 0.797 | 0.322 | 0.989 | 0.477 |
| Training set | LR | 0.755 (0.693−0.817) | 0.700 | 0.699 | 0.702 | 0.198 | 0.957 | 0.308 |
| Training set | RF | 1.000 (1.000−1.000) | 1.000 | 1.000 | 1.000 | 1.000 | 1.000 | 1.000 |
| Training set | SVM | 0.652 (0.580−0.723) | 0.626 | 0.452 | 0.800 | 0.192 | 0.933 | 0.269 |
| Training set | XGB | 0.850 (0.802−0.899) | 0.783 | 0.822 | 0.744 | 0.252 | 0.975 | 0.386 |
| Validation set | KNN | 0.624 (0.534−0.714) | 0.502 | 0.062 | 0.942 | 0.105 | 0.901 | 0.078 |
| Validation set | LGBM | 0.632 (0.523−0.741) | 0.598 | 0.438 | 0.759 | 0.167 | 0.925 | 0.241 |
| Validation set | LR | 0.589 (0.484−0.694) | 0.569 | 0.469 | 0.670 | 0.135 | 0.920 | 0.210 |
| Validation set | RF | 0.619 (0.517−0.722) | 0.519 | 0.062 | 0.976 | 0.222 | 0.904 | 0.098 |
| Validation set | SVM | 0.597 (0.488−0.705) | 0.527 | 0.250 | 0.804 | 0.123 | 0.907 | 0.165 |
| Validation set | XGB | 0.667 (0.573−0.760) | 0.580 | 0.469 | 0.691 | 0.143 | 0.922 | 0.219 |

AUC, area under the curve; CI, confidence interval; PPV, pos pred value; NPV, neg pred value; KNN, K-Nearest Neighbor; LGBM, light gradient boosting machine; LR, logistic regression; RF, random forest; SVM, support vector machine; XGB, eXtreme gradient boosting.

**Table S19. Performance of machine learning algorithms (Predicting re-admission within 90 days after discharge for patients underwent operations)**

| Class | Model | AUC (95%CI) | Accuracy | Sensitivity | Specificity | PPV | NPV | F1 score |
| --- | --- | --- | --- | --- | --- | --- | --- | --- |
| Training set | KNN | 1.000 (1.000−1.000) | 1.000 | 1.000 | 1.000 | 1.000 | 1.000 | 1.000 |
| Training set | LGBM | 0.918 (0.887−0.949) | 0.856 | 0.899 | 0.814 | 0.357 | 0.986 | 0.511 |
| Training set | LR | 0.754 (0.695−0.812) | 0.691 | 0.709 | 0.673 | 0.199 | 0.953 | 0.311 |
| Training set | RF | 1.000 (1.000−1.000) | 1.000 | 1.000 | 1.000 | 1.000 | 1.000 | 1.000 |
| Training set | SVM | 0.643 (0.580−0.707) | 0.622 | 0.772 | 0.472 | 0.144 | 0.948 | 0.242 |
| Training set | XGB | 0.846 (0.798−0.894) | 0.779 | 0.734 | 0.824 | 0.324 | 0.964 | 0.450 |
| Validation set | KNN | 0.606 (0.515−0.698) | 0.565 | 0.200 | 0.931 | 0.259 | 0.905 | 0.226 |
| Validation set | LGBM | 0.589 (0.496−0.682) | 0.538 | 0.343 | 0.733 | 0.135 | 0.902 | 0.194 |
| Validation set | LR | 0.612 (0.514−0.710) | 0.592 | 0.514 | 0.670 | 0.159 | 0.919 | 0.243 |
| Validation set | RF | 0.601 (0.507−0.694) | 0.518 | 0.057 | 0.979 | 0.250 | 0.895 | 0.093 |
| Validation set | SVM | 0.537 (0.435−0.638) | 0.553 | 0.571 | 0.535 | 0.130 | 0.911 | 0.212 |
| Validation set | XGB | 0.654 (0.555−0.753) | 0.587 | 0.371 | 0.802 | 0.186 | 0.913 | 0.248 |

AUC, area under the curve; CI, confidence interval; PPV, pos pred value; NPV, neg pred value; KNN, K-Nearest Neighbor; LGBM, light gradient boosting machine; LR, logistic regression; RF, random forest; SVM, support vector machine; XGB, eXtreme gradient boosting.

**Table S20. Performance of machine learning algorithms (Predicting mortality within 180 days after discharge for patients underwent operations)**

| Class | Model | AUC (95%CI) | Accuracy | Sensitivity | Specificity | PPV | NPV | F1 score |
| --- | --- | --- | --- | --- | --- | --- | --- | --- |
| Training set | KNN | 1.000 (1.000−1.000) | 1.000 | 1.000 | 1.000 | 1.000 | 1.000 | 1.000 |
| Training set | LGBM | 0.969 (0.953−0.986) | 0.915 | 0.949 | 0.882 | 0.301 | 0.997 | 0.457 |
| Training set | LR | 0.868 (0.817−0.920) | 0.820 | 0.821 | 0.820 | 0.196 | 0.988 | 0.317 |
| Training set | RF | 1.000 (1.000−1.000) | 1.000 | 1.000 | 1.000 | 1.000 | 1.000 | 1.000 |
| Training set | SVM | 0.726 (0.648−0.804) | 0.687 | 0.795 | 0.579 | 0.092 | 0.981 | 0.164 |
| Training set | XGB | 0.932 (0.896−0.968) | 0.882 | 0.872 | 0.892 | 0.301 | 0.992 | 0.447 |
| Validation set | KNN | 0.590 (0.467−0.713) | 0.518 | 0.071 | 0.964 | 0.083 | 0.958 | 0.077 |
| Validation set | LGBM | 0.835 (0.733−0.937) | 0.763 | 0.643 | 0.883 | 0.200 | 0.982 | 0.305 |
| Validation set | LR | 0.850 (0.783−0.917) | 0.788 | 0.786 | 0.790 | 0.145 | 0.988 | 0.244 |
| Validation set | RF | 0.845 (0.755−0.936) | 0.495 | 0.000 | 0.990 | 0.000 | 0.956 | NaN |
| Validation set | SVM | 0.631 (0.490−0.772) | 0.650 | 0.714 | 0.586 | 0.072 | 0.978 | 0.132 |
| Validation set | XGB | 0.831 (0.733−0.928) | 0.697 | 0.500 | 0.893 | 0.175 | 0.975 | 0.259 |

AUC, area under the curve; CI, confidence interval; PPV, pos pred value; NPV, neg pred value; KNN, K-Nearest Neighbor; LGBM, light gradient boosting machine; LR, logistic regression; RF, random forest; SVM, support vector machine; XGB, eXtreme gradient boosting.

**Table S21. Performance of machine learning algorithms (Predicting mortality within 365 days after discharge for patients underwent operations)**

| Class | Model | AUC (95%CI) | Accuracy | Sensitivity | Specificity | PPV | NPV | F1 score |
| --- | --- | --- | --- | --- | --- | --- | --- | --- |
| Training set | KNN | 1.000 (1.000−1.000) | 1.000 | 1.000 | 1.000 | 1.000 | 1.000 | 1.000 |
| Training set | LGBM | 0.926 (0.896−0.957) | 0.866 | 0.953 | 0.780 | 0.282 | 0.995 | 0.436 |
| Training set | LR | 0.852 (0.812−0.892) | 0.792 | 0.859 | 0.724 | 0.221 | 0.983 | 0.351 |
| Training set | RF | 1.000 (1.000−1.000) | 1.000 | 1.000 | 1.000 | 1.000 | 1.000 | 1.000 |
| Training set | SVM | 0.736 (0.679−0.792) | 0.707 | 0.875 | 0.538 | 0.147 | 0.979 | 0.252 |
| Training set | XGB | 0.964 (0.947−0.982) | 0.919 | 0.938 | 0.901 | 0.462 | 0.994 | 0.619 |
| Validation set | KNN | 0.707 (0.586−0.828) | 0.562 | 0.167 | 0.957 | 0.187 | 0.951 | 0.176 |
| Validation set | LGBM | 0.803 (0.719−0.886) | 0.656 | 0.556 | 0.757 | 0.119 | 0.967 | 0.196 |
| Validation set | LR | 0.896 (0.842−0.949) | 0.810 | 0.889 | 0.731 | 0.163 | 0.991 | 0.276 |
| Validation set | RF | 0.904 (0.847−0.960) | 0.551 | 0.111 | 0.990 | 0.400 | 0.950 | 0.174 |
| Validation set | SVM | 0.713 (0.581−0.845) | 0.666 | 0.833 | 0.498 | 0.089 | 0.981 | 0.161 |
| Validation set | XGB | 0.881 (0.812−0.950) | 0.740 | 0.611 | 0.869 | 0.216 | 0.974 | 0.319 |

AUC, area under the curve; CI, confidence interval; PPV, pos pred value; NPV, neg pred value; KNN, K-Nearest Neighbor; LGBM, light gradient boosting machine; LR, logistic regression; RF, random forest; SVM, support vector machine; XGB, eXtreme gradient boosting.

**Table S22. Performance of machine learning algorithms (Predicting in-hospital mortality for patients admitted to the ICU)**

| Class | Model | AUC (95%CI) | Accuracy | Sensitivity | Specificity | PPV | NPV | F1 score |
| --- | --- | --- | --- | --- | --- | --- | --- | --- |
| Training set | KNN | 1.000 (1.000−1.000) | 1.000 | 1.000 | 1.000 | 1.000 | 1.000 | 1.000 |
| Training set | LGBM | 0.979 (0.966−0.991) | 0.929 | 0.942 | 0.916 | 0.590 | 0.992 | 0.726 |
| Training set | LR | 0.964 (0.945−0.983) | 0.898 | 0.962 | 0.835 | 0.427 | 0.994 | 0.592 |
| Training set | RF | 1.000 (1.000−1.000) | 1.000 | 1.000 | 1.000 | 1.000 | 1.000 | 1.000 |
| Training set | SVM | 0.954 (0.930−0.978) | 0.879 | 0.808 | 0.951 | 0.677 | 0.975 | 0.737 |
| Training set | XGB | 0.999 (0.998−1.000) | 0.995 | 1.000 | 0.990 | 0.929 | 1.000 | 0.963 |
| Validation set | KNN | 0.818 (0.720−0.916) | 0.703 | 0.458 | 0.948 | 0.579 | 0.919 | 0.512 |
| Validation set | LGBM | 0.885 (0.812−0.959) | 0.783 | 0.708 | 0.858 | 0.436 | 0.950 | 0.540 |
| Validation set | LR | 0.918 (0.860−0.976) | 0.838 | 0.875 | 0.800 | 0.404 | 0.976 | 0.553 |
| Validation set | RF | 0.938 (0.898−0.977) | 0.710 | 0.458 | 0.961 | 0.647 | 0.920 | 0.537 |
| Validation set | SVM | 0.934 (0.878−0.989) | 0.872 | 0.833 | 0.910 | 0.588 | 0.972 | 0.690 |
| Validation set | XGB | 0.911 (0.858−0.965) | 0.840 | 0.750 | 0.929 | 0.621 | 0.960 | 0.679 |

ICU, intensive care unit; AUC, area under the curve; CI, confidence interval; PPV, pos pred value; NPV, neg pred value; KNN, K-Nearest Neighbor; LGBM, light gradient boosting machine; LR, logistic regression; RF, random forest; SVM, support vector machine; XGB, eXtreme gradient boosting.

**Table S23. Performance of machine learning algorithms (Predicting re-admission within 30 days after discharge for patients admitted to the ICU)**

| Class | Model | AUC (95%CI) | Accuracy | Sensitivity | Specificity | PPV | NPV | F1 score |
| --- | --- | --- | --- | --- | --- | --- | --- | --- |
| Training set | KNN | 1.000 (1.000−1.000) | 1.000 | 1.000 | 1.000 | 1.000 | 1.000 | 1.000 |
| Training set | LGBM | 0.940 (0.906−0.973) | 0.895 | 0.963 | 0.828 | 0.260 | 0.997 | 0.409 |
| Training set | LR | 0.891 (0.834−0.949) | 0.856 | 0.963 | 0.749 | 0.194 | 0.997 | 0.323 |
| Training set | RF | 1.000 (1.000−1.000) | 1.000 | 1.000 | 1.000 | 1.000 | 1.000 | 1.000 |
| Training set | SVM | 0.747 (0.650−0.845) | 0.727 | 0.741 | 0.714 | 0.140 | 0.978 | 0.235 |
| Training set | XGB | 0.935 (0.898−0.972) | 0.868 | 0.889 | 0.847 | 0.267 | 0.992 | 0.410 |
| Validation set | KNN | 0.468 (0.390−0.546) | 0.470 | 0.000 | 0.940 | 0.000 | 0.929 | NaN |
| Validation set | LGBM | 0.517 (0.348−0.687) | 0.434 | 0.083 | 0.784 | 0.027 | 0.923 | 0.041 |
| Validation set | LR | 0.647 (0.479−0.815) | 0.568 | 0.417 | 0.719 | 0.096 | 0.945 | 0.156 |
| Validation set | RF | 0.665 (0.503−0.827) | 0.497 | 0.000 | 0.994 | 0.000 | 0.933 | NaN |
| Validation set | SVM | 0.530 (0.353−0.707) | 0.469 | 0.250 | 0.689 | 0.055 | 0.927 | 0.090 |
| Validation set | XGB | 0.559 (0.357−0.761) | 0.616 | 0.417 | 0.814 | 0.139 | 0.951 | 0.208 |

ICU, intensive care unit; AUC, area under the curve; CI, confidence interval; PPV, pos pred value; NPV, neg pred value; KNN, K-Nearest Neighbor; LGBM, light gradient boosting machine; LR, logistic regression; RF, random forest; SVM, support vector machine; XGB, eXtreme gradient boosting.

**Table S24. Performance of machine learning algorithms (Predicting re-admission within 60 days after discharge for patients admitted to the ICU)**

| Class | Model | AUC (95%CI) | Accuracy | Sensitivity | Specificity | PPV | NPV | F1 score |
| --- | --- | --- | --- | --- | --- | --- | --- | --- |
| Training set | KNN | 1.000 (1.000−1.000) | 1.000 | 1.000 | 1.000 | 1.000 | 1.000 | 1.000 |
| Training set | LGBM | 0.897 (0.852−0.942) | 0.841 | 0.895 | 0.788 | 0.276 | 0.988 | 0.422 |
| Training set | LR | 0.808 (0.733−0.882) | 0.753 | 0.684 | 0.821 | 0.257 | 0.966 | 0.374 |
| Training set | RF | 1.000 (1.000−1.000) | 1.000 | 1.000 | 1.000 | 1.000 | 1.000 | 1.000 |
| Training set | SVM | 0.712 (0.625−0.800) | 0.667 | 0.868 | 0.465 | 0.128 | 0.975 | 0.224 |
| Training set | XGB | 0.904 (0.855−0.954) | 0.833 | 0.868 | 0.797 | 0.280 | 0.985 | 0.423 |
| Validation set | KNN | 0.604 (0.482−0.726) | 0.527 | 0.167 | 0.888 | 0.143 | 0.905 | 0.154 |
| Validation set | LGBM | 0.675 (0.564−0.787) | 0.558 | 0.389 | 0.727 | 0.137 | 0.914 | 0.203 |
| Validation set | LR | 0.722 (0.603−0.841) | 0.663 | 0.556 | 0.770 | 0.213 | 0.939 | 0.308 |
| Validation set | RF | 0.756 (0.655−0.858) | 0.494 | 0.000 | 0.988 | 0.000 | 0.898 | NaN |
| Validation set | SVM | 0.689 (0.540−0.839) | 0.597 | 0.778 | 0.416 | 0.130 | 0.944 | 0.222 |
| Validation set | XGB | 0.753 (0.645−0.860) | 0.666 | 0.611 | 0.720 | 0.196 | 0.943 | 0.297 |

ICU, intensive care unit; AUC, area under the curve; CI, confidence interval; PPV, pos pred value; NPV, neg pred value; KNN, K-Nearest Neighbor; LGBM, light gradient boosting machine; LR, logistic regression; RF, random forest; SVM, support vector machine; XGB, eXtreme gradient boosting.

**Table S25. Performance of machine learning algorithms (Predicting re-admission within 90 days after discharge for patients admitted to the ICU)**

| Class | Model | AUC (95%CI) | Accuracy | Sensitivity | Specificity | PPV | NPV | F1 score |
| --- | --- | --- | --- | --- | --- | --- | --- | --- |
| Training set | KNN | 1.000 (1.000−1.000) | 1.000 | 1.000 | 1.000 | 1.000 | 1.000 | 1.000 |
| Training set | LGBM | 0.875 (0.817−0.933) | 0.817 | 0.750 | 0.884 | 0.407 | 0.971 | 0.528 |
| Training set | LR | 0.829 (0.756−0.902) | 0.777 | 0.705 | 0.850 | 0.333 | 0.964 | 0.453 |
| Training set | RF | 1.000 (1.000−1.000) | 1.000 | 1.000 | 1.000 | 1.000 | 1.000 | 1.000 |
| Training set | SVM | 0.807 (0.731−0.884) | 0.756 | 0.682 | 0.831 | 0.300 | 0.961 | 0.417 |
| Training set | XGB | 0.914 (0.869−0.960) | 0.856 | 0.841 | 0.872 | 0.411 | 0.981 | 0.552 |
| Validation set | KNN | 0.668 (0.551−0.784) | 0.581 | 0.238 | 0.924 | 0.294 | 0.901 | 0.263 |
| Validation set | LGBM | 0.778 (0.675−0.880) | 0.718 | 0.619 | 0.816 | 0.310 | 0.942 | 0.413 |
| Validation set | LR | 0.743 (0.638−0.847) | 0.654 | 0.524 | 0.785 | 0.244 | 0.925 | 0.333 |
| Validation set | RF | 0.753 (0.646−0.861) | 0.494 | 0.000 | 0.987 | 0.000 | 0.881 | NaN |
| Validation set | SVM | 0.690 (0.561−0.819) | 0.672 | 0.571 | 0.772 | 0.250 | 0.931 | 0.348 |
| Validation set | XGB | 0.733 (0.622−0.843) | 0.654 | 0.524 | 0.785 | 0.244 | 0.925 | 0.333 |

ICU, intensive care unit; AUC, area under the curve; CI, confidence interval; PPV, pos pred value; NPV, neg pred value; KNN, K-Nearest Neighbor; LGBM, light gradient boosting machine; LR, logistic regression; RF, random forest; SVM, support vector machine; XGB, eXtreme gradient boosting.

**Table S26. Performance of machine learning algorithms (Predicting mortality within 180 days after discharge for patients admitted to the ICU)**

| Class | Model | AUC (95%CI) | Accuracy | Sensitivity | Specificity | PPV | NPV | F1 score |
| --- | --- | --- | --- | --- | --- | --- | --- | --- |
| Training set | KNN | 1.000 (1.000−1.000) | 1.000 | 1.000 | 1.000 | 1.000 | 1.000 | 1.000 |
| Training set | LGBM | 0.888 (0.837−0.940) | 0.821 | 0.846 | 0.797 | 0.280 | 0.982 | 0.420 |
| Training set | LR | 0.833 (0.773−0.892) | 0.762 | 0.718 | 0.806 | 0.257 | 0.968 | 0.378 |
| Training set | RF | 1.000 (1.000−1.000) | 1.000 | 1.000 | 1.000 | 1.000 | 1.000 | 1.000 |
| Training set | SVM | 0.771 (0.699−0.844) | 0.707 | 0.872 | 0.543 | 0.151 | 0.978 | 0.258 |
| Training set | XGB | 0.913 (0.875−0.952) | 0.837 | 0.923 | 0.751 | 0.257 | 0.991 | 0.402 |
| Validation set | KNN | 0.446 (0.364−0.529) | 0.470 | 0.000 | 0.917 | 0.000 | 0.933 | NaN |
| Validation set | LGBM | 0.649 (0.494−0.803) | 0.627 | 0.545 | 0.708 | 0.109 | 0.960 | 0.182 |
| Validation set | LR | 0.745 (0.624−0.865) | 0.766 | 0.818 | 0.714 | 0.158 | 0.984 | 0.265 |
| Validation set | RF | 0.660 (0.513−0.807) | 0.531 | 0.091 | 0.970 | 0.167 | 0.942 | 0.118 |
| Validation set | SVM | 0.682 (0.534−0.830) | 0.656 | 0.818 | 0.494 | 0.096 | 0.976 | 0.171 |
| Validation set | XGB | 0.728 (0.603−0.852) | 0.627 | 0.545 | 0.708 | 0.109 | 0.960 | 0.182 |

ICU, intensive care unit; AUC, area under the curve; CI, confidence interval; PPV, pos pred value; NPV, neg pred value; KNN, K-Nearest Neighbor; LGBM, light gradient boosting machine; LR, logistic regression; RF, random forest; SVM, support vector machine; XGB, eXtreme gradient boosting.

**Table S27. Performance of machine learning algorithms (Predicting mortality within 365 days after discharge for patients admitted to the ICU)**

| Class | Model | AUC (95%CI) | Accuracy | Sensitivity | Specificity | PPV | NPV | F1 score |
| --- | --- | --- | --- | --- | --- | --- | --- | --- |
| Training set | KNN | 1.000 (1.000−1.000) | 1.000 | 1.000 | 1.000 | 1.000 | 1.000 | 1.000 |
| Training set | LGBM | 0.849 (0.795−0.903) | 0.780 | 0.860 | 0.700 | 0.261 | 0.976 | 0.400 |
| Training set | LR | 0.798 (0.733−0.863) | 0.737 | 0.680 | 0.794 | 0.288 | 0.953 | 0.405 |
| Training set | RF | 1.000 (1.000−1.000) | 1.000 | 1.000 | 1.000 | 1.000 | 1.000 | 1.000 |
| Training set | SVM | 0.679 (0.600−0.758) | 0.633 | 0.500 | 0.767 | 0.208 | 0.926 | 0.294 |
| Training set | XGB | 0.880 (0.833−0.928) | 0.808 | 0.800 | 0.816 | 0.348 | 0.971 | 0.485 |
| Validation set | KNN | 0.478 (0.350−0.607) | 0.476 | 0.083 | 0.868 | 0.043 | 0.929 | 0.057 |
| Validation set | LGBM | 0.569 (0.385−0.754) | 0.573 | 0.500 | 0.647 | 0.092 | 0.947 | 0.156 |
| Validation set | LR | 0.669 (0.512−0.826) | 0.624 | 0.583 | 0.665 | 0.111 | 0.957 | 0.187 |
| Validation set | RF | 0.624 (0.464−0.785) | 0.553 | 0.167 | 0.940 | 0.167 | 0.940 | 0.167 |
| Validation set | SVM | 0.668 (0.513−0.823) | 0.612 | 0.500 | 0.725 | 0.115 | 0.953 | 0.188 |
| Validation set | XGB | 0.712 (0.578−0.845) | 0.606 | 0.500 | 0.713 | 0.111 | 0.952 | 0.182 |

ICU, intensive care unit; AUC, area under the curve; CI, confidence interval; PPV, pos pred value; NPV, neg pred value; KNN, K-Nearest Neighbor; LGBM, light gradient boosting machine; LR, logistic regression; RF, random forest; SVM, support vector machine; XGB, eXtreme gradient boosting.

**Table S28. Performance of clinical scores**

| Class | Model | AUC (95%CI) | Accuracy | Sensitivity | Specificity | PPV | NPV | F1 score |
| --- | --- | --- | --- | --- | --- | --- | --- | --- |
| Training set | APSIII | 0.844 (0.791−0.897) | 0.769 | 0.885 | 0.654 | 0.247 | 0.978 | 0.387 |
| Training set | SAPSII | 0.834 (0.778−0.891) | 0.765 | 0.865 | 0.664 | 0.249 | 0.975 | 0.386 |
| Training set | SIRS | 0.590 (0.518−0.661) | 0.576 | 0.865 | 0.286 | 0.135 | 0.943 | 0.233 |
| Training set | SOFA | 0.770 (0.702−0.838) | 0.705 | 0.750 | 0.659 | 0.220 | 0.954 | 0.341 |
| Validation set | APSIII | 0.859 (0.789−0.929) | 0.782 | 0.958 | 0.606 | 0.274 | 0.989 | 0.426 |
| Validation set | SAPSII | 0.839 (0.770−0.908) | 0.770 | 0.875 | 0.665 | 0.288 | 0.972 | 0.433 |
| Validation set | SIRS | 0.660 (0.563−0.756) | 0.629 | 0.917 | 0.342 | 0.177 | 0.964 | 0.297 |
| Validation set | SOFA | 0.814 (0.719−0.909) | 0.741 | 0.792 | 0.690 | 0.284 | 0.955 | 0.418 |

AUC, area under the curve; CI, confidence interval; PPV, pos pred value; NPV, neg pred value; APS III, Acute Physiology Score III; SAPS II, Simplified Acute Physiology Score II; SIRS, Systemic Inflammatory Response Syndrome Criteria; SOFA, Sequential Organ Failure Assessment.

**Table S29. Variables of patients underwent biliary drainage during ICU treatment in eICU database (n = 617) and Wenzhou Hospital (n = 150)**

| Variables | n (%), mean ± SD (range), or med [IQR] | |
| --- | --- | --- |
|  | eICU database (n = 617) | Wenzhou Hospital (n = 150) |
| Death in hospital, n (%) | 23 (3.75%) | 16 (10.67%) |
| Age (years) | 52.00 [41.00-64.00] | 50.00 [39.25-70.00] |
| Bicarbonate _ avg (mEq/L) | 24.50 [21.58-26.75] | 21.25 (5.18) |
| Creatinine _ max (mg/dL) | 1.00 [0.71-1.71] | 1.33 [0.92-3.06] |
| Glucose _ avg (mg/dL) | 131.40 [106.0-172.07] | 174.28 [145.15-237.37] |
| Potassium _ avg (mEq/L) | 3.80 [3.60-4.06] | 3.83 [3.65-4.03] |
| Urea nitrogen _ avg (mg/dL) | 13.00 [7.38-23.35] | 36.42 [24.51-67.29] |
| Hemoglobin _ min (g/dL) | 10.30 [8.40-11.80] | 8.70 [6.80-10.60] |
| MCHC _ min (g/dL) | 32.60 [31.74-33.50] | 29.50 [28.30-30.70] |
| MCV _ max (fL) | 92.00 [87.27-96.40] | 94.20 [90.73-97.68] |
| PLT _ max (K/μL) | 229.75 [160.00-334.00] | 294.50 [198.00-436.75] |
| PLT _ avg (K/μL) | 193.88 [139.38-259.28] | 201.18 [143.20-262.29] |
| RDW _ avg (%) | 14.26 [13.25-15.40] | 14.06 [13.25-14.91] |
| WBC _ min (K/μL) | 7.40 [5.30-10.10] | 6.17 [4.15-8.83] |
| TBil _ min (mg/dL) | 0.60 [0.30-1.00] | 0.70 [0.47-1.17] |
| AST _max (IU/L) | 59.00 [27.00-137.12] | 75.50 [41.25-182.50] |
| AST _min (IU/L) | 9.00 [5.00-16.00] | 23.50 [17.00-34.00] |
| Phosphate _ max (mg/dL) | 2.50 [0.00-3.90] | 3.34 [2.68-4.24] |
| Phosphate _ avg (mg/dL) | 2.20 [0.00-3.15] | 2.76 [2.20-3.35] |
| Sepsis, n (%) | 62 (10.05%) | 15 (10.00%) |
| AKI, n (%) | 104 (16.86%) | 48 (32.00%) |
| Heart rate on the first day of ICU _ max (beats/minute) | 99.39 [81.00-115.71] | 121.50 [105.00-138.00] |
| Heart rate on the first day of ICU _ avg (beats/minute) | 91.53 [74.47-107.00] | 103.76 (21.34) |
| SysBP on the first day of ICU _ max (mmHg) | 130.00 [111.00-148.00] | 152.00 [136.00-165.75] |
| SysBP on the first day of ICU _ min (mmHg) | 119.12 [101.00-136.00] | 107.00 [94.00-122.50] |
| Respiratory rate on the first day of ICU _ max (beats/mins) | 20.00 [17.0-24.33] | 29.00 [24.25-35.00] |
| SpO2 on the first day of ICU _ max (%) | 96.00 [93.69-98.00] | 98.20 [95.20-99.00] |
| SpO2 on the first day of ICU _ min (%) | 94.56 [92.0-96.43] | 95.70 [90.30-97.80] |

MCHC, mean corpuscular hemoglobin concentration; MCV, mean corpuscular volume; PLT, platelet count; RDW, red cell distribution width; WBC, white blood cells; TBil, total bilirubin; AST, aspartate aminotransferase; AKI, acute kidney injury; ICU, intensive care unit.

**Table S30. Performance of machine learning algorithms (Predicting in-hospital mortality for patients admitted to the ICU) in two external validation sets**

| Class | Model | AUC (95%CI) | Accuracy | Sensitivity | Specificity | PPV | NPV | F1 score |
| --- | --- | --- | --- | --- | --- | --- | --- | --- |
| eICU database | KNN | 0.703 (0.589−0.817) | 0.635 | 0.478 | 0.791 | 0.081 | 0.975 | 0.139 |
| eICU database | LGBM | 0.921 (0.840−1.000) | 0.765 | 0.565 | 0.965 | 0.382 | 0.983 | 0.456 |
| eICU database | LR | 0.877 (0.812−0.943) | 0.779 | 0.696 | 0.862 | 0.163 | 0.987 | 0.264 |
| eICU database | RF | 0.883 (0.812−0.954) | 0.640 | 0.304 | 0.976 | 0.333 | 0.973 | 0.318 |
| eICU database | SVM | 0.806 (0.749−0.863) | 0.683 | 0.565 | 0.801 | 0.099 | 0.979 | 0.169 |
| eICU database | XGB | 0.931 (0.873−0.988) | 0.784 | 0.609 | 0.960 | 0.368 | 0.984 | 0.459 |
| Wenzhou Hospital | KNN | 0.695 (0.565−0.826) | 0.591 | 0.250 | 0.933 | 0.308 | 0.912 | 0.276 |
| Wenzhou Hospital | LGBM | 0.868 (0.777−0.959) | 0.733 | 0.562 | 0.903 | 0.409 | 0.945 | 0.474 |
| Wenzhou Hospital | LR | 0.748 (0.599−0.896) | 0.672 | 0.500 | 0.843 | 0.276 | 0.934 | 0.356 |
| Wenzhou Hospital | RF | 0.837 (0.738−0.936) | 0.711 | 0.438 | 0.985 | 0.778 | 0.936 | 0.560 |
| Wenzhou Hospital | SVM | 0.800 (0.682−0.917) | 0.689 | 0.438 | 0.940 | 0.467 | 0.933 | 0.452 |
| Wenzhou Hospital | XGB | 0.811 (0.674−0.948) | 0.725 | 0.562 | 0.888 | 0.375 | 0.944 | 0.450 |

ICU, intensive care unit; AUC, area under the curve; CI, confidence interval; PPV, pos pred value; NPV, neg pred value; KNN, K-Nearest Neighbor; LGBM, light gradient boosting machine; LR, logistic regression; RF, random forest; SVM, support vector machine; XGB, eXtreme gradient boosting.
